# Evidence Map of Pharmacologic and Non-Pharmacologic Perioperative Strategies for Managing Acute Postoperative Pain After Laparoscopic Surgery, 2012–2025: The M-PALS Collaborative

**DOI:** 10.3390/jcm15082872

**Published:** 2026-04-10

**Authors:** Romil R. Parikh, Gabriella L. Lott, Miranda Considine, Peter Sawtell, Sallee Brandt, Luz Angela Choconta-Piraquive, Swathi Pagadala, Drew J. Persson, Amy M. Claussen, Christopher J. Tignanelli, Timothy Wilt, Shahnaz Sultan, Adalyn J. Scherer, Aaron Berg, Christie L. Martin, Elizabeth Wick, Genevieve B. Melton, Mary E. Butler, Bronwyn J. Southwell

**Affiliations:** 1 School of Public Health, University of Minnesota, Minneapolis, MN 55455, USA; 2 Center for Learning Health System Sciences, University of Minnesota, Minneapolis, MN 55455, USA; 3 “Twin Cities”, University of Minnesota Medical School, Minneapolis, MN 55455, USA; 4 Department of Surgery, University of Minnesota, Minneapolis, MN 55455, USA; 5 Institute for Health Informatics, University of Minnesota, Minneapolis, MN 55455, USA; 6 Veterans Association Health Care System, Minneapolis, MN 55417, USA; 7 Department of Medicine, University of Minnesota, Minneapolis, MN 55455, USA; 8 Department of Anesthesiology, University of Minnesota, Minneapolis, MN 55455, USA; 9 School of Nursing, University of Minnesota, Minneapolis, MN 55455, USA; 10 Department of Surgery, University of California, San Francisco, CA 94143, USA

**Keywords:** postoperative pain, acute pain, opioid use, regional anesthesia, analgesia

## Abstract

**Background:** Effectively managing acute postoperative pain after laparoscopic surgery (M-PALS) is essential to optimize outcomes, enhance recovery, and mitigate opioid-related risks. We aimed to systematically map evidence on effectiveness and harms of pharmacologic and non-pharmacologic interventions for M-PALS. **Methods:** We searched three databases (2012–2025) for randomized clinical trials (RCTs) that reported postoperative opioid use and pain-related outcomes. We assessed study quality using the Cochrane Risk of Bias (ROB)-2 tool. **Results:** From 7638 citations, we included 101 RCTs. Postoperative opioid use was reported variably (e.g., total use over 24 or 48 h postoperatively, frequency of rescue-opioid use, and time to first rescue-opioid use). One out of 101 RCTs evaluated opioid prescription at discharge. No RCT reported opioid use at ≥3 months postoperatively. Eleven strategies were evaluated in ≥2 RCTs, with usual care/ sham as comparators. None of the 101 RCTs favored usual care over any intervention for pain or opioid use outcomes. For regional anesthesia (21 RCTs total; 12 with low ROB), intraperitoneal/preperitoneal local anesthetic instillation (10 RCTs; 4 with low ROB), intravenous dexamethasone (3 RCTs; 1 with low ROB), and the Enhanced Recovery After Surgery (ERAS) protocol (3 RCTs; 0 with low ROB), compared to usual care, >50% of RCTs favored the intervention for reducing pain and opioid use. For adverse events, only 3 out of 101 RCTs favored comparators. Inconsistent outcome reporting across all RCTs and, for multimodal strategies, the uniqueness of intervention–comparator combinations hindered comparisons. **Conclusions:** Interventions for M-PALS appear safe, with no RCT indicating worse efficacy of intervention than usual care; but evidence regarding superiority is conflicting. Future research should establish standardized and longer-term core outcome sets and make head-to-head comparisons between optimal strategies.

## 1. Introduction

Minimally invasive abdominal and pelvic laparoscopic surgery offers advantages over open procedures, including reduced postoperative pain, shorter recovery, and decreased hospital length of stay (LOS) [1,2]. However, many patients still experience substantial postoperative pain that can impair recovery and quality of life, underscoring the need for effective pain management strategies [2,3]. Opioids remain central to postoperative analgesia, yet their overuse, excess prescription, and/or resultant unused opioid medication contributes to dependence, diversion, and overdose [3,4,5,6]. The U.S. Food and Drug Administration (FDA) has urged clinicians to minimize unnecessary opioid exposure, particularly in the perioperative setting, as unused opioid prescriptions frequently contribute to community misuse [7,8]. Under the SUPPORT Act, the FDA commissioned the National Academies of Sciences, Engineering, and Medicine (NASEM) to develop clinical practice guidelines for acute pain management [8,9]. NASEM identified a lack of evidence-based guidance specific to laparoscopic abdominal surgery [9]. To address this gap, the FDA sponsored the *Managing Pain After Laparoscopic Surgery* (M-PALS) initiative which included a review and appraisal of the evidence on this topic. Consistent with the objectives of evidence mapping methodology, we present here an evidence map of strategies for M-PALS to identify patterns and gaps in evidence, without determining relative comparative effectiveness nor endorsing the superiority of any strategy. This evidence map emphasizes longer-term postoperative outcomes, at ≥24 h postoperatively.

## 2. Methods

This evidence map was prepared following the Agency for Healthcare Research and Quality Methods Guide and the Preferred Reporting Items for Systematic Reviews and Meta-Analyses (PRISMA) guidance [10,11]. The protocol was prospectively registered in PROSPERO (CRD42024569937). Following the objectives for evidence maps, we did not perform a formal meta-analysis or strength-of-evidence assessment [12,13,14]. Methods are detailed in the online-only supplement [Supplement SI] and summarized below.

### 2.1. Key Question

The primary objective was to identify, categorize, and visually summarize evidence on pain management strategies aimed at reducing opioid use and improving patient-reported pain among adults undergoing laparoscopic or robotic abdominal or pelvic surgery.

#### Data Sources and Study Selection

We conducted a comprehensive search of MEDLINE, Embase, and Scopus for English-language publications from 1 January 2012 through 30 January 2025, and queried ClinicalTrials.gov for ongoing or unpublished studies. The search strategy for databases is available in supplemental methods [Supplement SIB]. We determined the study publication date cutoff to be 2012 based on the widespread increase in multimodal, enhanced recovery regimens after that time creating a difference in the baseline perioperative and analgesic care that patients received. Secondly, 2012 was the peak of the opioid misuse epidemic in the United States and signaled a shift in pain management with the goal of minimizing opioids, as well as study designs to better detect a decrease in opioid consumption [14]. We screened titles and abstracts using PICO Portal™ (https://picoportal.org/ St. Petersburg, FL, USA; last accessed on 30 March 2026), a machine-learning enabled platform (details in Supplementary Material) [15]. Included studies were randomized controlled trials (RCTs) of adults undergoing abdominal or pelvic, laparoscopic, or robotic surgery, reporting at least one pain and one postoperative opioid outcome, with ≥50 participants per arm (details in Supplementary SIC and Table S1).

### 2.2. Data Extraction

Data abstraction followed the Template for Intervention Description and Replication (TIDieR) checklist [16], capturing study characteristics, patient demographics, interventions, comparators, and outcomes including patient-reported pain and postoperative opioid consumption at 24, 48, and >48 h postoperatively; rescue opioid metrics (for example, frequency of and time to first rescue dose), hospital LOS, quality of life, patient satisfaction, readmissions, and adverse events [Supplementary SID].

### 2.3. Data Analysis and Evidence Synthesis

We used the Cochrane Risk of Bias (ROB) 2.0 Tool [17] to assess study quality by dual independent review [Supplementary SIE]. Ratings were assigned as low, some concerns (moderate), or high for each RCT, with disagreements resolved by team consensus. Evidence synthesis was descriptive and visual. We used tableau (Salesforce Inc., San Francisco, CA, USA) to summarize study distributions and for data visualization [18]. Graphs included only interventions evaluated in two or more RCTs [Supplementary SIF]. Heat maps were generated to display the number of RCTs reporting a given outcome for each intervention type. Bubble plots were generated to map the signal for effectiveness and harms of interventions along with the ROB and sample size of RCTs.

## 3. Results

From 7638 citations, we screened 408 full-text articles of which 101 eligible RCTs [19,20,21,22,23,24,25,26,27,28,29,30,31,32,33,34,35,36,37,38,39,40,41,42,43,44,45,46,47,48,49,50,51,52,53,54,55,56,57,58,59,60,61,62,63,64,65,66,67,68,69,70,71,72,73,74,75,76,77,78,79,80,81,82,83,84,85,86,87,88,89,90,91,92,93,94,95,96,97,98,99,100,101,102,103,104,105,106,107,108,109,110,111,112,113,114,115,116,117,118,119] were included (Supplement SIG; Figure S1). Only 11 interventions were evaluated in ≥2 eligible RCTs, namely, regional anesthesia (24 RCTs), intraperitoneal/preperitoneal local anesthetic [LA] instillation (10 RCTs), combination of intra- or preperitoneal LA and incisional LA (2 RCTs), incisional LA (7 RCTs), gabapentin (3 RCTs), intravenous dexamethasone (3 RCTs), acetaminophen (2 RCTs), non-steroidal anti-inflammatory drugs (NSAIDs; 3 RCTs), active removal of insufflation gas (13 RCTs), use of warm humidified insufflation gas (3 RCTs) and the Enhanced Recovery After Surgery (ERAS) protocol (3 RCTs). Other strategies evaluated in single RCTs included multimodal strategies with unique combinations (7 RCTs), complementary and alternative medicine strategies (7 RCTs), and other single strategies (14 RCTs).

**Figure 1:**
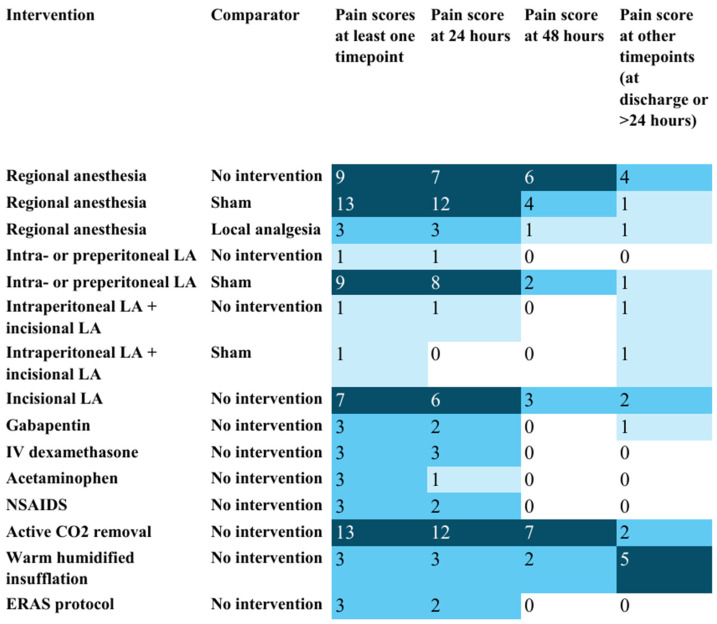
Heat map showing the number of studies reporting outcomes related to patient-reported postoperative pain scores: evidence map for the management of pain after laparoscopic surgery (M-PALS). Note: CO_2_—carbon dioxide; ERAS—Enhanced Recovery After Surgery; LA—local anesthetics. The counts in each cell represent the number of randomized controlled trials reporting the outcome mentioned in the column heading for the intervention–comparator pair mentioned in the row heading. For example, 12 randomized controlled trials reported pain at 24 h as an outcome for regional anesthesia versus sham. This information is presented to help plan future systematic reviews. Darker shades of blue-colored cells represent a greater number of studies in those cells (i.e. larger clustering of evidence).

**Figure 2:**
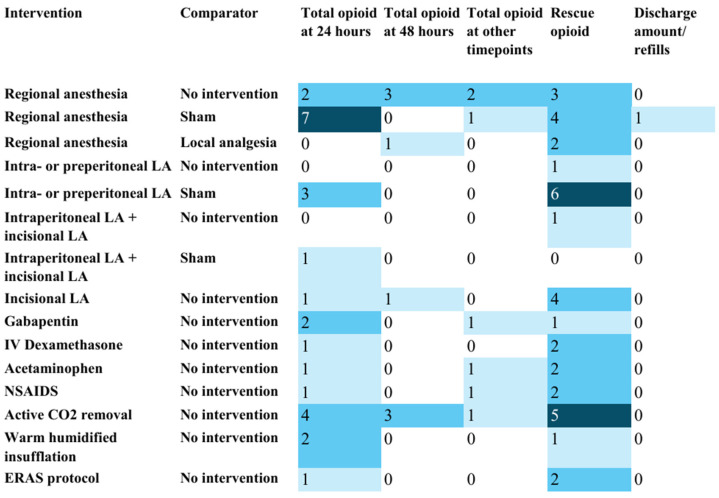
Heat map showing the number of studies reporting outcomes related to postoperative opioid use: evidence map for the management of pain after laparoscopic surgery (M-PALS). Note: CO_2_—carbon dioxide; ERAS—Enhanced Recovery After Surgery; LA—local anesthetics. The counts in each cell represent the number of randomized controlled trials reporting the outcome mentioned in the column heading for the intervention–comparator pair mentioned in the row heading. For example, seven randomized controlled trials reported total postoperative opioid use at 24 h as an outcome for regional anesthesia versus sham. This information is presented to help plan future systematic reviews. Darker shades of blue-colored cells represent a greater number of studies in those cells (i.e. larger clustering of evidence).

**Figure 3:**
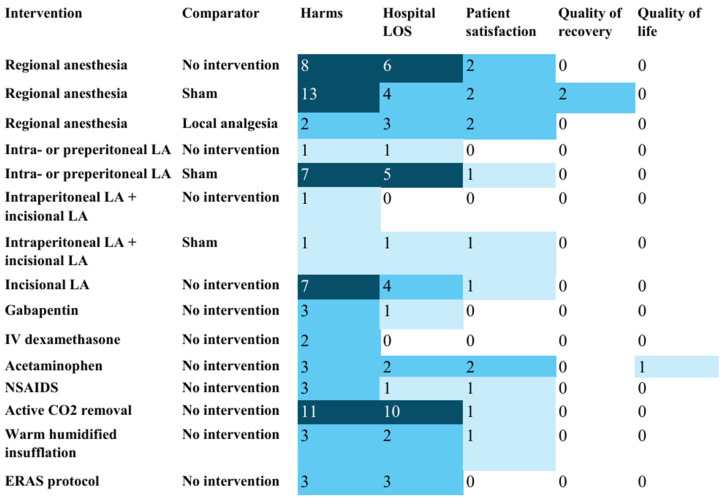
Heat map showing the number of studies reporting harms and other outcomes: evidence map for the management of pain after laparoscopic surgery (M-PALS). Note: CO_2_—carbon dioxide; ERAS—Enhanced Recovery After Surgery; LA—local anesthetics. The counts in each cell represent the number of randomized controlled trials reporting the outcome mentioned in the column heading for the intervention–comparator pair mentioned in the row heading. For example, 13 randomized controlled trials reported harms as an outcome for regional anesthesia versus sham. This information is presented to help plan future systematic reviews. Darker shades of blue-colored cells represent a greater number of studies in those cells (i.e. larger clustering of evidence).

Outcome reporting was inconsistent across RCTs (Figure 1, Figure 2 and Figure 3). Patient-reported pain score at 24 h postoperatively was the most frequently reported outcome. Reporting of postoperative opioid use was inconsistent, with several RCTs not reporting total opioid use and reporting disparate rescue opioid metrics (for example, time to first rescue dose, number of patients requiring a rescue dose, or mean number of clicks on patient-controlled pumps for rescue dose). Only one RCT evaluated opioid prescription at discharge. None of the RCTs reported long-term opioid use outcomes at three months or beyond.

**Figure 4:**
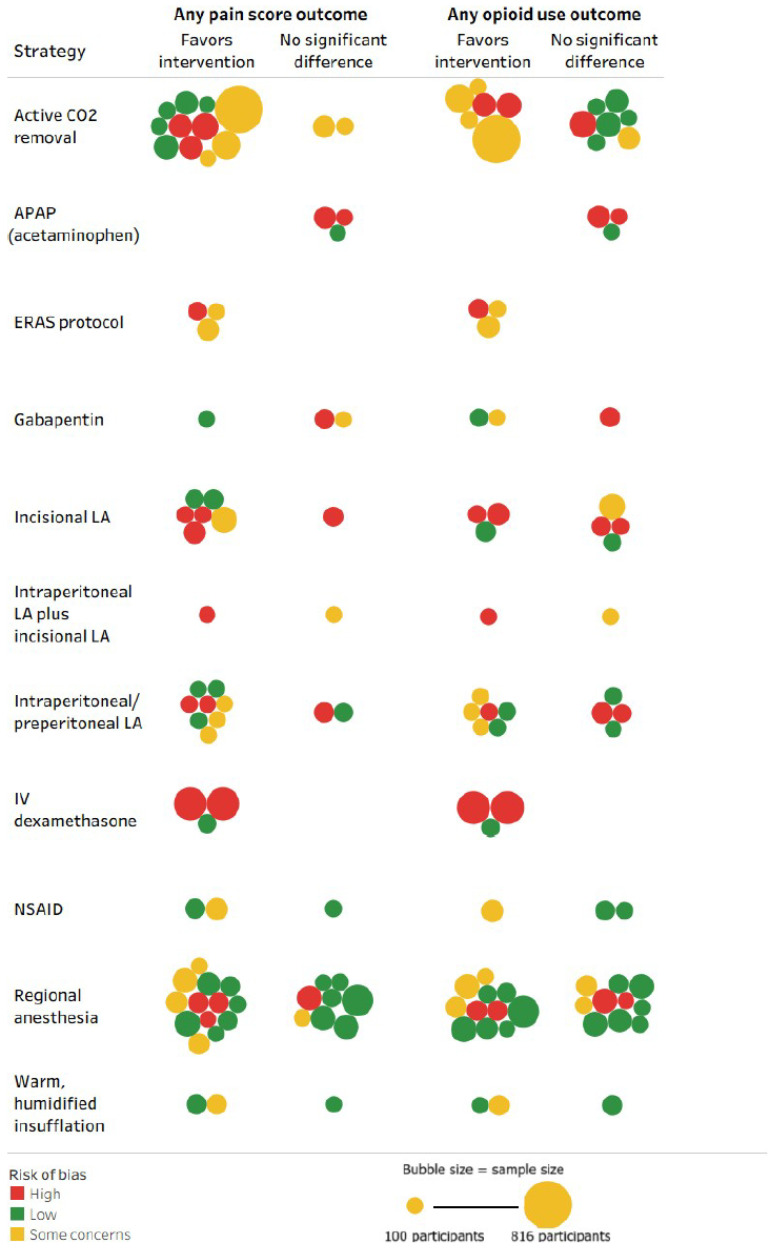
Bubble plots mapping the signal for any effect on patient-reported pain score and opioid use outcomes of strategies for the management of pain after laparoscopic surgery (M-PALS). Note: CO_2_—carbon dioxide; ERAS—Enhanced Recovery After Surgery; LA—local anesthetics. Each bubble represents a unique randomized controlled trial. The size of the bubble represents the relative number of participants randomized in the trial, ranging between 100 and 816 patients. The color of the bubble represents the ROB assessment for the study as shown in the legend; green represents low ROB, yellow represents moderate ROB, and red represents high ROB. The outcome column “any pain score outcome” includes any form of patient-reported postoperative pain outcome at any timepoint; an RCT would be categorized as “favors intervention” if it favored the intervention in at least one pain-related outcome, and would be classified as “no significant difference” only if it reported no significant difference for every pain outcome reported. Similarly, the column for “any opioid use outcome” includes any postoperative opioid use outcome; an RCT would be categorized as “favors intervention” if it favored the intervention in at least one postoperative opioid use-related outcome, and would be classified as “no significant difference” only if it reported no significant difference for every postoperative opioid use outcome reported. The intention of the bubble plots is to identify clustering of any signal of an effect for postoperative pain and opioid use. The bubble plot should not be used as a substitute for a formal systematic review to draw any conclusions regarding the comparative effectiveness of the interventions. No trials favored control (i.e., usual care or sham), no trial reported conflicting findings, and no trial failed to report the outcome.

**Figure 5:**
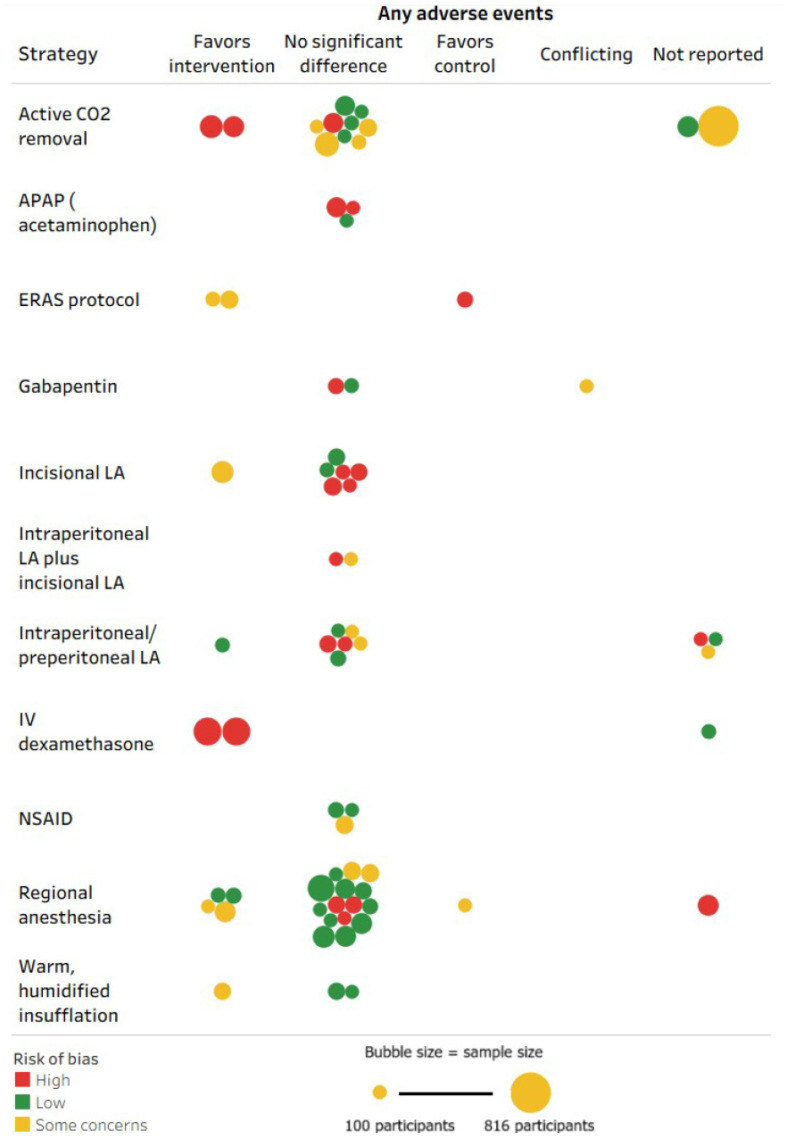
Bubble plots mapping the signal for any harm of strategies for the management of pain after laparoscopic surgery (M-PALS). Note: CO_2_—carbon dioxide; ERAS—Enhanced Recovery After Surgery; LA—local anesthetics. Each bubble represents a unique randomized controlled trial. The size of the bubble represents the relative number of participants randomized in the trial, ranging between 100 and 816 patients. The color of the bubble represents the ROB assessment for the study as shown in the legend; green represents low ROB, yellow represents moderate ROB, and red represents high ROB. The bubble plot should not be used as a substitute for a formal systematic review to draw any conclusions regarding the comparative effectiveness of the interventions.

Signals for effectiveness (based on pain scores and opioid use) and harms are mapped in the bubble plots (Figure 4 and Figure 5). For pain and opioid outcomes, the findings for all 11 interventions versus usual care/sham varied between favoring intervention and no significant difference. None of the included RCTs favored usual care over the intervention for pain and opioid outcomes. None of the included RCTs focused on or reported subgroup analyses for patients with history of chronic opioid use or other psychiatric comorbidities who were at higher risk of subsequent opioid misuse or dependency; several RCTs excluded these high-risk subpopulations. For adverse events, >50% RCTs in each of 11 strategies reported either no significant difference or favored the intervention; and only one RCT each in regional anesthesia, gabapentin, and ERAS protocol reported favoring usual care over intervention. For regional anesthesia, intraperitoneal/preperitoneal LA, dexamethasone, and ERAS protocol, compared to usual care or sham, >50% of the evaluating RCTs favored the intervention for both patient-reported pain reduction and postoperative opioid use metrics.

### 3.1. Regional Anesthesia

We identified 24 RCTs [19,20,21,22,23,24,25,26,27,28,29,30,31,32,33,34,35,36,37,38,39,40,41,42] (five with low ROB [23,27,32,35,40] and three with high ROB [22,24,38]) evaluating regional anesthesia interventions (Supplemental Tables SA.1–SA.9); all involved peripheral nerve (not neuraxial) blocks (as opposed to neuraxial blocks).

Regional anesthesia was compared with usual care in nine RCTs (n = 1226 patients) [22,23,24,27,32,35,38,40,42]. Three RCTs [24,27,38] focused on gynecologic surgery and the remaining six RCTs had ≥42% female participants. Mean age was 39–66 years. Mean BMI was 21–45 kg/m^2^, with two RCTs reporting a mean BMI > 30 kg/m^2^ (Supplemental Table SA.1). ROB was assessed as low in five RCTs [23,27,32,35,40], moderate in one RCT due to deviation from intended intervention [42], and high in three RCTs due to concerns about deviation from intended intervention, selective reporting of results, incompletely described randomization process, and missing data (Supplemental Table SA.2) [22,24,38].

Regional anesthesia was compared with sham block in 13 RCTs (n = 2193 patients) [19,20,21,24,25,26,28,29,31,36,37,39,41]. Six RCTs focused on gynecologic surgery [19,20,24,25,26,31]. The proportion of females was >38% in six RCTs [21,28,29,36,39,41] and not reported in one RCT [37]. Mean age was 32–72 years, with only one RCT reporting mean age >65 years [36]. Mean BMI was 24–45 kg/m^2^, with four RCTs reporting a mean BMI > 40 kg/m^2^ [21,37,39,41] and two RCTs not reporting mean BMI (Supplemental Table SA.4) [26,31]. ROB was assessed as low in seven RCTs [19,20,26,28,36,37,41], moderate in four RCTs [21,25,29,39], and high in two RCTs due to concerns about deviation from intended intervention, selective reporting of results, incompletely described randomization process, and missing data (Supplemental Table SA.5) [24,31].

Regional anesthesia was compared with incisional LA in three RCTs (n = 398 patients; one RCT with low ROB; conflicting findings; Supplemental Tables SA.7–SA.9) [30,33,34].

### 3.2. Intraperitoneal or Preperitoneal Instillation of Local Anesthetics

Ten RCTs (n = 1091 patients) [43,44,45,46,47,48,49,50,51,52] evaluated the efficacy of intraperitoneal (including one preperitoneal [45]) instillation of LA (Supplemental Tables SB.1–SB.6): nine versus sham (normal saline) [43,44,45,46,47,48,49,50,52] and one versus usual care [51]. Two involved gynecological surgery [44,45], one urological surgery [46], and seven gastrointestinal surgery (Supplemental Table SB.1). The proportion of females was ≥55% in nine RCTs and not reported in one RCT [50]. Mean/median age was 32–49 years (Supplemental Table SB.1). Mean/median BMI was >40 kg/m^2^ in six RCTs [43,47,49,50,51,52] and not reported in three RCTs [45,46,48]. Out of the 10 RCTs, five were assessed at low ROB [43,45,50,51,52], two at moderate ROB [46,49], and three at high ROB (Supplemental Table SB.2) [44,47,48].

Two RCTs (n = 208 patients; Supplemental Table SB.4) evaluated combined LA for intraperitoneal instillation plus infiltration of incisional sites versus normal saline (moderate ROB) or no intervention (high ROB; Supplemental Table SB.5) [53,54].

### 3.3. Incisional Local Anesthetics

We identified seven RCTs (n = 1056 patients) evaluating the efficacy of incisional LA versus sham or usual care (Supplemental Tables SC.1–SC.3) [55,56,57,58,59,60,61]. Sham was the comparator in two RCTs only [58,61]; therefore, we pooled findings from RCTs with sham or usual care comparators. Among the seven RCTs, two focused on laparoscopic gynecologic surgery [56,57] and five on laparoscopic gastrointestinal surgery (Supplemental Table SC.1). Mean age was 29 to 56 years. The proportion of females was >50% in six RCTs [55,56,57,58,59,60]. Mean BMI was 24 to 27 kg/m^2^ in five RCTs. BMI was not reported in two RCTs [57,61]. ROB was assessed as low in two RCTs [56,61], moderate in one RCT [59] and high in four RCTs (Supplemental Table SC.2) [55,57,58,60].

### 3.4. Gabapentinoids

Three RCTs (n = 357 patients) [62,63,64] evaluated oral gabapentin (Supplemental Tables SD.1–SD.3): two versus placebo (n = 220 patients; both RCTs with focus on gastrointestinal surgery) [63,64] and one versus usual care (focused on gynecologic surgery) [62]. Mean age was 36–53 years and the proportion of females was ≥75%. One RCT reported average BMI > 40 kg/m^2^ [64], one reported median BMI <30 kg/m^2^ [62], and one did not report BMI [63]. ROB was low in one RCT [64], moderate in one RCT [63], and high in one RCT [62].

### 3.5. Intravenous Dexamethasone

Three RCTs (n = 890 patients) evaluated intravenous dexamethasone versus normal saline for laparoscopic cholecystectomy (Supplemental Tables SE.1–SE.3) [65,66,67]. Mean age was 40–69 years and the proportion of females was ≥48%. Average BMI was not reported. ROB was assessed as low in one RCT [67] and high in two RCTs [65,66].

### 3.6. Acetaminophen

Three RCTs (n = 384 patients) evaluated intravenous acetaminophen versus normal saline for abdominal and pelvic laparoscopic procedures (Supplemental Tables SF.1–SF.3) [68,69,70]. Mean age was 41–62 years and the proportion of females was >93%. Average BMI was <30 kg/m^2^ in one RCT [70], >40 kg/m^2^ in another [68], and not reported in the third RCT [69]. ROB was assessed as low in one RCT [68] and high in two RCTs [69,70].

### 3.7. Non-Steroidal Anti-Inflammatory Drugs

Three RCTs (n = 339 patients) [60,71,72] evaluated postoperative NSAIDs (Supplemental Tables SG.1–SG.3) compared with placebo in two RCTs (n = 219 patients, one RCT each for urologic surgery and gynecologic surgery) [71,72] and usual care in one RCT (for gastrointestinal surgery) [60]. The mode of delivery was intravenous in two RCTs [60,71] and per rectum (suppository) in one RCT [72]. Mean age was 34–57 years and the proportion of females was >40%. Average BMI was 25–29 kg/m^2^. ROB was low in two RCTs [71,72] and high in one RCT [60].

### 3.8. Other Single-Study Pain Management Interventions

Fourteen RCTs met eligibility criteria, each evaluating a uniquely different intervention in a singular center/setting [73,74,75,76,77,78,79,80,81,82,83,84,85,86]. Interventions included intrathecal morphine, methadone, buprenorphine, nalbuphine, dexmedetomidine, ice, and peritoneal lavage with normal saline (full list of interventions in Supplemental Table SH.1).

### 3.9. Multimodal Opioid-Free Strategies

Seven RCTs evaluated opioid-free multimodal pain management strategies, each a unique bundle of interventions or different comparators, which hindered making any comparisons between RCTs (Supplemental Table SI.1) [87,88,89,90,91,92,93].

### 3.10. Enhanced Recovery After Surgery Protocol

Three RCTs (n = 424 patients) evaluated ERAS protocol versus usual care for laparoscopic gastrointestinal surgery (Supplemental Tables SJ.1–SJ.3) [94,95,96]. Mean age was 36–45 years and the proportion of females was between 61% and 83%. BMI was 42–45 kg/m^2^. ROB was moderate in two RCTs [95,96] and high in one RCT [94].

### 3.11. Low-Pressure Pneumoperitoneum or Active CO_2_ Removal

Thirteen RCTs (n = 2588 patients) evaluated either low-pressure pneumoperitoneum or active CO_2_ removal versus usual care (Supplemental Tables SK.1–SK.3) [97,98,99,100,101,102,103,104,105,106,107,108,109]. Interventions evaluated in this category included operating at low-pressure pneumoperitoneum or actively draining insufflation gas at the end of the procedures (e.g., with a suction drain). The evidence base included an equal share of RCTs covering gynecological and gastrointestinal surgery. The proportion of females was ≥50% in all RCTs. Mean/ median age was 36–54 years. Mean/median BMI was >30 kg/m^2^ in one RCT [105] and between 22 and 28 kg/m^2^ in the remaining RCTs. Five were low ROB [100,101,103,104,109], five moderate ROB [97,98,99,106,108], and three high ROB [102,105,107].

### 3.12. Warm and Humidified CO_2_ Insufflation

Three RCTs (n = 399 patients) evaluated warm and humid CO_2_ versus cold and dry CO_2_, for laparoscopic gastrointestinal surgery (Supplemental Tables SL.1–SL.3) [110,111,112]. Mean age was 56–69 years and the proportion of females was 30–70%. Average BMI was 23–29 kg/m^2^. ROB was low in two RCTs [111,112] and moderate in one RCT [110].

### 3.13. Complementary and Alternative Medicine Strategies

Seven RCTs each evaluated a different intervention (acupressure, virtual reality, rehabilitation, etc.) in a single center, hindering data pooling or comparisons between RCTs (Supplemental Table SM.1) [113,114,115,116,117,118,119].

## 4. Discussion

Across more than a decade of research on managing postoperative pain after laparoscopic surgery, we identified substantial heterogeneity in interventions, comparators, and outcomes, with the most trials conducted in a single center, and rated as moderate to high ROB. Evidence was highly concentrated in select interventions such as regional anesthesia and intraperitoneal LA, and many potentially valuable strategies (for example, dexamethasone or gabapentin) were evaluated only in few low-quality studies. Relatively fewer studies evaluated non-pharmacologic or multimodal strategies. Patient-reported pain and postoperative opioid consumption were the most assessed outcomes; however, the timing and reporting of these outcomes were inconsistent. Very few studies assessed longer-term outcomes such as postoperative opioid use beyond 48 h or at hospital discharge, which remains a critical evidence gap [3,120]. Adverse effects, hospital LOS, and patient satisfaction were infrequently reported, while other important secondary outcomes such as quality of life, readmissions, or quality of recovery were rarely captured.

### 4.1. Interventions with Emerging or Consistent Evidence

Regional anesthesia and the ERAS protocol appear to be promising strategies for future research in postoperative acute pain management [121,122]. Evidence from previous RCTs indicate improvements in early pain control and patient satisfaction, with minimal safety concerns, justifying the need for a rigorous comparative effectiveness review [121,122]. Intraperitoneal LA instillation also appears to have more evidence signaling reduction in early postoperative pain and opioid use, justifying the need for a rigorous comparative effectiveness review. A previous review reported a significant reduction in postoperative pain scores and reduced incidence of post-laparoscopic shoulder pain, but the authors presented differences in pain scores as standardized mean differences, did not account for minimal important difference, did not standardize the time-point of pain score measurement, had high heterogeneity in meta-analyses, and did not evaluate total opioid use, thus leaving an unmet need for a rigorous comparative effectiveness review [123]. Notably, the intervention’s simplicity, safety profile, and potential cumulative effect when combined with other modalities may render it a pragmatic choice for further research. NSAIDs remain a mainstay of multimodal analgesia per current guidelines, given the low-risk profile [124,125]. Acetaminophen showed sparse evidence in isolation, in laparoscopic surgery populations, though its inclusion in multimodal bundles remains pragmatically justified [126]. Consistent with previous systematic reviews, low-pressure pneumoperitoneum or active CO_2_ removal showed small improvements in pain at 24–48 h; however, its impact on postoperative opioid use remains unclear [127,128]. Trials of gabapentin, dexamethasone, warm and humidified insufflation gas, and complementary and alternative medicine strategies were few and of variable quality, underscoring the need for additional targeted trials before reliable conclusions can be drawn.

### 4.2. Future Directions

Following the objectives for evidence maps, we mapped the evidence on several interventions for M-PALS to inform future comparative effectiveness reviews and future research. We did not conduct formal meta-analyses or strength-of-evidence assessments, and we did not analyze the consistency or precision of estimates across RCTs for singular outcomes. This evidence map does not endorse the superiority of any intervention, which can be determined only by formal comparative effectiveness systematic reviews.

This evidence map highlights the uneven distribution of evidence across interventions and outcomes. Future research should prioritize several key areas to advance the evidence base for postoperative pain management. First, high-quality, adequately powered, and standardized RCTs are urgently needed to clarify the independent and synergistic contributions of different modalities within multimodal pain management pathways. Previous systematic reviews that attempted pooling dissimilar intervention bundles resulted in very high heterogeneity (I^2^ > 75%), underscoring the challenge in making sound comparisons between such RCTs [121,122,123,127,128]. Second, research in this area lacks standardized outcome reporting and a standardized core outcomes set which would ensure consistent measurement of pain intensity, opioid consumption, and adverse events across studies and time points, and these need to be developed and adopted in order to advance the field [129]. Alternatively, the use of existing frameworks such as the Initiative on Methods, Measurement, and Pain Assessment in Clinical Trials (IMMPACT) recommendations could improve future syntheses to help draw practice-informing conclusions [129,130]. Third, longer follow-up periods are needed to characterize patient-reported pain and opioid use beyond 24 h postoperatively. Data on long-term outcomes, especially long-term opioid use, are still lacking. Filling this evidence gap is critically important to identify pain management strategies which effectively reduce opioid use and the risk of chronic opioid use disorder [3,120]. Fourth, subgroup analyses (or dedicated RCTs) are needed to evaluate treatment effects among special subpopulations including patients with a history of chronic opioid use or psychiatric comorbidities who may be at a high risk of developing opioid dependency or substance use disorders; these populations are underrepresented in the current evidence base, hindering the generalizability of findings to such vulnerable subpopulations [3]. Future systematic reviews should also consider stratified meta-analyses to explore whether there may be increased heterogeneity owing to differences in interventions under the same category (e.g., low-pressure pneumoperitoneum or active removal of insufflation gas). Finally, transparent reporting, including adherence to standardized reporting guidelines such as the Consolidated Standards of Reporting Trials (CONSORT) extensions for perioperative research, will enhance the reproducibility, comparability, and clinical applicability of future evidence [131,132]. Standardized statistical reporting should also be promoted [133]. Some RCTs did not report measures of variance or displayed data in figures only (without numerical estimates), and some RCTs reported medians for very skewed data. These inconsistencies would pose challenges for formal meta-analyses.

### 4.3. Limitations

We limited eligibility to studies reporting both pain- and opioid-related outcomes to allow comparisons between these two sets of outcomes because one of our key goals of mapping the evidence was to inform which strategies merit further research, but this selection criterion might introduce bias. Our review focuses on M-PALS, but the choice of interventions may depend on multiple factors and not just postoperative pain, especially for non-pharmacological interventions such as low-pressure pneumoperitoneum or active removal of insufflation gas [127,128]. We show the clustering of evidence and signals of any effectiveness in the bubble plots. However, they should not be used as a substitute for a comparative effectiveness review and should not be used to claim superiority of interventions. They are only intended to help prioritize the selection of interventions for future evaluation, research, and comparative effectiveness reviews.

## 5. Conclusions

This evidence map of interventions for M-PALS reveals a fragmented evidence base. Regional anesthesia, intraperitoneal or preperitoneal LA instillation, dexamethasone, and ERAS protocols showed the most frequent signals of benefit, while adverse events were generally comparable across groups. However, study design limitations including inconsistent reporting of outcomes challenge making comparisons and constrain evidence certainty. Future research should prioritize standardized and long-term core outcomes, especially for postoperative opioid use, and conduct rigorous head-to-head comparisons to address current gaps and inform clinical practice.

## Data Availability

No new data were created or analyzed in this study.

## Supplementary Materials

The following supporting information can be downloaded at: https://www.mdpi.com/article/10.3390/jcm15082872/s1, MPALS Evidence Map.
