# Supplementary material for: Evidence Map of Pharmacologic and Non-Pharmacologic Perioperative Strategies for Managing Acute Postoperative Pain After Laparoscopic Surgery, 2012–2025: The M-PALS Collaborative
Source: J Clin Med. 2026 Apr 10;15(8):2872. doi: 10.3390/jcm15082872 (PMC13116218; doi:10.3390/jcm15082872)
Supplement: Supplementary file 1 [file jcm-15-02872-s001.zip › jcm-4169550-supplementary.pdf]

Supplementary Material

Title: Evidence Map of Pharmacologic and Non-Pharmacologic Perioperative Strategies for Managing Acute Postoperative Pain After Laparoscopic Surgery, 2012–2025: The M-PALS Collaborative

Contents

Supplementary Material: MPALS Evidence Map 1
- [SI] Methods 1
  - [A] Key Questions 1
  - [B] Search Strategy 1
  - [C] Selection Criteria 8
  - [D] Data Extraction 12
  - [E] Study Quality and Bias Assessment 12
  - [F] Data Analysis 13
  - [G] PRISMA Flow Diagram 14
- [SII] Results 15
  - [A] Regional Anesthesia 15
  - [B] Preoperative intraperitoneal or preperitoneal instillation of local anesthetics 45
  - [C] Incisional local anesthetics 59
  - [D] Gabapentinoids 67
  - [E] Intravenous dexamethasone 70
  - [F] Acetaminophen 75
  - [G] Nonsteroidal anti-inflammatory drugs (NSAIDs) 80
  - [H] Other pain management interventions 84
  - [I] Multimodal opioid-free strategies 86
  - [J] ERAS protocol 88
  - [K] Active CO2 Removal 92
  - [L] Warm humidified insufflation 104
  - [M] Complementary and alternative medicine strategies 109
- [SIII] Alphabetized List of Included Studies 112

[SI] Methods

[SIA] Key Questions

The primary objective of this evidence map is to identify, categorize, and visually summarize the existing evidence on pain management strategies used to reduce opioid consumption and improve patient-reported pain outcomes among adults undergoing laparoscopic or robotic abdominal surgery. The evidence map will encompass a broad range of interventions, including non-opioid pharmacologic therapies (across all routes of administration), regional anesthesia techniques, non-pharmacologic modalities, and multimodal or bundled approaches. A secondary objective is to explore the distribution of evidence across key patient subgroups, such as individuals with pre-existing chronic opioid use, chronic pain conditions, or comorbid psychiatric diagnoses, to highlight areas where evidence is concentrated or lacking.

[SIB] Search Strategy

A comprehensive literature search was conducted across major electronic databases, including MEDLINE, Embase, and Scopus (Elsevier). The search was restricted to English-language publications from 2012 through 2024. Initial searches were completed on January 21, 2024, and subsequently updated on January 30, 2025. A professional medical librarian, in collaboration with the research team comprising both clinical and methodological experts, developed the detailed search strategies following guidance in the Peer Review of Electronic Search Strategies (PRESS) checklist (see Appendix A). In addition, ClinicalTrials.gov was queried to identify ongoing or unpublished studies to support the assessment of potential reporting bias. Search Strategies are presented below:

Ovid MEDLINE(R)

1 exp Laparoscopy/ or Hand-Assisted Laparoscopy/ or Minimally Invasive Surgical Procedures/ or Robotic Surgical Procedures/ or Cholecystectomy, Laparoscopic/ or Nephrolithotomy, Percutaneous/

2 (laparoscop* or (("minimally invasive" or robotic*) adj2 (assisted or surg*))).mp.

3 1 or 2

4 Abdomen/ or Appendicitis/ or exp Bariatric Surgery/ or exp Hernia, Abdominal/ or exp Digestive System Neoplasms/ or exp Digestive System Surgical Procedures/ or gallbladder/ or exp gallbladder diseases/ or gastrectomy/ or gastroenterostomy/ or exp Gastroenteritis/ or exp Gynecologic Surgical Procedures/ or hepatectomy/ or Herniorrhaphy/ or exp inflammatory bowel diseases/ or exp Intestinal Diseases/ or exp Intestine, Small/ or exp Liver/ or exp Pancreas/ or pancreatectomy/ or pancreaticoduodenectomy/ or exp Stomach/ or exp Stomach Diseases/

5 (abdomen or abdomin* or adrenal* or appendectom* or appendicitis or appendix or bariatric or bowel or cholecystectom* or cholecystitis or colectom* or colonoscop* or endometri* or gall bladder? or

gallbladder? or gastrectom* or gastric or gastroenterostom* or gyn?ecologic* or hepatectom* or hernia? or herniorrhaph* or herniotom* or Hernioplast* or hysterectom* or inguinal* or intestin* or kidney? or liver or myomectomy or nephrectom* or nephrolithotom* or pancreas or pancreatectom* or pancreatic* or peritoneal or peritoneum or proctocolectom* or retroperitoneum or spleen? or splenectom* or stomach or umbilic*).mp.

6 4 or 5

7 Acute pain/ or Breakthrough pain/ or Pain/ or exp Pain Perception/ or Pain, Postoperative/ or (Postoperative Period/ and pain.mp.) or Pain, Referred/ or Visceral pain/

8 (pain* adj10 ("after surg*" or day? or discharge? or hour? or month? or post* or week?)).mp.

9 7 or 8

10 and/3,6,9

11 exp Analgesics, Opioid/ or exp Opioid-Related Disorders/ or Opiate Substitution Treatment/ or ((opiate$1 or opioid*) adj3 (disorder? or abuse or addict* or analgesic? or consumption or dependen* or discharge or dosage or dose or dosing or manage* or misuse or post* or prescrib* or prescription? or regimen? or substitut* or treat*)).mp.

12 exp analgesics/ or exp analgesics, non-narcotic/ or analgesics, short-acting/ or exp Anti-Inflammatory Agents, Non-Steroidal/ or (analgesi* or acetylsalicylic or acetaminophen or aspirin or "Cyclooxygenase 2" or COX2 or "COX-2" or dexmedetomidine or ibuprofen or Naproxen or nonopioid or non-opioid or "anti-inflammator*" or NSAIDs or "opioid sparing" or piroxicam or rescue).mp.

13 Adjuvants, Anesthesia/ or exp "anesthesia and analgesia"/ or (analges* or an?esthe* or audioanalges* or anxiolytic$1 or conscious sedation or cryoan?esthe* or deep sedation or electroacupuncture or electronarcosis or epidural or hypnoan?esthe* or hypnosedat* or neuroleptan?alges* or prean?esthe*).mp.

14 Nerve Block/ or Autonomic Nerve Block/ or Sphenopalatine Ganglion Block/ or (block? or blockade?).mp.

15 exp anesthetics/ or anesthetics, combined/ or anesthetics, local/ or exp "hypnotics and sedatives"/ or exp narcotics/ or tranquilizing agents/ or anti-anxiety agents/ or lorazepam/ or midazolam/ or (an?esthe* or anti-anxiety or bupivacaine or hypnotic? or ketamine or lorazepam or methadone or midazolam or morphine or narcotic? or paracetamol or propofol or ropivacaine or sedative? or tramadol or tranquiliz*).mp.

16 Combined Modality Therapy/ or ((drug or pain) adj5 (bundle? or combin* or modality or modalities or multimodal* or multi-modal* or regimen or therap* or treat*)).mp.

17 exp complementary therapies/ or (acupoint or acupuncture or acupressure or ((alternative or complementary or traditional) adj medicine) or biofeedback or breathing or cognitive-behavio?ral or dry

needling or guided imagery or holistic or homeopath* or hypnosis or hypnotherapy or massag* or meditation or mesotherapy or mind-body or mindfulness or moxibustion or musculoskeletal manipulations or music or osteopathic or psychotherap* or relaxation or therapeutic touch*).mp.

18 exp Electric Stimulation Therapy/ or ((electric* adj stimulat*) or TENS).mp.

19 Insufflation/ or (insufflat* adj3 (heat* or humidif* or warm*)).mp.

20 Pneumoperitoneum, Artificial/ or (pneumoperitoneum adj3 (low or pressure or reduc*)).mp.

21 Peritoneal Lavage/ or ((intraperitoneal or peritoneal) adj2 (irrigation or lavage or wash*)).mp.

22 Pain Management/ or (pain adj5 (control* or killer? or manage* or modalit* or reduc* or regimen? or relief or reliever? or treat*)).mp.

23 Patient Education as Topic/ or patient participation/ or (patient adj3 (educat* or participat*)).mp.

24 or/11-23

25 10 and 24

26 25 not (case reports/ or comment/ or editorial/ or letter/ or clinical conference/)

27 26 not ((exp infant/ or exp child/ or adolescent/ or Obstetrics/ or pregnant women/ or exp Pediatrics/) not exp adult/)

28 27 and ((randomized controlled trial or controlled clinical trial).pt. or random*.ti,ab,kf. or placebo.ab. or drug therapy.fs. or trial.ab. or groups.ab.)

29 limit 28 to (english language and yr="2012 -Current")

Cochrane Highly Sensitive Search Strategy for identifying controlled trials in Medline (Ovid). Box 3.c., Technical Supplement to Chapter 4: Searching for and Selecting Studies. Cochrane Handbook for Systematic Reviews of Interventions Version 6. Date access: January 30, 2025. https://training.cochrane.org/handbook/version-6/chapter-4-tech-suppl

Embase

1 laparoscopic surgery/ or exp laparoscopic adrenalectomy/ or laparoscopic appendectomy/ or hand assisted laparoscopy/ or laparoscopic cholecystectomy/ or laparoscopic hysterectomy/ or exp percutaneous nephrolithotomy/ or laparoscopic sleeve gastrectomy/ or exp laparoscopic gastrectomy/ or laparoscopic liver resection/ or laparoscopic myomectomy/ or exp laparoscopic nephrectomy/ or minimally invasive surgery/ or robot assisted surgery/

2 (laparoscop* or (("minimally invasive" or robotic*) adj2 (assisted or surg*))).ti,ab,hw,kw.

3 or/1-2

4 Abdomen/ or exp abdominal surgery/ or Appendicitis/ or exp Hernia, Abdominal/ or exp Digestive System Neoplasms/ or gallbladder/ or exp gallbladder diseases/ or exp Gastroenteritis/ or exp Gynecologic Surgical Procedures/ or exp inflammatory bowel diseases/ or exp Intestinal Diseases/ or exp Intestine, Small/ or exp Liver/ or exp Pancreas/ or Spleen/ or exp Stomach/ or exp Stomach Diseases/

5 (abdomen or abdomin* or adrenal* or appendectom* or appendicitis or appendix or bariatric or bowel or cholecystectom* or cholecystitis or colectom* or colonoscop* or endometri* or gall bladder? or gallbladder? or gastrectom* or gastric or gastroenterostom* or gyn?ecologic* or hepatectom* or hernia? or herniorrhaph* or herniotom* or Hernioplast* or hysterectom* or inguinal* or intestin* or kidney? or liver or myomectomy or nephrectom* or nephrolithotom* or pancreas or pancreatectom* or pancreatic* or peritoneal or peritoneum or proctocolectom* or retroperitoneum or spleen? or splenectom* or stomach or umbilic*).ti,ab,hw,kw. 6698394

6 or/4-5

7 breakthrough pain/ or pain/ or nociceptive pain/ or visceral pain/ or postoperative pain/

8 (pain* adj10 ("after surg*" or day$1 or discharge$1 or hour$1 or month$1 or post* or week$1)).ti,ab,kw.

9 or/7-8

10 and/3,6,9

11 exp opiate addiction/ or opiate substitution treatment/ or ((opiate$1 or opioid*) adj3 (disorder? or abuse or addict* or analgesic? or consumption or dependen* or discharge or dosage or dose or dosing or manage* or misuse or post* or prescrib* or prescription? or regimen? or substitut* or treat*)).ti,ab,kw.

12 analgesic agent/ or short acting analgesic agent/ or antinociceptive agent/ or antipyretic analgesic agent/ or antiinflammatory agent/ or cyclooxygenase 2 inhibitor/ or nonsteroid antiinflammatory agent/ or acetylsalicylic acid/ or dexmedetomidine/ or ibuprofen/ or naproxen/ or paracetamol/ or piroxicam/ or (analgesi* or acetylsalicylic or acetaminophen or aspirin or "Cyclooxygenase 2" or COX2 or "COX-2" or dexmedetomidine or ibuprofen or Naproxen or nonopioid or non-opioid or "anti-inflammator*" or NSAIDs or "opioid sparing" or naproxen or paracetamol or piroxicam or rescue).ti,ab,hw,kw.

13 analgesia/ or acupuncture analgesia/ or antinociception/ or anxiolytic agent/ or audioanalgesia/ or electroanalgesia/ or epidural analgesia/ or hypnotic agent/ or hypnotic sedative agent/ or interpleural analgesia/ or neuroleptanalgesia/ or patient controlled analgesia/ or postoperative analgesia/ or propofol/ or sedative agent/ or tranquilizer/ or (analges* or anti-anxiety or audioanalges* or anxiolytic$1 or conscious sedation or cryoan?esthe* or deep sedation or electroacupuncture or electronarcosis or epidural or hypnoan?esthe* or hypnosedat* or hypnotic? or lorazepam or neuroleptan?alges* or prean?esthe* or propofol or sedativ* or tranquiliz*).ti,ab,hw,kw.

14 anesthesia/ or anesthesia induction/ or exp epidural anesthesia/ or exp general anesthesia/ or exp intravenous anesthesia/ or exp local anesthesia/ or spinal anesthesia/ or anesthetic agent/ or exp inhalation anesthetic agent/ or intravenous anesthetic agent/ or local anesthetic agent/ or analgesics, opioid/ or narcotic analgesic agent/ or methadone/ or morphine/ or opiate/ or opiate agonist/ or oxycodone/ or tramadol/ or (anesthe* or fentanyl or morphine or narcotic? or opiate? or opioid? or oxycodone or tramadol).ti,ab,hw,kw.

15 nerve block/ or brachial plexus anesthesia/ or intercostal nerve block/ or lumbar plexus block/ or quadratus lumborum block/ or regional anesthesia/ or transversus abdominis plane block/ or (block? or blockade?).ti,ab.

16 combination drug therapy/ or ((drug or pain) adj5 (bundle? or combin* or modality or modalities or multimodal* or multi-modal* or regimen or therap* or treat*)).ti,ab,kw.

17 exp alternative medicine/ or exp acupuncture/ or cognitive behavioral therapy/ or guided imagery/ or hypnosis/ or massage/ or mindfulness/ or mindfulness-based cognitive therapy/ or mindfulness meditation/ or exp musculoskeletal manipulation/ or exp osteopathic medicine/ or transcutaneous electrical nerve stimulation/ or (acupoint or acupressure or acupuncture or alternative medicine or anthroposophy or biofeedback or breathing exercise? or complementary medicine or cognitive-behavioral therap* or dry needling or Electric Stimulation Therapy or electroacupuncture or guided imagery or holistic therap* or homeopath* or hypnosis or hypnotherapy or massage or meditation or mesotherapy or mind-body therap* or mindfulness or moxibustion or musculoskeletal manipulations or music therap* or traditional medicin* or osteopathic or psychotherap* or relaxation therap* or therapeutic touch* or transcutaneous electrical nerve stimulation or TENS).ti,ab,kw.

18 laparoscopic humidification system/ or laparoscopic insufflator/ or (insufflat* adj3 (heated or humidifi* or warm*)).ti,ab,kw.

19 artificial pneumoperitoneum/ or (pneumoperitoneum adj3 (low or pressure or reduced)).ti,ab,kw.

20 peritoneum lavage/ or ((intraperitoneal or peritoneal) adj2 (irrigation or lavage or wash*)).ti,ab,kw.

21 pain clinic/ or (pain adj5 (control* or killer? or manage* or modalit* or reduce* or regimen? or relief or reliever? or treat*)).ti,ab,kw.

22 patient education/ or patient participation/ or (patient educat* or patient participat*).ti,ab,kw.

23 or/11-22

24 10 and 23

25* (Randomized controlled trial/ or Controlled clinical study/ or random$.ti,ab. or randomization/ or intermethod comparison/ or placebo.ti,ab. or (compare or compared or comparison).ti. or ((evaluated or evaluate or evaluating or assessed or assess) and (compare or compared or comparing or comparison)).ab. or (open adj label).ti,ab. or ((double or single or doubly or singly) adj (blind or blinded or blindly)).ti,ab.

or double blind procedure/ or parallel group$1.ti,ab. or (crossover or cross over).ti,ab. or ((assign$ or match or matched or allocation) adj5 (alternate or group$1 or intervention$1 or patient$1 or subject$1 or participant$1)).ti,ab. or (assigned or allocated).ti,ab. or (controlled adj7 (study or design or trial)).ti,ab. or (volunteer or volunteers).ti,ab. or human experiment/ or trial.ti.) not (((random$ adj sampl$ adj7 ("cross section$" or questionnaire$1 or survey$ or database$1)).ti,ab. not (comparative study/ or controlled study/ or randomi?ed controlled.ti,ab. or randomly assigned.ti,ab.)) or (Cross-sectional study/ not (randomized controlled trial/ or controlled clinical study/ or controlled study/ or randomi?ed controlled.ti,ab. or control group$1.ti,ab.)) or (((case adj control$) and random$) not randomi?ed controlled).ti,ab. or (Systematic review not (trial or study)).ti. or (nonrandom$ not random$).ti,ab. or "Random field$".ti,ab. or (random cluster adj3 sampl$).ti,ab. or ((review.ab. and review.pt.) not trial.ti.) or ("we searched".ab. and (review.ti. or review.pt.)) or "update review".ab. or (databases adj4 searched).ab. or ((rat or rats or mouse or mice or swine or porcine or murine or sheep or lambs or pigs or piglets or rabbit or rabbits or cat or cats or dog or dogs or cattle or bovine or monkey or monkeys or trout or marmoset$1).ti. and animal experiment/) or (Animal experiment/ not (human experiment/ or human/)))

26 24 and 25

27 limit 26 to (english language and yr="2012 -Current")

28 limit 27 to (books or chapter or conference abstract or conference paper or "conference review" or editorial or letter or note or "preprint (unpublished, non-peer reviewed)" or tombstone) 1659

29 27 not 28

30 book/ or case report/ or exp conference paper/ or editorial/ or letter/ or note/

31 29 not 30

32 31 not (exp juvenile/ not exp adult/)

*Cochrane Highly Sensitive Search Strategy for identifying controlled trials in Embase. Source: Box 3.e. Technical Supplement to Chapter 4: Searching for and Selecting Studies. Cochrane Handbook for Systematic Reviews of Interventions Version 6. Date access: January 30, 2025. https://training.cochrane.org/handbook/version-6/chapter-4-tech-suppl

Scopus (Elsevier)

INDEXTERMS (laparoscop*) OR TITLE-ABS (laparoscop* W/7 abdomen OR abdomin* OR adrenalectom* OR appendectom* OR "bariatric surg*" OR "bowel resection" OR cholecystectom* OR colectom* OR colonoscop* OR "gall bladder" OR gastrectom* OR "gastric bypass" OR gastroenterostom* OR hepatectom* OR hernia* OR intestin* OR kidney* OR liver OR nephrectom* OR nephrolithotom* OR pancreas OR pancreatectom* OR pancreaticoduodenectom* OR proctocolectom*

OR retroperitoneum OR spleen OR splenectom* OR stomach) OR TITLE-ABS ("minimally invasive" W/7 abdomen OR abdomin* OR adrenalectom* OR appendectom* OR "bariatric surg*" OR "bowel resection" OR cholecystectom* OR colectom* OR colonoscop* OR "gall bladder" OR gastrectom* OR "gastric bypass" OR gastroenterostom* OR hepatectom* OR hernia? OR intestin* OR kidney? OR liver OR nephrectom* OR nephrolithotom* OR pancreas OR pancreatectom* OR pancreaticoduodenectom* OR proctocolectom* OR retroperitoneum OR spleen OR splenectom* OR stomach) AND INDEXTERMS ("postoperative pain") OR TITLE-ABS (pain W/3 postoperative) OR TITLE-ABS (opioid) AND INDEXTERMS ("pain management") OR INDEXTERMS (anesthesia) OR INDEXTERMS (analgesia) OR TITLE-ABS (analgesic* OR anesthesia OR antinociceptive OR antipyretic OR {acetylsalicylic acid} OR ibuprofen OR naproxen OR paracetamol OR {anti-inflammatory agent} OR {cyclooxygenase 2 inhibitor} OR {muscle relaxant} OR antispasmodics OR naproxen OR {non-opioid} OR {nonsteroidal anti-inflammatory drugs} OR nsaids OR paracetamol OR piroxicam OR analgesia OR antinociception OR audioanalgesia OR electroanalgesia OR epidural OR neuroleptanalgesia OR "abdominal wall block*" OR "brachial plexus block" OR "erector spinae plane block*" OR "ESP block*" OR "neuraxial block*" OR "neuromuscular block*" OR "nerve block*" OR "quadratus lumborum block*" OR "transversus abdominis plane block*" OR "TAP Block*" OR "Transversalis fascia plane block*" OR "TFP block*" OR anesthetic OR anxiolytic OR bupivacaine OR dexmedetomidine OR hypnotic OR lidocaine OR ketamine OR midazolam OR methadone OR morphine OR narcotic OR opioid OR propofol OR ropivacaine OR sedative OR tramadol OR tranquiliz* OR multimodal* OR multi-modal* OR "non-pharmacological" OR "perioperative care" OR acupoint OR acupressure OR acupuncture OR "alternative medicine" OR anthroposophy OR biofeedback OR "breathing exercise" OR "complementary medicine" OR "cognitive-behavioral therap*" OR "dry needling" OR electroacupuncture OR "guided imagery" OR "holistic therap*" OR homeopath* OR hypnosis OR hypnotherapy OR massage OR meditation OR mesotherapy OR "mind-body therap*" OR mindfulness OR moxibustion OR "musculoskeletal manipulations" OR "music therap*" OR "traditional medicin*" OR osteopathic OR psychotherap* OR "relaxation therap*" OR "therapeutic touch*" OR "patient education" OR "patient participation") OR INDEXTERMS (electroacupuncture) OR INDEXTERMS ("preanesthetic medication") OR INDEXTERMS ("complementary therapies") OR INDEXTERMS ("Electric Stimulation Therapy") OR INDEXTERMS ("laparoscopic humidification system") OR INDEXTERMS ("artificial pneumoperitoneum") OR INDEXTERMS ("peritoneum lavage") OR INDEXTERMS ("patient education") OR INDEXTERMS ("patient participation") AND PUBYEAR > 2011 AND PUBYEAR < 2025 AND NOT TITLE (child OR pediatric) AND NOT INDEXTERMS (child OR pediatric) AND TITLE-ABS-KEY ((clinic* W/1 trial*) OR (randomi* W/1 control*) OR (randomi* W/2 trial*) OR (random* W/1 assign*) OR (random* W/1 allocat*) OR (control* W/1 clinic*) OR (control* W/1 trial) OR placebo* OR (quantitat* W/1 stud*) OR (control* W/1 stud*) OR (randomi* W/1 stud*) OR (singl* W/1 blind*) OR (singl* W/1 mask*) OR (doubl* W/1 blind*) OR (doubl* W/1 mask*) OR (tripl* W/1 blind*) OR (tripl* W/1 mask*) OR (trebl* W/1 blind*) OR (trebl* W/1 mask*)) AND NOT (SRCTYPE (b) OR SRCTYPE (k) OR SRCTYPE (p) OR SRCTYPE (r) OR SRCTYPE (d) OR DOCTYPE (ab) OR DOCTYPE (bk) OR DOCTYPE (ch) OR DOCTYPE (bz) OR DOCTYPE (cp) OR DOCTYPE (cr) OR DOCTYPE (ed) OR DOCTYPE (er) OR DOCTYPE (le) OR DOCTYPE (no) OR DOCTYPE (pr) OR DOCTYPE (rp) OR DOCTYPE (re) OR DOCTYPE (sh)) AND NOT INDEX (medline) AND (LIMIT-TO (DOCTYPE , "ar")) AND (LIMIT-TO (SUBJAREA , "MEDI") OR LIMIT-TO (SUBJAREA ,

"NURS") OR LIMIT-TO (SUBJAREA , "MULT") OR LIMIT-TO (SUBJAREA , "HEAL") OR LIMIT-TO (SUBJAREA , "SOCI") OR LIMIT-TO (SUBJAREA , "PSYC"))

[SIC] Selection Criteria

We included randomized controlled trials (RCTs) enrolling adult participants (age, 18 years or greater) who underwent abdominal or pelvic laparoscopic or robotic surgery for therapeutic or diagnostic procedures. RCTs involving pediatric, obstetric, or breastfeeding populations were excluded. We included RCTs that investigated postoperative pain management interventions encompassing both pharmacologic and non-pharmacologic approaches. For inclusion, RCTs were required to report at least one pain-related outcome as well as at least one postoperative opioid use related outcome. To mitigate the risk of small study bias, each trial arm was required to include a minimum of 50 participants. The inclusion period began in 2012 for 2 main reasons. First, this timeframe coincided with the widespread adoption of multimodal and enhanced recovery protocols that substantially altered standard perioperative and analgesic practices. Second, 2012 corresponded with the height of the opioid misuse crisis in the United States, prompting a shift in pain management research toward opioid-sparing strategies and refined measurement of opioid consumption. Additional inclusion and exclusion criteria are summarized in Table S1.

Table S1. Selection Criteria Based on the PICOTS Framework

**PICOS**	**Inclusion- Eligible**	**Exclusion-not eligible**
Population	Adults undergoing abdominal laparoscopic and/or robotic surgery for therapeutic and/or diagnostic purposes<br><br>Subpopulations:<br><br>-chronic opioid use (≥90d of opioid use)<br><br>-chronic pain diagnosis<br><br>-comorbid psychiatric diagnosis.	Obstetrical & immediate (within the same hospital admission as delivery) post-partum patients, breastfeeding patients<br><br>Pediatric patients (<18 yrs old)

Interventions	-Pharmacological interventions (e.g., gabapentinoids, acetaminophen, dexamethasone, non-steroidal anti-inflammatory drugs, etc.)<br><br>-Regional anesthesia (e.g., epidural blocks)<br><br>-Non-pharmacological interventions (e.g., ice, meditation, virtual reality, use of warm humidified insufflation gas, active removal of insufflation gas, etc.)<br><br>-Bundled regimens consisting of a combination of pain management strategies listed above	- Interventions primarily for nausea<br><br>- Nutritional (diet or supplement) interventions<br><br>- Non-FDA approved drugs (off-label uses will be included)<br><br>- Other pain management techniques outside of this intervention set
Comparison	- Opioid only regimens<br><br>- Another included intervention type<br><br>- Another pain management technique outside of the included interventions above	No defined comparison or studies comparing:<br><br>- Drugs within the same drug class (e.g., celecoxib vs. ibuprofen)<br><br>- Differing dosages of the same drug/drug class<br><br>- Methods, technique, or route of delivery of the drug or procedure(s)<br><br>- Timing of regional anesthesia nerve block (e.g., before surgery vs. during surgery vs. after surgery)
Outcomes	Primary:<br><br>1. Subjective pain experience rating (e.g., visual analog scale, numeric rating scale)	Studies that do not have subjective pain experience rating and amount of opioid consumed.

	2. Cumulative amount of opioid consumed in morphine milliequivalents<br><br>Secondary:<br><br>1. In-hospital length of stay<br><br>2. Post-Anesthesia Care Unit length of stay<br><br>3.Continued opioid use at 90 days after surgery in a previously opioid naive patient<br><br>4. Functional and quality of life outcomes<br><br>5. Patient satisfaction scores<br><br>-Adverse Outcomes:<br><br>● Regional anesthesia associated- local anesthetic toxicity, hematoma, nerve or vascular damage, organ damage, dural tear, central spinal fluid leak, persistent pain, or others attributed to regional anesthesia<br><br>● Drug associated - harms reported for >5% of participants, delirium, over-sedation, GI upset/reflux/nausea, others attributed to drug<br><br>● Severe Adverse Events- death, end-organ dysfunction/failure	Other outcomes not listed
Study Designs	RCTs with intention-to-treat analysis with minimum sample size of ≥50 patients randomized per arm	● Cross-sectional<br><br>● Case-studies

		● Opinions/letters<br>● Pilot studies<br>● Non-randomized controlled trials<br>● Cohort studies<br>● RCTs without both opioid intake and a pain metric as an outcome
Study Size	≥50 participants per study arm (≥30 participants per study arm for CAM studies)	
Intervention Timing/Setting	Perioperative period: day of surgery to 24 hours postoperative	Interventions conducted in a clinic to treat chronic pain and not to prepare for surgery
Outcome Timing	Acute: immediate postoperative hour 24 hours – discharge or 7 days<br>Intermediate (discharge – 90 days)<br>Long term (>90 days)	
Geographic location	Include any/all United States or international settings	
Publication status	Articles published in peer-reviewed journals	Articles that have not been peer reviewed and are not published in peer-reviewed journals (e.g., unpublished data, manuscripts, pre-prints, reports, abstracts, conference proceedings)

Abbreviations: ED= emergency department; RCT= randomized controlled trial; SUD = substance use disorder

Database search results were imported into PICO Portal™ (www.picoportal.net), a machine-learning enabled cloud-based platform used to prioritize citations most relevant to the evidence map. Two reviewers independently screened titles and abstracts until 90% recall of eligible studies was achieved, after which a single reviewer continued screening to reach a 95% recall threshold. For articles deemed ineligible by AI, we conducted a random check of 200 titles and abstracts to verify 100% accuracy in this set as a quality check. Full-text assessments were conducted independently by 2 reviewers using the same system, with disagreements resolved through discussion and team consensus.

[SID] Data Extraction

Data extraction was performed in accordance with the *Template for Intervention Description and Replication* (TIDieR) checklist. Extracted variables included study-level metadata, study design characteristics, sample size, patient demographics (e.g., age, sex, body mass index, American Society of Anesthesiologists physical status), and information on interventions, comparators, and outcomes as defined by the PICOTS framework.

Findings tables were generated to summarize these data and synthesize results by intervention–comparator pair. For opioid consumption, cumulative use was recorded at 24, 48, and beyond 48 hours postoperatively. When studies did not specify the observation window for total opioid use, the longest reported period for pain outcomes was applied. Secondary outcomes included hospital length of stay, quality of life, patient satisfaction, 30-day readmission rates, and pain-related parameters such as trajectory, interference, rebound pain, and adverse events/ harms.

[SIE] Study Quality and Bias Assessment

Risk of bias was assessed at the study-level for each eligible RCT using the *Cochrane Risk of Bias 2.0 Tool*[12] in five key domains: bias arising from the randomization process, deviations from intended interventions, missing outcome data, outcome measurement, and selective reporting. An initial assessment was conducted by dual independent review and any disagreements were resolved through team discussion and consensus. Each RCT was assigned an overall risk-of-bias rating as being low, moderate, or high, based on domain-level judgments and the reviewers' confidence in the credibility of the findings despite potential methodological limitations. Detailed assessments at both the overall and domain levels are provided in the appendix. To support this evaluation, ClinicalTrials.gov was also reviewed to compare study protocols and registrations with published reports, identifying any discrepancies between planned and implemented methods.

[SIF] Data Analysis

Given the objectives of this evidence map, data synthesis focused on describing and visually summarizing the scope, distribution, and characteristics of the existing evidence rather than conducting meta-analyses. Data from included randomized controlled trials (RCTs) were organized by intervention, comparator, and outcome domain using Microsoft Excel (Microsoft Corp), R version 4.3.0 (R Foundation for Statistical Computing), and Tableau (Salesforce Inc) for visualization.

Study frequencies and sample size distributions were summarized across key analytic dimensions, including intervention category (non-opioid pharmacologic, regional anesthesia, non-pharmacologic, and multimodal regimens), surgical setting (laparoscopic vs robotic), and patient subgroups (e.g., chronic opioid use, chronic pain diagnosis, psychiatric comorbidities). Outcomes were grouped into clinically relevant categories using the *Core Outcome Measures in Effectiveness Trials* (COMET) framework where applicable.

Heat maps were developed to display the number of RCTs reporting a given outcome for each intervention–comparator pair, presented in three panels:

1. **Patient-reported pain outcomes**, including pain intensity at 24 hours, 48 hours, or other postoperative time points.

2. **Postoperative opioid use outcomes**, including cumulative opioid consumption at 24 hours, 48 hours, at discharge, or other intervals, as well as opioid rescue outcomes (e.g., number of patients requiring rescue doses, time to first rescue dose, and number of rescue attempts on patient-controlled analgesia).

3. **Adverse effect outcomes**, encompassing any reported treatment-related harms.

Additionally, bubble plots were created to map the overall signal for effectiveness and harms among strategies evaluated in two or more RCTs. Separate plots were generated for: (1) any patient-reported pain outcome, (2) any postoperative opioid use outcome, and (3) any adverse effect. In these visualizations, color coding denoted the overall risk-of-bias assessment for each RCT, while bubble size represented the relative number of participants randomized in each trial.

Evidence gaps were identified where limited or no RCTs addressed specific intervention–outcome pairs, subpopulations, or long-term outcomes. All analyses were performed according to procedures specified in the registered protocol (PROSPERO CRD42024569937).

[SIG] PRISMA Flow Diagram

Figure S1. PRISMA Flow Diagram for Study Selection

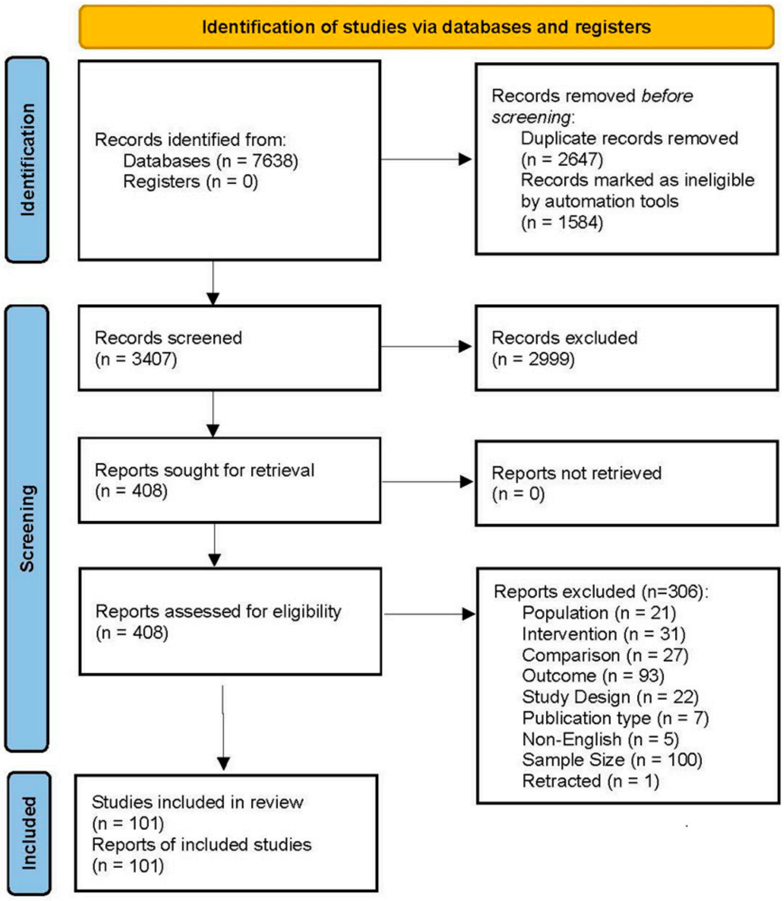

[SII] Results

[SIIA] Regional Anesthesia

Supplemental table SA.1 Regional Anesthesia vs Standard Care: Descriptive Characteristics of Studies

**Author, Year**<br>**Country**<br>**Study Design**<br>**Ambulatory or Inpatient**<br>**Risk of bias**	**Population**<br>**n randomized**<br>**Mean age (SD)**<br>**Sex (% female)**<br>**Race/ Ethnicity**<br>**Mean BMI (SD)**	**Intervention**	**Comparator**	**Outcomes of interest reported**
Chen, 2024<br>China<br>RCT<br>Inpatient<br>High	Laparoscopic Umbilical Hernia Repair with Intraperitoneal Onlay Mesh<br>Excluded neurological or psychiatric disorders<br>n=151<br>Age:<br>I: 50.10 (15.595)<br>C: 53.10 (16.522)<br>43.7% female<br>Race/ethnicity NR<br>BMI:<br>I: 27.20 (6.630)<br>C: 26.33 (6.210)	Ultrasound-Guided Rectus Sheath Block	No block	Pain scores (1, 6, 12, 24h)<br>PONV<br>Intervention harms
Terranova, 2024<br>Italy<br>RCT<br>Inpatient<br>High	Minor Gynecologic Laparoscopic Surgery<br>Excluded neurological or cognitive dysfunction.<br>n=104<br>Age:<br>I: 44.86 (12.67)<br>C: 44.18 (14.16)	TAP/RS Block	No block	Pain scores (6, 12, 18, 24, 36, 48, 72h)<br>Total opioid (48h)<br>Length of stay<br>Patient satisfaction<br>PONV

Author, Year<br>Country<br>Study Design<br>Ambulatory or Inpatient<br>Risk of bias	Population<br>n randomized<br>Mean age (SD)<br>Sex (% female)<br>Race/ Ethnicity<br>Mean BMI (SD)	Intervention	Comparator	Outcomes of interest reported
	100% female<br>Race/ethnicity NR<br>BMI:<br>I: 23.05 (3.42)<br>C: 22.37(2.64)			
Yin, 2024<br>China<br>RCT<br>Inpatient<br>Some concerns	Robot-assisted laparoscopic Nephrectomy<br>n=100<br>Age:<br>I: 54.78 (8.97)<br>C: 55.48 (8.77)<br>49% female<br>Race/ethnicity NR<br>BMI:<br>I: 23.50 (2.73)<br>C: 23.70 (1.98)	Thoracolumbar paravertebral block	No block	Pain scores (2,12, 24, 48h)<br>Total opioid (24, 48h)<br>Length of stay<br>PONV<br>PO complications
Frost, 2023<br>USA<br>RCT<br>Mixed<br>High	Laparoscopic Gyn surgery patients<br>Excluded those actively taking opioids; inclusion of chronic pain diagnoses and psychiatric diagnoses NR<br>n=215<br>Age:<br>I1: 43.9 (6.5) y<br>C1: 43.6 (7.5) y<br>C2: 44.3 (6.6) y<br>100% female<br>Race/ ethnicity excluded from analysis	Uterosacral bupivacaine administration	C1: saline<br>C2: no administration	Pain score (other timepoints)<br>Total postoperative opioid consumption (24 hr, 7 days)<br>Postoperative complications<br>Discharge prescription amount/ refills

Author, Year<br>Country<br>Study Design<br>Ambulatory or Inpatient<br>Risk of bias	Population<br>n randomized<br>Mean age (SD)<br>Sex (% female)<br>Race/ Ethnicity<br>Mean BMI (SD)	Intervention	Comparator	Outcomes of interest reported
	BMI:<br>I: 22% Normal BMI<br>C1: 17% Normal BMI<br>C2: 12% Normal BMI			
Dai, 2022<br>China<br>RCT<br>Inpatient<br>Low	Laparoscopic GI surgery patients<br>Excluded those with chronic pain, chronic opioid use, and psychiatric diagnoses<br>n= 160<br>Age:<br>I: 57.2 (14.8) y<br>C: 55.3 (14.0) y<br>69.4% female<br>Race/ Ethnicity: 98.1% Han; 1.9% Other<br>BMI: I: 24.7 (4.0) kg/m^2<br>C:23.9 (2.7) kg/m^2	TAP Block with PCIA pump	Only PCIA pump	Pain scores (24 hr, 48 hr)<br>Total postoperative opioid consumption (48 hr)<br>Hospital length of stay<br>Intervention associated harms<br>Patient satisfaction
Pedrazzani, 2021<br>Italy, Korea<br>RCT<br>Inpatient<br>Low	Laparoscopic GI surgery patients<br>Excluded those with chronic opioid use and those with psychiatric diagnoses; inclusion of chronic pain NR<br>n=108<br>Age:<br>I: 65.4 (9.8) y<br>C: 64.9 (10.4) y	Wound infiltration with Ropivacaine+TAP block Ropivacaine	Wound infiltration Ropivacaine	Pain scores (24 hr, 48 hr, 72 hr)<br>Hospital length of stay<br>Intervention associated harms<br>Postoperative complications<br>Rescue opioid metrics

**Author, Year<br>Country<br>Study Design<br>Ambulatory or Inpatient<br>Risk of bias**	**Population<br>n randomized<br>Mean age (SD)<br>Sex (% female)<br>Race/ Ethnicity<br>Mean BMI (SD)**	**Intervention**	**Comparator**	**Outcomes of interest reported**
	42% female<br>Race/ ethnicity NR<br>BMI: Median (IQR)<br>I: 24.5 (22.6–27.0) kg/m^2<br>C: 23.9 (22.6–26.2) kg/m^2			
Ruiz-Tovar, 2020<br>Spain<br>RCT<br>Inpatient<br>Low	Laparoscopic GI surgery patients<br>Inclusion of chronic opioid use, chronic pain, and psychiatric diagnoses NR<br>n=140<br>Age:<br>I: 43.1 (10.6) y<br>C: 43.9 (10.2) y<br>71% female<br>BMI:<br>I: 42.4 (3.2) kg/m^2<br>C: 42.6 (3.6) kg/m^2	TAP block Bupivacaine	No block	Pain scores (24 hr)<br>Hospital length of stay<br>Intervention associated harms<br>Postoperative complications<br>Opioid rescue metrics
Wong, 2020<br>USA<br>RCT<br>Inpatient<br>Low	Laparoscopic GI surgery patients<br>Excluded those with recent history of opioid use, chronic pain, and those with psychiatric diagnoses<br>n=219<br>Age:<br>I1:42.1 (9.8) y<br>I2: 39.4 (10.9) y<br>C: 40.4 (11.0) y<br>I1: 80.0% female	I1: TAP block Liposomal Bupivacaine<br>I2: TAP block Regular Bupivacaine	No block	Pain scores (48 hr)<br>Hospital length of stay<br>Intervention associated harms<br>Opioid rescue metrics

Author, Year<br>Country<br>Study Design<br>Ambulatory or Inpatient<br>Risk of bias	Population<br>n randomized<br>Mean age (SD)<br>Sex (% female)<br>Race/ Ethnicity<br>Mean BMI (SD)	Intervention	Comparator	Outcomes of interest reported
	I2: 78.1% female<br>C: 90.1% female<br>Race/ ethnicity:<br>I1/I2/C - %<br>Black -33.3/ 37.0 /33.8<br>Caucasian 21.3 /5.5/ 9.9<br>Hispanic 45.3/ 56.2/ 53.5<br>Other 0 /1.4 /2.8<br>BMI:<br>I1:44.5 (7.6) kg/m^2<br>I2: 44.8 (5.5) kg/m^2<br>C: 44.2 (5.5) kg/m^2			
Kinjo, 2019<br>Japan<br>RCT<br>Inpatient<br>Low	Laparoscopic Gyn surgery patient<br>Inclusion of those with chronic pain, chronic opioid use, and those with psychiatric diagnoses NR<br>n=210<br>Age:<br>I:39.2 (10.3) y<br>C: 41.6 (13.3) y<br>100% female<br>Race/ ethnicity NR<br>BMI:<br>I:21.6 (3.2) kg/m^2<br>C: 21.8 (3.0) kg/m^2	Rectus sheath field block - ropivacaine	No block	Pain scores (24 hr, 48 hr, 72 hr)<br>Total postoperative opioid consumption (timing unspecified)<br>Postoperative complications

Abbreviations: BMI= body mass index; C= control/comparator; d/c= discharge; GI= gastrointestinal; Gyn=gynecological; hr= hour(s); I= intervention; kg= kilogram; m=meter; n= number of participants; NR= not reported; PCIA= patient controlled intravenous analgesia; RCT= randomized controlled trial; SD= standard deviation; TAP= transversus abdominis plane; USA= United States of America; y= year(s)

Supplemental table SA.2 Regional Anesthesia vs Standard Care: Risk of Bias

Author, Year	Bias from randomization process	Bias from deviation from intended interventions (assignment)	Bias due to missing outcome data	Bias in measurement of outcome	Bias in selection of reported results	Overall risk of bias (low, some concerns, high)
Chen, 2024	Some concerns	High	Low	Low	High	High
Terranova, 2024	Low	Some concerns	High	Low	Low	High
Yin, 2024	Low	Some concerns	Low	Low	Low	Some concerns
Frost, 2023	High	Low	Low	Low	High	High
Dai, 2022	Low	Low	Low	Low	Low	Low
Pedrazzani, 2021	Low	Low	Low	Low	Low	Low
Ruiz-Tovar, 2020	Low	Low	Low	Low	Low	Low
Wong, 2020	Low	Low	Low	Low	Low	Low
Kinjo, 2019	Low	Low	Low	Low	Low	Low

Supplemental table SA.3 Regional Anesthesia vs Standard Care: Summary of Findings

**Quality assessment**	**Results**
**Number of studies**<br>**n analyzed**	
**24-hour: Pain scores**	
7 RCTs<br>n=962	Pedrazzani, 2021 reported a mean pain score of 3.80 (SD 2.40) in the experimental group and 3.60 (SD 2.30) in the control group, with a mean difference of 0.20 (95% CI −0.71 to 1.11).

Quality assessment<br>Number of studies<br>n analyzed	Results
	Ruiz-Tovar, 2020 reported mean scores of 1.66 (SD 1.14) versus 3.54 (SD 1.27), with a mean difference of −1.88 (95% CI −2.28 to −1.48).<br>Chen, 2025 reported mean scores of 1.77 (SD 0.70) versus 3.25 (SD 0.73), with a mean difference of −1.48 (95% CI −1.72 to −1.24).<br>Terranova, 2024 reported mean scores of 1.08 (SD 1.80) versus 4.75 (SD 1.69), with a mean difference of −3.67 (95% CI −4.34 to −3.00).<br>Kinjo, 2019 reported mean pain score on a 0-5 scale and found no significant difference.<br>Dai, 2022 reported pain as proportions in a scale from 0 to 3 and found a significant difference in the proportion of participants with high pain scores favoring the intervention.<br>Yin, 2024 reported median pain scores in figures and found a significant difference favoring intervention.
**48-hour pain scores**	
6 RCT<br>n=862	Pedrazzani, 2021 reported a mean score of 2.00 (SD 1.90) in the experimental group and 2.00 (SD 2.40) in the control group, with a mean difference of 0.00 (95% CI −0.84 to 0.84).<br>Wong, 2020 reported mean scores of 4.70 (SD 1.70) versus 4.70 (SD 1.50), with a mean difference of 0.00 (95% CI −0.52 to 0.52).<br>Terranova, 2024 reported mean scores of 0.34 (SD 1.21) versus 3.21 (SD 2.22), with a mean difference of −2.97 (95% CI −3.66 to −2.28).<br>Kinjo, 2019 reported mean pain score on a 0-5 scale and found no significant difference.<br>Dai, 2022 reported pain as proportions in a scale from 0 to 3 and found no significant difference.<br>Yin, 2024 reported median pain scores in figures and found a significant difference favoring intervention.

**Quality assessment**<br>**Number of studies**<br>**n analyzed**	**Results**
**72 hour pain scores**	
3 RCTs<br>n=458	Two RCTs reported mean pain scores using 0-10 scale, one found a significant difference favoring intervention (Terranova, 2024- data reported only in Figures without numbers); Pedrazzani, 2021 reported no significant difference<br><br>Kinjo, 2019 reported a mean pain score on a 0-5 scale and found no significant difference.
**24 hour: Total postoperative opioid consumption**	
2 RCT<br>n=220	Frost, 2023 reported medians and found no significant difference between groups.<br><br>Yin, 2024 reported means and found a significant difference favoring intervention.<br>Mean(SD)<br>I: 26.1(5.1)<br>C: 41.37 (8.22)<br>$p<0.001$
**48 hour: Total postoperative opioid consumption**	
3 RCT<br>n = 306	Pedrazzani, 2021 reported a mean value of 364.90 (SD 9.00) OME in the experimental group and 369.50 (SD 9.50) OME in the control group, with a mean difference of −4.60 (95% CI −8.19 to −1.01) OME.<br>Terranova, 2024 reported mean values of 0.00 (SD 0.51) versus 0.24 (SD 0.75) OME, with a mean difference of −0.24 (95% CI −0.49 to 0.01) OME.<br>Yin, 2024 reported mean values of 65.49 (SD 12.84) versus 81.81 (SD 14.28) OME, with a mean difference of −16.32 (95% CI −21.64 to −11.00) OME.

**Quality assessment**	**Results**
**Number of studies**<br>**n analyzed**	
**Opioid Rescue Analgesia (for those that don't have cumulative total opioid consumption)**	
3 RCT<br>n = 386	Two RCTs reported % of patients requiring additional opioids. One study found a significant difference favoring the intervention (Ruiz-tovar, 2020) and the other found no significant difference (Wong, 2020).<br><br>Two studies reported the amount of opioids (not cumulative) consumed. One reported Defined Daily Dose of tramadol and found a significant difference favoring the intervention on POD 3, but no significant difference on POD 1 (Pedrazzani, 2021). Another study reported total PCA in fentanyl equivalents at 48 hours and found no significant difference between arms (Wong, 2020).
**Patient satisfaction**	
2 RCTs<br>n = 299	Dai, 2022 reported satisfaction proportion in a 0-4 scale and found significant differences favoring intervention.<br><br>Terranova, 2024 reported mean satisfaction score (0-10 scale) and found a significant difference favoring intervention.
**Discharge prescription amount/ refills**	
1 RCT<br>n=120	Frost, 2023 reported frequency of opioid refill requests and did not statistically compare frequency between arms.
**Hospital length of stay (LOS; days)**	
6 RCT<br>n = 752	Wong, 2020 reported a mean value of 1.50 (SD 0.80) d in the experimental group and 1.40 (SD 0.50) d in the control group, with a mean difference of 0.10 (95% CI −0.12 to 0.32) d. Pedrazzani, 2021 reported mean values of 6.35 (SD 2.29) d versus 6.18 (SD 3.43) d, with a mean difference of 0.17 (95% CI −0.96 to 1.30) d.

Quality assessment<br>Number of studies<br>n analyzed	Results
	Terranova, 2024 reported mean values of 1.92 (SD 0.41) d versus 2.71 (SD 0.72) d, with a mean difference of −0.79 (95% CI −1.02 to −0.56) d.<br><br>Three RCTs reported median LOS, Ruiz-Tovar, 2020 found no differences, Dai 2022 favored the control group, and Yin 2024 favored the intervention group.
**Intervention associated harms**	
7 RCT<br>n = 785	Chen, 2024 reported no cases of peritoneal oozing or local anesthetic toxicity; postoperative nausea and vomiting occurred in 17.6% of the intervention group and 26.7% of the control group (p = 0.103).<br>Terranova, 2024 reported no complications related to injection, intravenous local anesthetic toxicity, or anaphylaxis; postoperative nausea and vomiting occurred in 9% of the intervention group and 25.5% of the control group (p = 0.09).<br>Yin, 2024 reported no procedure-related complications such as hematoma, infection, pneumothorax, or nerve injury; dizziness occurred in 6% of the intervention group and 8% of controls (p = 0.695), nausea in 6% versus 18% (p = 0.65), and emesis in 4% versus 12% (p = 0.140).<br>Dai, 2022 reported similar rates of postoperative nausea and vomiting in the intervention and control groups (18.8% each; p = 1.000); unplugging of the analgesic pump due to adverse reactions occurred in 7.5% of the intervention group and 2.5% of controls (p = 0.276), and other adverse events including hypotension, bradycardia, respiratory depression, itching, infection, and combination therapy use did not differ significantly between groups.<br>Pedrazzani, 2021 reported postoperative nausea and vomiting in 34% of the intervention group and 25% of the control group (p = 0.39), with no other intervention-related adverse events reported.

Quality assessment<br>Number of studies<br>n analyzed	Results
	Ruiz-Tovar, 2020 reported postoperative nausea and vomiting in 1.4% of the intervention group and 11.4% of the control group (odds ratio 4.27; 95% CI 1.8–10.6; p = 0.039), with no other complications reported.<br>Wong, 2020 reported nausea in 57.8% of the intervention group and 46.5% of the control group (p > 0.05), and stated that no adverse effects attributable to the intervention were observed.

Abbreviations: C= comparator/control; CI= confidence interval; I= intervention; IQR= interquartile range; LOS= length of stay; MA= meta-analysis; mm=millimeters; n= number of participants; NR= not reported; p= p-value; PCA= patient controlled analgesia; POD= postoperative day; PONV= postoperative nausea and vomiting; RCT= randomized controlled trials; SD= standard deviation; SE= standard error; SMD = standard mean difference; SOE= strength of evidence; VAS= visual analogue scale

Supplemental table SA.4 Regional Anesthesia vs Sham: Descriptive Characteristics of Studies

**Author, Year<br>Country<br>Study Design<br>Ambulatory or In patient<br>Risk of bias**	**Population<br>n randomized<br>Mean age (SD)<br>Sex (% female)<br>Race/ Ethnicity<br>Mean BMI (SD)**	**Intervention**	**Comparator**	**Outcomes of interest reported**
Cataldo, 2024	Laparoscopic Sleeve Gastrectomy patients<br>Excluded patients with a chronic pain diagnosis and ongoing opioid therapy<br>n=110	TAP block	Sham	Pain scores at 24h<br>Morphine 24h<br>Length of hospital stay<br>Surgical complications<br>PONV

Author, Year<br>Country<br>Study Design<br>Ambulatory or In patient<br>Risk of bias	Population<br>n randomized<br>Mean age (SD)<br>Sex (% female)<br>Race/ Ethnicity<br>Mean BMI (SD)	Intervention	Comparator	Outcomes of interest reported
	Age Median (IQR)<br>I: 43.5 (35–48)<br>C: 43.0 (35–52)<br>Female<br>I: 41 (75.9%)<br>C: 42 (75.0%)<br>Race/ ethnicity excluded from analysis<br>BMI Median (IQR)<br>I: 42.0 (39.7–45.5)<br>C: 42.9 (38.9–45.8)			
Gungorduk, 2024	Laparoscopic Gyn surgery patients<br>Excluded patients with regular narcotic use<br>n = 152<br>Age mean(SD)<br>I: 56.3 (9.3)<br>C: 53.9 (10.0)<br>Female 100%<br>Race/ ethnicity excluded from analysis<br>BMI<br>I: 27.7 ± 3.3<br>C: 28.6 ± 3.1	Paracervical injection	Sham	Pain scores at 24 h<br>Length of hospital stay<br>QoR-40<br>Postoperative complications<br>Total additional analgesic
Salmonsen, 2024	Patients undergoing minimally invasive colon surgery<br>n = 360	I1: US- TAP block<br>I2: LA-TAP block	Sham	Pain scores POD1<br>Total opioid 24h<br>QoR-15<br>Length of hospital stay

Author, Year<br>Country<br>Study Design<br>Ambulatory or In patient<br>Risk of bias	Population<br>n randomized<br>Mean age (SD)<br>Sex (% female)<br>Race/ Ethnicity<br>Mean BMI (SD)	Intervention	Comparator	Outcomes of interest reported
	Age Median (IQR)<br>I1: 73.8 (63.7–78.5)<br>I2: 72.7 (64.5–77.9)<br>C: 70.5 (61.7–77.9)<br>Female n(%)<br>I1: 47 (37.0)<br>I2: 63 (49.6)<br>C: 47 (54.7)<br>Race/ ethnicity excluded from analysis<br>BMI Median(1QR)<br>I1: 25.9 (23.8–28.7)<br>I2:25.9 (23.4–29.3)<br>C: 26.1 (23.0 to 29.2)			Postoperative complications
Frost, 2023<br>USA<br>RCT<br>Mixed<br>High	Laparoscopic Gyn surgery patients<br>Excluded those actively taking opioids; inclusion of chronic pain diagnoses and psychiatric diagnoses NR<br>n=215<br>Age:<br>I1: 43.9 (6.5) y<br>C1: 43.6 (7.5) y<br>C2: 44.3 (6.6) y<br>100% female<br>Race/ ethnicity excluded from analysis<br>BMI:<br>I: 22% Normal BMI	Uterosacral ligament injection	C1: Sham block<br>C2: no block	Pain scores (on discharge)<br>Total postoperative opioid consumption (24 hr, 7 days)<br>Discharge prescription amount/ refills<br>Postoperative complications

Author, Year<br>Country<br>Study Design<br>Ambulatory or In patient<br>Risk of bias	Population<br>n randomized<br>Mean age (SD)<br>Sex (% female)<br>Race/ Ethnicity<br>Mean BMI (SD)	Intervention	Comparator	Outcomes of interest reported
	C1: 17% Normal BMI<br>C2: 12% Normal BMI			
Lu, 2023<br>China<br>RCT<br>Inpatient<br>Some concerns	Patients undergoing laparoscopic cholecystectomy<br>n= 197<br>Age:<br>I:49.50 (12.31)<br>C:47.95 (11.99)<br>Female:<br>C:71.29%<br>I:58.33%<br>Race/ ethnicity excluded from analysis<br>BMI:<br>I: 24.63 (2.90)<br>C: 24.17 (2.96)	US guided ESPB	Sham	Pain scores (30 min, 1, 2, 6, 12, 24, 36, 48h)<br><br>Patient satisfaction
Nazar, 2023<br>India<br>RCT<br>Inpatient<br>High	Laparoscopic Gyn surgery patients<br>Inclusion of those with chronic pain, chronic opioid use, and those with psychiatric diagnoses NR<br>n=138<br>Age:<br>I: 43.5 (7.9) y<br>C: 43.5 (4.4) y<br>100% female<br>Race/ethnicity NR<br>BMI NR	Quadratus lumborum block	Sham	Pain scores (24 hr)<br>Total postoperative opioid consumption (24 hr)<br>Intervention associated harms

Author, Year<br>Country<br>Study Design<br>Ambulatory or In patient<br>Risk of bias	Population<br>n randomized<br>Mean age (SD)<br>Sex (% female)<br>Race/ Ethnicity<br>Mean BMI (SD)	Intervention	Comparator	Outcomes of interest reported
Xue, 2022<br>China<br>RCT<br>Inpatient<br>Low	Laparoscopic GI surgery patients<br>Excluded those with chronic pain, chronic opioid use, and psychiatric diagnoses.<br>n=234<br>Age:<br>I1: 30.4 (7.5) y<br>I2:32.7 (6.9) y<br>C: 32.4 (7.3) y<br>75.1% female<br>Race/ ethnicity NR<br>BMI:<br>I1:43.3 (7.5) kg/m^2<br>I2: 41.8 (5.2) kg/m^2<br>C:42.2 (6.1) kg/m^2	I1: TAP Block<br>I2: Quadratus lumborum block	Sham	Pain scores (24 hr, 48 hr, discharge)<br>Hospital length of stay<br>Intervention associated harms<br>Postoperative complications<br>Rescue opioid metrics
Li, 2019<br>China<br>RCT<br>Inpatient<br>Low	Laparoscopic Urology surgery patients<br>Excluded those with chronic opioid use; inclusion of those with chronic pain and/or psychiatric diagnoses NR<br>n=104<br>Age:<br>I: 51.9 (10.3) y<br>C: 51.1 (11.1) y<br>38.8% female<br>Race/ ethnicity NR	TAP block	Sham	Pain scores (24 hr)<br>Total postoperative opioid consumption (24 hr)<br>Hospital length of Stay<br>Intervention associated harms<br>Postoperative complications

Author, Year<br>Country<br>Study Design<br>Ambulatory or In patient<br>Risk of bias	Population<br>n randomized<br>Mean age (SD)<br>Sex (% female)<br>Race/ Ethnicity<br>Mean BMI (SD)	Intervention	Comparator	Outcomes of interest reported
	BMI:<br>I: 24.8 (3.8) kg/m^2<br>C: 24.8 (3.3) kg/m^2			
Tülübaş, 2019<br>Turkey<br>RCT<br>Inpatient<br>Some concerns	Laparoscopic GI surgery patients<br>Excluded those with "drug abuse"; inclusion of those with chronic pain or psychiatric diagnoses NR<br>n=165<br>Age:<br>I: 37.97 (10.61) y<br>C: 37.88 (10.14) y<br>61.8% female<br>Race/ethnicity NR<br>BMI:<br>I: 48.03 (6.77) kg/m^2<br>C:50.96 (8.73) kg/m^2	TAP block	Sham	Pain scores (24 hr)<br>Total postoperative opioid consumption (24 hr)<br>Intervention associated harms<br>Postoperative complications
Barr, 2018<br>USA<br>RCT<br>Mixed<br>Low	Laparoscopic Gyn surgery patients<br>Inclusion of those with chronic pain, chronic opioid use, and those with psychiatric diagnoses NR<br>n=132<br>Age:<br>I: 48.4 (9.2) y<br>C: 47.8 (9.3) y<br>100% female<br>Race/Ethnicity - n (%)	Paracervical injection	Sham	Pain scores (24 hr, 48 hr)<br>Postoperative complications<br>Rescue opioid metrics

**Author, Year**<br>**Country**<br>**Study Design**<br>**Ambulatory or In patient**<br>**Risk of bias**	**Population**<br>**n randomized**<br>**Mean age (SD)**<br>**Sex (% female)**<br>**Race/ Ethnicity**<br>**Mean BMI (SD)**	**Intervention**	**Comparator**	**Outcomes of interest reported**
	White I: 36 (54) C:28 (44)<br>Black I: 12 (18) C:20 (32)<br>Hispanic I:13 (19) C:10 (16)<br>Asian I:4 (6) C:3 (5)<br>Other I: 2 (3) C:2 (4)<br>BMI:<br>I:27.2 (5.7) kg/m^2<br>C: 29.1 (6.9) kg/m^2			
Kawahara, 2015<br>Japan<br>RCT<br>Inpatient<br>Low	Laparoscopic Gyn surgery patients<br>Inclusion of those with chronic pain, chronic opioid use, and those with psychiatric diagnoses NR<br>n=119<br>Age, median (range):<br>I: 41 (24-70) y<br>C: 38 (24-53) y<br>100% female<br>Race/ ethnicity NR<br>BMI NR	TAP block	Sham	Pain scores (24 hr)<br>Total postoperative opioid consumption (12-24 hr)<br>Intervention associated harms
Calle, 2014<br>Colombia<br>RCT<br>Ambulatory<br>Low	Laparoscopic Gyn surgery patients<br>Inclusion of those with chronic pain, chronic opioid use, and those with psychiatric diagnoses NR<br>n=197<br>Age: Median (IQ [*sic*])	TAP block	Sham	Pain scores (24 hr, 48 hr, 72 hr)<br>Intervention associated harms<br>Postoperative complications<br>Rescue opioid metrics

Author, Year<br>Country<br>Study Design<br>Ambulatory or In patient<br>Risk of bias	Population<br>n randomized<br>Mean age (SD)<br>Sex (% female)<br>Race/ Ethnicity<br>Mean BMI (SD)	Intervention	Comparator	Outcomes of interest reported
	I: 44 (7) y<br>C: 43 (7) y<br>100% female<br>Race/ ethnicity NR<br>BMI:<br>I: 24.68 (4.4) kg/m^2<br>C: 25.83 (3.76) kg/m^2			
Sinha, 2013<br>India<br>RCT<br>Inpatient<br>Low	Laparoscopic GI surgery patients<br>Inclusion of those with chronic pain, chronic opioid use, and those with psychiatric diagnoses NR<br>n=100<br>Age:<br>I: 39.9 (13.3) y<br>C: 39.1 (10.6) y<br>% female NR<br>Race/ ethnicity NR<br>BMI<br>I: 48.1 (6.3) kg/m^2<br>C: 45.6 (6.6) kg/m^2	TAP block	Sham	Pain scores (24 hr)<br>Total postoperative opioid consumption (24 hr)<br>Patient satisfaction scores<br>Intervention associated harms<br>Postoperative complications

Abbreviations: Abbreviations: BMI= body mass index; C= control/comparator; d/c= discharge; GI= gastrointestinal; Gyn=gynecological; hr= hour(s); I= intervention; kg= kilogram; m=meter; n= number of participants; NR= not reported; PCIA= patient controlled intravenous analgesia; RCT= randomized controlled trial; SD= standard deviation; TAP= transversus abdominis plane; USA= United States of America; y= year(s)

Supplemental table SA.5 Regional Anesthesia vs Sham: Risk of Bias

Author, Year	Bias from randomization process	Bias from deviation from intended interventions (assignment)	Bias due to missing outcome data	Bias in measurement of outcome	Bias in selection of reported results	Overall risk of bias (low, some concerns, high)
Cataldo, 2024	Low	Some concerns	Low	Low	Low	Some concerns
Gungorduk, 2024	Low	Some concerns	Low	Low	Low	Some concerns
Salmonsen, 2024	Low	Low	Low	Low	Low	Low
Frost, 2023	High	Low	Low	Low	High	High
Lu, 2023	Low	Low	Low	Low	Some concerns	Some concerns
Nazar, 2023	Low	High	High	Low	Low	High
Xue, 2022	Low	Low	Low	Low	Low	Low
Li, 2019	Low	Low	Low	Low	Low	Low
Tülübaş, 2019	Some concerns	Low	Low	Low	Low	Some concerns
Barr, 2018	Low	Low	Low	Low	Low	Low
Kawahara, 2015	Low	Low	Low	Low	Low	Low
Calle, 2014	Low	Low	Low	Low	Low	Low
Sinha, 2013	Low	Low	Low	Low	Low	Low

Supplemental table SA.6 Regional Anesthesia vs Sham: Summary of Findings

Quality assessment	Results
**Number of studies**<br>**n analyzed**	
24-hour pain scores	
12 RCTs<br>n= 1978	Cataldo, 2024 reported a 24-hour pain score with a median of 2 (IQR 0–3.75) in the intervention group and 1 (IQR 0–2) in the control group.<br>Gungorduk, 2024 reported mean 24-hour pain scores of 1.9 (SD 0.5) in the intervention group and 2.1 (SD 0.8) in the control group.<br>Salmonsen, 2024 reported postoperative day 1 pain scores with a mean of 4.1 (97.5% CI 3.5–4.6) in the control group; the mean differences for the two intervention groups were −0.4 (97.5% CI −1.1 to 0.3; p = 0.25) for I1 and −0.5 (97.5% CI −1.2 to 0.3; p = 0.15) for I2.<br>Lu, 2023 reported mean pain scores extracted from a figure, with 0.58 (SD 0.73) in the intervention group and 1.34 (SD 1.03) in the control group.<br>Frost, 2023 did not report 24-hour pain outcomes.<br>Nazar, 2023 reported median pain scores of 2 (IQR 1–2) in the intervention group and 4 (IQR 1–2.5) in the control group (p = 0.001).<br>Xue, 2022 reported mean pain scores of 0.7 (SD 1.2) in the TAP block group, 0.6 (SD 1.0) in the QLB group, and 1.0 (SD 1.1) in the control group, with no significant differences between study arms.<br>Li, 2019 reported median resting pain scores of 2 (IQR 1–3) in the intervention group and 2 (IQR 2–3) in the control group, with no statistically significant difference (p > 0.05).<br>Tülübaş, 2019 reported mean pain scores of 1.77 in the intervention group and 2.56 in the control group (p = 0.489).<br>Barr, 2018 reported mean pain scores of 4.5 in the intervention group and 4.6 in the control group, with no statistically significant difference.<br>Kawahara, 2015 reported four-grade pain scores with a median of 1 (range 0–2) in both intervention and control groups (p = 0.23).

Quality assessment	Results
**Number of studies**<br>**n analyzed**	
	Calle, 2014 reported mean pain scores of 3.7 in the intervention group and 4.4 in the control group (p = 0.237).<br>Sinha, 2013 reported median pain scores of 0 (range 0–2) in the intervention group and 1 (range 0–2) in the control group (p = 0.012).
**48 hours: Pain scores**	
4 RCTs<br>n=751	Lu, 2023 reported mean pain scores extracted from a figure, with 0.07 (SD 0.29) in the intervention group and 0.51 (SD 0.76) in the control group.<br>Xue, 2022 reported 48-hour mean pain scores of 0.3 (SD 0.8) in the TAP block group, 0.3 (SD 0.9) in the QLB group, and 0.3 (SD 0.8) in the control group, with no significant differences between arms.<br>Barr, 2018 reported postoperative day 2 mean pain scores of 3.9 in the intervention group and 3.8 in the control group, with no statistically significant difference.<br>Calle, 2014 reported 48-hour pain scores of 2.95 in the intervention group and 3.00 in the control group (p = 0.702).
**24 hours: Total postoperative opioid use**	
7 RCTs<br>n = 626	Cataldo, 2024 reported mean 24-hour opioid consumption of 0.45 mg OME (SD 1.59) in the intervention group and 56 mg OME (SD 0.42) in the control group, with no statistically significant difference (p > 0.99).<br>Salmonsen, 2024 reported median 24-hour opioid consumption of 57.6 mg (IQR 20.4–101.4) for intervention group I1 and 47.4 mg (IQR 24.9–75.6) for intervention group I2, compared with 66 mg (IQR 39.9–99.9) in the control group; I1 did not differ significantly from control (p = 0.55), while I2 consumed significantly less opioid than control (p = 0.01).<br>Frost, 2023 reported median opioid consumption of 13.5 mg (IQR 5.0–20.0; range 0–40) in the intervention group, compared

Quality assessment	Results
**Number of studies n analyzed**	
	with 10.0 mg (IQR 0.0–15.5; range 0–35) in control group C1 and 10.0 mg (IQR 7.5–20.0; range 0–50) in control group C2, with no statistically significant difference (p = 0.1).<br>Nazar, 2023 reported mean opioid consumption of 50.19 mg OME (SD 13.20) in the intervention group and 67.95 mg OME (SD 12.57) in the control group, with a statistically significant difference (p = 0.001).<br>Li, 2019 reported median opioid consumption of 39.6 mg (IQR 24–79.8) in the intervention group and 32.4 mg (IQR 23.4–65.1) in the control group, with no significant difference (p = 0.311).<br>Tülübaş, 2019 reported mean opioid consumption of 3.96 mg OME (SD 2.58; median 3.6) in the intervention group and 4.28 mg OME (SD 2.77; median 3.6) in the control group, with no significant difference (p = 0.65).<br>Sinha, 2013 reported mean opioid consumption of 2.7 mg in the intervention group and 14.4 mg in the control group, with a statistically significant difference (p = 0.00).
**Additional opioid metrics for studies that do not report a cumulative opioid consumption**	
4 RCTs<br>n = 545	One RCT reported mean total opioid consumption including intraoperative opioids and found no significant difference between groups (Calle, 2014).<br><br>One RCT reported the mean number of effective PCA requests and found no significant difference when comparing TAP block vs. sham. However, there was a significant difference favoring QLB (Xue, 2022).<br><br>One RCT reported the number of patients using narcotics at specific postoperative days, mean number of opioid tablets consumed by POD 14, and median number of postoperative days

Quality assessment	Results
**Number of studies**<br>**n analyzed**	
	on opioid analgesics and found no significant difference (Barr, 2018)<br><br>One RCT reported the proportion of patients receiving rescue tramadol and found a significant difference favoring intervention. (Gungorduk, 2024)<br>I: 6 (7.8%)<br>C: 16 (21.3)<br>p=0.021
**Patient satisfaction**	
2 RCT<br>n=297	Sinha 2013 reported median patient satisfaction score and favored the intervention. Median satisfaction score (min-max):<br>I: 7.0 (5.0- 8.0); n=50<br>C: 6.0 (5.0- 7.0); n=50<br>p<0.001<br><br>Lu 2023 reported proportion of satisfied patients and found a significant difference favoring intervention.<br>C: 32 (31.68%)<br>I: 75 (78.13%)<br>p< 0.05
**Hospital length of stay**	
4 RCTs<br>n = 677	Li, 2019 reported a mean hospital stay of 4.00 days (SD 0.61) in the intervention group and 4.00 days (SD 0.46) in the control group, with a mean difference of 0.00 days (95% CI −0.21 to 0.21).<br>Xue, 2022 reported mean stays of 3.65 days (SD 1.21) in the intervention group and 3.50 days (SD 0.70) in the control group, with a mean difference of 0.15 days (95% CI −0.10 to 0.40).<br>Lu, 2023 reported mean stays of 3.06 days (SD 0.24) in the intervention group and 3.15 days (SD 0.43) in the control group, with a mean difference of −0.09 days (95% CI −0.19 to 0.01).

Quality assessment	Results
**Number of studies<br>n analyzed**	
	Gungorduk, 2024 reported mean stays of 1.00 day (SD 0.001) in both intervention and control groups, with a mean difference of 0.00 days (95% CI −0.00 to 0.00).
**Intervention associated harms**	
11 RCTs<br>n = 1978	Cataldo, 2024 reported postoperative nausea and vomiting at 24 hours in 13% of the intervention group and 14% of the control group (p = 0.914).<br>Gungorduk, 2024 reported no complications or adverse effects related to the use of bupivacaine.<br>Salmonsen, 2024 reported nausea in 37.8% of controls, 38.9% of the I1 group (p = 0.47), and 34.4% of the I2 group (p = 0.62); vomiting occurred in 22% of controls, 23.2% of the I1 group (p = 0.45), and 22.2% of the I2 group (p = 0.93).<br>Lu, 2023 reported adverse reactions—including nausea, vomiting, hypotension, bradycardia, and others—in 5.21% of the intervention group and 17.82% of the control group (p = 0.006).<br>Nazar, 2023 reported postoperative nausea and vomiting in 42.8% of the intervention group and 23.0% of the control group, with no statistically significant difference per study authors; pruritus occurred in 5.7% of the intervention group and 3.0% of controls, also with no significant difference.<br>Xue, 2022 reported inpatient postoperative nausea and vomiting in 31.6% of the I1 group, 28.9% of the I2 group, and 35.6% of the control group (p = 0.918).<br>Li, 2019 reported 24-hour postoperative nausea and vomiting in 32.7% of the intervention group and 29.4% of the control group (p = 0.719).<br>Tülübaş, 2019 reported 24-hour nausea in 25% of the intervention group and 44.7% of the control group (p = 0.2); vomiting was assessed in a figure, and no statistically significant difference was reported.

Quality assessment	Results
**Number of studies**<br>**n analyzed**	
	Kawahara, 2015 reported nausea during the first 24 hours in 18.3% of the intervention group and 35.6% of the control group (p = 0.04).<br>Calle, 2014 reported no seizures or cardiovascular instability and stated that subtle signs of neurological toxicity were not prospectively assessed.<br>Sinha, 2013 reported that no adverse events related to the TAP block were observed.
**Discharge prescription amount/ refills**	
1 RCT<br>n=120	One RCT reported frequency of opioid refill requests and did not conduct a statistical test to compare frequency between groups.<br><br>Frost, 2023<br>n, %<br>I: 2/60, 3.33%<br>C: 0/60, 0%<br>No p-value reported
**Quality of recovery**	
2 RCTs<br>n = 492	Gungorduk, 2024 reported mean QoR- 40 and found a significant difference favoring intervention<br>I: 166.6 (11.9)<br>C: 161.9 (13.3)<br>p = 0.022<br><br>Salmonsen, 2024 reported mean QoR- 15 and found no significant difference.

Abbreviations: Abbreviations: C= comparator/control; CI= confidence interval; I= intervention; IQR= interquartile range; LOS= length of stay; MA= meta-analysis; mm=millimeters; n= number of participants; NR= not reported; p= p-value; PCA= patient controlled analgesia; POD= postoperative day; PONV= postoperative nausea and vomiting; RCT= randomized controlled trials; SD= standard deviation; SE= standard error; SMD = standard mean difference; SOE= strength of evidence; VAS= visual analogue scale

Supplemental table SA.7 Regional Anesthesia vs Local Anesthesia: Descriptive Characteristics of Studies

**Author, Year<br>Country<br>Study Design<br>Ambulatory or In patient<br>Risk of bias**	**Population<br>n randomized<br>Mean age (SD)<br>Sex (% female)<br>Race/ Ethnicity<br>Mean BMI (SD)**	**Intervention**	**Comparator**	**Outcomes of interest reported**
Mongelli, 2023<br>Switzerland<br>RCT<br>Inpatient<br>Some concerns	Patients undergoing laparoscopic GI surgery<br>n=113<br>Age:<br>I: 47.9 (10.1) y<br>C:48.0 (12.1) y<br>77% female<br>Race/ ethnicity NR<br>BMI:<br>I: 40.4 (5.9) kg/m^2<br>C: 40.5 (5.9) kg/m^2	TAP block	Port site infiltration	Pain scores (24 hr)<br>Total postoperative opioid consumption (24 hr)<br>Hospital length of stay<br>Patient satisfaction<br>Postoperative complications
Ren, 2022<br>China<br>RCT<br>Inpatient<br>Low	Patients undergoing laparoscopic Colorectal Cancer Resection<br>n= 160<br>Age:<br>I: 60.3 (7.2)<br>C: 61.0 (8.4)<br>% female<br>I: 50.7%<br>C: 49.4%	TAP block	Local wound infiltration	Pain scores (24, 48, 72 hrs)<br>Hospital length of stay<br>Patient satisfaction<br>Postoperative complications

	NR<br>BMI<br>21.6 (2.5)<br>22.1 (3.2)			
Ruiz-Tovar, 2018<br>Spain<br>RCT<br>Inpatient<br>Some concerns	Patients undergoing laparoscopic GI surgery<br>n=140<br>Age:<br>I: 41.9 (5.9) y<br>C: 41.7 (7.2) y<br>57.1% female<br>Race/ ethnicity NR<br>BMI:<br>I: 47.4 (5.2) kg/m^2<br>C: 46.5 (4.3) kg/m^2	TAP block	Port site infiltration	Pain scores (24 hr)<br>Hospital length of stay<br>Postoperative complications<br>Rescue opioid metrics

Abbreviations: BMI= body mass index; C: control/comparator; CI: confidence interval; d=day(s); d/c=discharge; GI=gastrointestinal; gyn=gynecologic; hr=hour(s); I=intervention; kg=kilogram; m=meter(s); n=number of participants; NR=not reported; OME=oral morphine equivalents; RCT= randomized controlled trial; SD=standard deviation; p=p-value; y=year(s)

Supplemental table SA.8 Regional Anesthesia vs Local Anesthesia: Risk of Bias

**Author, Year**	**Bias from randomization process**	**Bias from deviation from intended interventions (assignment)**	**Bias due to missing outcome data**	**Bias in measurement of outcome**	**Bias in selection of reported results**	**Overall risk of bias (low, some concerns, high)**
Mongelli, 2023	Some concerns	Low	Low	Low	Low	Some concerns
Ren, 2022	Low	Low	Low	Low	Low	Low
Ruiz-Tovar, 2018	Some concerns	Some concerns	Low	Low	Low	Some concerns

Supplemental table SA.9 Regional Anesthesia vs Local Anesthesia: Summary of Findings

Quality assessment	Results
**Number of studies**<br>**n analyzed**	
**Pain scores (24 hours)**	
3 RCTs<br>n= 398	Mongelli, 2023 reported mean 24-hour pain scores of 2.5 (SD 2.6) in the intervention group and 2.3 (SD 2.1) in the control group, with no statistically significant difference (p = 0.661).<br>Ren, 2022 reported mean 24-hour pain scores of 2 (IQR 1–3) in the intervention group and 2 (IQR 1–4) in the control group, with no significant difference (p = 0.638).<br>Ruiz-Tovar, 2018 reported 24-hour pain scores on a 0–100 scale, with mean values of 10 (SD 8.1) in the intervention group and 16.8 (SD 11.2) in the control group, a statistically significant difference (p = 0.001).
**Pain scores (48 hours)**	
2 RCTs<br>n= 285	Ruiz-Tovar, 2018 reported means and found a significant difference favoring the intervention.<br>Mean (SD):<br>I: 1 (0.81)<br>C: 1.68 (1.12)<br>p=0.001<br><br>Ren 2022 reported median pain scores, found no significant difference.
**Cumulative postoperative opioid use (24 hours)**	
1 RCT<br>n = 113	Mongelli, 2023 reported morphine milligram equivalents - unclear if oral and found no significant difference.
**Hospital LOS**	
3 RCTs<br>n = 398	Mongelli, 2023 reported a mean hospital stay of 3.4 days (SD 1.5) in the intervention group and 3.2 days (SD 1.1) in the control group, with no statistically significant difference (p = 0.392).

Quality assessment<br>**Number of studies<br>n analyzed**	Results
	Ren, 2022 reported a median stay of 6 days (IQR 4–7.5) in the intervention group and 6 days (IQR 5–7) in the control group, with no significant difference (p = 0.843).<br>Ruiz-Tovar, 2018 reported a mean hospital stay of 2.1 days (SD 1.2) in the intervention group and 2.9 days (SD 1.3) in the control group, a statistically significant difference (p = 0.019).
**Patient satisfaction with analgesia**	
1 RCT<br>n= 148	Ren, 2022 reported no significant difference in satisfaction scores.
**Perioperative patient satisfaction**	
1 RCT<br>n=113	Mongelli, 2023 reported Leiden Perioperative care Patient Satisfaction scores and found no significant difference in overall scores.
**Intervention associated harms (PONV)**	
1 RCTs<br>n= 148	Ren, 2022 reported PONV proportions and found no significant differences.
**Opioid rescue metrics (only studies that did not report cumulative postoperative opioid consumption)**	
2 RCTs<br>n = 285	Ruiz-Tovar, 2018 reported the proportion of participants requiring opioid rescue and found a significant difference favoring intervention.<br>I: (nr), 2.9%<br>C: (nr), 13.2%<br>p=0.0270*

Quality assessment<br>Number of studies<br>n analyzed	Results
	Ren, 2022 reported the proportion of participants which had PCIA bolus of opioid rescue at different time intervals and found no significant difference.

Abbreviations: C= comparator/control; CI= confidence interval; I= intervention; IQR= interquartile range; LOS= length of stay; MA= meta-analysis; mm=millimeters; n= number of participants; NR= not reported; p= p-value; PCA= patient controlled analgesia; POD= postoperative day; PONV= postoperative nausea and vomiting; RCT= randomized controlled trials; SD= standard deviation; SE= standard error; SMD = standard mean difference; SOE= strength of evidence; VAS= visual analogue scale

* Calculated by ERT

[SIIB] Preoperative intraperitoneal or preperitoneal instillation of local anesthetics

Supplemental table SB.1 Intraperitoneal or preperitoneal instillation of LA vs sham/ no intervention: Descriptive Characteristics of Studies

Author, Year<br>Country<br>Study Design<br>Ambulatory or Inpatient<br>Risk of bias	Population<br>Subgroups<br>n randomized<br>Mean age (SD)<br>Sex (% female)<br>Race/ Ethnicity<br>Mean BMI (SD)	Intervention	Comparator	Outcomes of interest reported
Zheng, 2023<br>Malaysia<br>RCT<br>Inpatient<br>Low	Laparoscopic GI surgical patients<br>Excluded those with chronic pain diagnoses, inclusion of chronic opioid use and psychiatric diagnoses NR<br>n=110<br>Median (IQR) I:36.0 (9.0) y; C: 40.0 (13.0) y<br>I: 73% female; C: 71% female<br>NR<br>Median(IQR) I:39.8 (10.79) kg/m^2; C: 40.8 (10.54) kg/m^2	Intraperitoneal instillation of ropivacaine	Intraperitoneal instillation of normal saline	Pain scores (24hr)<br>Opioid rescue analgesic metrics<br>Drug associated harms
Aldohayan, 2022<br>Saudi Arabia<br>RCT<br>Inpatient<br>Low	Laparoscopic GI surgical patients<br>Excluded those with chronic opioid use, inclusion of chronic pain diagnoses and psychiatric diagnosis NR<br>n=120<br>I: 35.48 y; C:33.18 y<br>61.7% female<br>NR	Pre-incisional laparoscopic preperitoneal local anesthetic technique with bupivacaine	Normal saline	Pain scores on mobilization (12hr)<br>Hospital length of stay<br>Opioid rescue analgesic metrics<br>Drug associated harms

Author, Year<br>Country<br>Study Design<br>Ambulatory or Inpatient<br>Risk of bias	Population<br>Subgroups<br>n randomized<br>Mean age (SD)<br>Sex (% female)<br>Race/ Ethnicity<br>Mean BMI (SD)	Intervention	Comparator	Outcomes of interest reported
	I: 46.97 kg/m^2; C: 45.10 kg/m^2			
Kaur, 2022<br>Australia<br>RCT<br>Inpatient<br>High	Laparoscopic GI surgical patients<br>Included chronic opioid use and chronic pain diagnoses, inclusion of psychiatric diagnosis NR<br>n=120<br>Median (IQR): 41.0 (29.8–50.0) y<br>78.8% female<br>Ethnicity n(%): Aboriginal/Torres Strait Islander 10 (9.6); Caucasian 94 (90.4)<br>Median (IQR): 40.9 (36.8–46.1) kg/m^2	Ropivacaine sprayed on to the diaphragm using mixing cannula	Normal saline sprayed on the diaphragm using mixing cannula	Pain scores (24 hr, 48hr)<br>Opioid rescue analgesic metrics<br>Hospital length of stay
Cunningham, 2020<br>UK<br>RCT<br>Inpatient<br>Low	Laparoscopic Gyn surgical patients<br>Inclusion of chronic opioid use, chronic pain diagnoses, and psychiatric diagnoses NR<br>n=100<br>I: 41 (13) y; C: 40 (12) y<br>100% female<br>NR<br>NR	Intraperitoneal instillation of levobupivacaine	Intraperitoneal instillation of saline	Pain Scores (24 hr, 96 hr)<br>Opioid rescue analgesic metrics
Manan, 2020<br>Pakistan	Laparoscopic GI surgical patients	Irrigation of bupivacaine	Irrigation of normal saline	Pain Scores(24 hr)

**Author, Year**<br>**Country**<br>**Study Design**<br>**Ambulatory or Inpatient**<br>**Risk of bias**	**Population**<br>**Subgroups**<br>**n randomized**<br>**Mean age (SD)**<br>**Sex (% female)**<br>**Race/ Ethnicity**<br>**Mean BMI (SD)**	**Intervention**	**Comparator**	**Outcomes of interest reported**
RCT<br>Inpatient<br>High	Excluded chronic opioid use and chronic pain diagnoses, inclusion of psychiatric diagnoses NR<br>n=110<br>I: 44.22 (10.45) y; C: 44.42 (10.78) y<br>55% female<br>NR<br>NR			Total postoperative opioid consumption (24 hr)
Safari, 2020<br>Iran<br>RCT<br>Inpatient<br>Low	Laparoscopic GI surgical patients<br>Excluded those with opioid use within 24 hours before surgery and those with chronic pain diagnoses, inclusion of comorbid psychiatric diagnoses NR<br>n=109<br>I: 37.5 y; C: 36.4 y<br>NR<br>NR<br>I: 43.7 6 kg/m^2; C: 45 kg/m^2	Intraperitoneal instillation of bupivacaine	Intraperitoneal instillation of normal saline	Pain Scores (24 hr)<br>Opioid rescue analgesic metrics
Schipper, 2019<br>Netherlands<br>RCT<br>Inpatient<br>Low	Laparoscopic GI surgical patients<br>Ran a subgroup analysis excluding patients with chronic use of analgesics, inclusion of	Bupivacaine was sprayed onto the left side of the diaphragm	No local anesthesia	Pain Scores (24hr)<br>Opioid rescue analgesic metrics<br>Hospital length of stay

**Author, Year**<br>**Country**<br>**Study Design**<br>**Ambulatory or Inpatient**<br>**Risk of bias**	**Population**<br>**Subgroups**<br>**n randomized**<br>**Mean age (SD)**<br>**Sex (% female)**<br>**Race/ Ethnicity**<br>**Mean BMI (SD)**	**Intervention**	**Comparator**	**Outcomes of interest reported**
	chronic pain diagnosis and psychiatric diagnoses NR<br>n=130<br>I:46.2 y; C: 42.3 y<br>I: 86.45% female; C: 83.6% female<br>NR<br>I: 42.1 kg/m^2; C: 42.5 kg/m^2			
Jairath, 2017<br>India<br>RCT<br>Inpatient<br>Low	Laparoscopic Urology surgical patients<br>Inclusion of chronic opioid use, chronic pain diagnoses, and psychiatric diagnoses NR<br>n=100<br>I: 48.52 (10.18) y; C: 45.92 (08.79)<br>72% female<br>NR<br>NR	Intraperitoneal instillation of bupivacaine	Instillation of normal saline	Pain scores (24 hr)<br>Total postoperative opioid consumption (24 hr)<br>Drug associated harms
Ruiz-Tovar, 2016<br>Spain<br>RCT<br>Inpatient<br>Some concerns	Laparoscopic GI surgical patients<br>Inclusion of chronic opioid use, chronic pain diagnoses, and psychiatric diagnoses NR<br>n=110<br>I: 44.6 y; C: 46.5 y<br>68.2% female<br>NR	Intraperitoneal ropivacaine irrigation	Intraperitoneal normal saline irrigation	Pain scores (24 hr)<br>Opioid rescue analgesic metrics<br>Hospital length of stay<br>Drug associated harms

Author, Year<br>Country<br>Study Design<br>Ambulatory or Inpatient<br>Risk of bias	Population<br>Subgroups<br>n randomized<br>Mean age (SD)<br>Sex (% female)<br>Race/ Ethnicity<br>Mean BMI (SD)	Intervention	Comparator	Outcomes of interest reported
	I: 46.9 (9.6) kg/m^2; C: 44.7 (5.9) kg/m^2			
Arden, 2013<br>USA<br>RCT<br>Inpatient<br>High	Laparoscopic Gyn surgical patients<br>Excluded chronic opioid use, inclusion of chronic pain diagnoses and psychiatric diagnoses NR<br>n=160<br>I: 44.1 (6.4) y; C: 44.9 (7.1) y<br>100% female<br>I: White- 87%,<br>Other- 14%<br>C: White - 89%,<br>Other- 11%<br>I: 27.2 kg/m^2; C: 27.3 kg/m^2	Intraperitoneal instillation of 100 mg bupivacaine	Instillation of 100 ml of saline	Pain scores (24 hr)<br>Total postoperative opioid consumption (24hr)<br>Hospital length of stay<br>Patient satisfaction<br>Drug associated harms

Abbreviations: BMI= body mass index; C=control/comparator; GI= gastrointestinal; Gyn= gynecological; hr(s)= hour(s); I= Intervention; IQR= interquartile range; mg=milligram(s); mL=milliliter(s); n=number of participants; NR= not reported; OME=oral morphine equivalents; p=p-value; RCT = randomized controlled trials; USA=United States of America; y=year(s)

Supplemental table SB.2 Intraperitoneal or preperitoneal instillation of LA vs sham/ no intervention: Risk of Bias

Author, Year	Bias from randomization process	Bias from deviation from intended interventions (assignment)	Bias due to missing outcome data	Bias in measurement of outcome	Bias in selection of reported results	Overall risk of bias (low, some concerns, high)
Zheng, 2023	Low	Low	Low	Low	Low	Low
Aldohayan, 2022	Low	Low	Low	Low	Low	Low
Kaur, 2022	Low	Low	High	Low	Low	High
Cunningham, 2020	Low	Low	Low	Low	Low	Low
Manan, 2020	Some concerns	Some concerns	Low	High	Low	High
Safari, 2020	Some concerns	Low	Low	Low	Low	Low
Schipper, 2019	Low	Low	Low	Low	Low	Low
Jairath, 2017	Some concerns	Low	Low	Low	Low	Some concerns
Ruiz-Tovar, 2016	Some concerns	Low	Low	Some concerns	Low	Some concerns
Arden, 2013	Low	High	High	Low	Low	High

Supplemental table SB.3 Intraperitoneal or preperitoneal instillation of LA vs sham/ no intervention: Summary of Findings

Quality assessment	Results
Number of studies<br>n analyzed	
24-hour pain scores	

**Quality assessment**	**Results**
**Number of studies**<br>**n analyzed**	
9 RCTs<br>n= 971	Ruiz-Tovar, 2016 reported mean pain scores of 1.33 (SD 1.09) in the intervention group and 2.17 (SD 1.45) in the control group, with a mean difference of −0.84 (95% CI −1.32 to −0.36).<br>Schipper, 2019 reported mean scores of 2.83 (SD 0.23) in the intervention group and 2.79 (SD 0.23) in the control group, with a mean difference of 0.04 (95% CI −0.04 to 0.12).<br>Cunningham, 2020 reported mean scores of 3.10 (SD 2.50) in the intervention group and 3.60 (SD 2.50) in the control group, with a mean difference of −0.50 (95% CI −1.49 to 0.49).<br>Manan, 2020 reported mean scores of 4.44 (SD 1.03) in the intervention group and 4.80 (SD 0.99) in the control group, with a mean difference of −0.36 (95% CI −0.74 to 0.02).<br>Safari, 2020 reported mean scores of 2.50 (SD 1.00) in the intervention group and 3.40 (SD 1.20) in the control group, with a mean difference of −0.90 (95% CI −1.32 to −0.48).<br><br>Zheng, 2023 reported median pain scores of 2 (IQR 1) in the intervention group and 3 (IQR 2) in the control group, with a statistically significant difference (p = 0.002).<br>Arden, 2013 reported median pain scores of 2.2 (IQR 1.1–4.6) in the intervention group and 2.0 (IQR 0.9–4.3) in the control group, with no significant difference (p = 0.36).<br>Kaur, 2022 reported median pain scores of 2 (IQR 0.8–3) in the intervention group and 2 (IQR 1–4.3) in the control group, with no significant difference (p = 0.23).<br>Jairath, 2017 reported mean pain scores of 1.46 in the intervention group and 1.7 in the control group, with no significant difference noted [Data in figure only, SD NR].
**24 hour: total postoperative opioid consumption (OME)**	

Quality assessment	Results
**Number of studies**<br>**n analyzed**	
3 RCT<br>n = 367	Manan, 2020 reported mean opioid consumption of 9.30 OME (SD 4.49) in the intervention group and 37.44 OME (SD 8.06) in the control group, with a mean difference of −28.14 OME (95% CI −30.58 to −25.70).<br><br>Jairath, 2017 reported mean opioid consumption of 16.50 OME (SD 13.00) in the intervention group and 31.00 OME (SD 14.00) in the control group, with a mean difference of −14.50 OME (95% CI −19.80 to −9.20).<br><br>Arden, 2013 reported mean opioid consumption of 73.09 OME (SD 62.78) in the intervention group and 89.90 OME (SD 74.29) in the control group, with a mean difference of −16.81 OME (95% CI −38.30 to 4.68).
**Opioid rescue analgesics (only for studies that did not report a total postoperative opioid consumption)**	
7 RCTs<br>n=777	Zheng, 2023 reported that 24% of patients in the intervention group and 42% in the control group required additional intravenous tramadol (p = 0.041); data on the number of analgesic requests and total opioid amount were not reported.<br>Aldohayan, 2022 reported mean PACU morphine use of 2.95 mg (SD 0.39) in the intervention group and 6.0 mg (SD 0.4) in the control group (p < 0.0001), corresponding to 8.85 OME (SD 1.17) versus 18 OME (SD 1.2); the study did not report the number of patients requiring opioids or the number of analgesic requests.<br>Kaur, 2022 reported a mean frequency of opioid-analgesia requests of 1.9 (95% CI 1.56–2.29) in the intervention group and 1.8 (95% CI 1.5–2.1) in the control group, with no p-value

Quality assessment	Results
**Number of studies**<br>**n analyzed**	
	reported; outcomes on the number of patients requiring opioids and total opioid dose were not provided.<br>Cunningham, 2020 reported no significant difference between groups in the need for postoperative analgesia, including opioid versus non-opioid requirements; figure-only data indicated no meaningful group differences in the number of analgesic requests at multiple time intervals, and total opioid dose was not reported.<br>Safari, 2020 reported that 1 of 52 intervention patients and 0 of 54 control patients required opioid rescue analgesia, and also reported significantly lower 24-hour pethidine use in the intervention group (20.64 OME, SD 5.88) compared with controls (31.7 OME, SD 4.05; $p < 0.001$).<br>Schipper, 2019 reported opioid use in 68.2% of intervention patients and 78.7% of control patients ($p = 0.184$), with no data reported on analgesic request frequency or opioid dose.<br>Ruiz-Tovar, 2016 reported morphine requirements within 24 hours in 3.6% of patients in the intervention group and 21.8% in the control group ($p = 0.01$), while the number of analgesic requests and total opioid amount were not reported.
**Hospital length of stay**	
5 RCT<br>n=617	Aldohayan, 2022 reported a mean length of stay of 0.58 days in the intervention group and 1.2 days in the control group, with no statistically significant difference ($p = 0.1$).<br>Kaur, 2022 reported a median stay of 1 day (IQR 1–2) in both the intervention and control groups, with no significant difference ($p = 0.63$).<br>Schipper, 2019 reported a mean stay of 1 day in both the intervention and control groups ($p = 0.62$).<br>Ruiz-Tovar, 2016 reported a median stay of 2 days (range 2–7) in the intervention group and 3 days (range 2–10) in the control group, with a statistically significant difference favoring the intervention ($p = 0.0009$).

Quality assessment<br>Number of studies<br>n analyzed	Results
	Arden, 2013 reported median lengths of stay of 23.3 hours (IQR 20.5–24.8) in the intervention group and 23 hours (IQR 21.5–25.8) in the control group, with no significant difference (p = 0.49).
**Patient satisfaction**	
1 RCT<br>n=136	Arden 2013 reported median, found no difference
**Drug-associated harms**	
5 RCT<br>n=597	Zheng, 2023 reported similar rates of nausea in the intervention and control groups (33% vs 38%, p = 0.550) and similar rates of vomiting (33% vs 36%, p = 0.688).<br>Aldohayan, 2022 reported postoperative nausea and vomiting in 35% of patients in the intervention group compared with 53.3% in the control group, a statistically significant difference (p = 0.04).<br>Jairath, 2017 stated that adverse effects—including nausea, vomiting, itching, bradycardia, shoulder pain, hypotension, and shivering—were less frequent in the intervention group, but the differences were not statistically significant.<br>Ruiz-Tovar, 2016 reported nausea or vomiting in 16.4% of intervention patients and 3.6% of control patients, with the difference approaching but not reaching statistical significance (p = 0.056).<br>Arden, 2013 reported one control-group readmission due to nausea and vomiting and no such readmissions in the intervention group; no p-value was provided.

Abbreviations: C=control/comparator; CI= confidence intervals; I= Intervention; IQR= interquartile range; MA=meta-analysis; n=number of participants; OME=oral morphine equivalents; p=p-value; RCT = randomized controlled trials; SOE = strength of evidence

*Reasons for exclusion from meta-analysis: Arden, 2013 was not included because it was significantly skewed and therefore could not be converted to mean; Kaur 2022, Jairath 2017, and Zheng 2023 did not report a measure of variance.

**Reasons for exclusion from meta-analysis: Kaur, 2022 and Ruiz-Tovar, 2016 were significantly skewed and therefore could not be converted to mean; Aldohayan, 2022 did not report a standard deviation

Supplemental table SB.4 Intraperitoneal or preperitoneal instillation of LA plus incisional local infiltration vs no intervention/sham: Descriptive Characteristics of Studies

**Author, Year**<br>**Country**<br>**Study Design**<br>**Ambulatory or Inpatient**<br>**Risk of bias**	**Population**<br>**Subgroups**<br>**n randomized**<br>**Mean age (SD)**<br>**Sex (% female)**<br>**Race/ Ethnicity**<br>**Mean BMI (SD)**	**Intervention**	**Comparator**	**Outcomes of interest reported**
Abet, 2017<br><br>RCT<br>Inpatient<br>Some concerns	Laparoscopic GI surgical patients<br>Excluded those with chronic pain diagnoses, inclusion of chronic opioid use and psychiatric diagnoses NR<br>n=102<br>Mean (SD) I: 49.9 (15.3) y; C: 45.7 (13.4) y<br>I: 68% female; C: 70% female<br>NR<br>NR	Intraperitoneal instillation and wound infiltration with ropivacaine	No intervention	Pain scores (24hr)<br>Opioid rescue analgesic metrics<br>Harms
Ismail, 2013<br><br>RCT<br>Inpatient<br>High	Laparoscopic Gynecological surgical patients<br>NR in selection criteria: history of a major psychiatric disorder, chronic pain syndrome, morbid obesity, chronic opioid	Preincisional local infiltration and intraperitoneal instillation levobupivacaine 0.25%	Preincisional local infiltration and intraperitoneal instillation normal saline	Pain scores (24hr)<br>Opioid consumption (24hr)<br>Hospital length of stay<br>Patient satisfaction<br>Harms

Author, Year<br>Country<br>Study Design<br>Ambulatory or Inpatient<br>Risk of bias	Population<br>Subgroups<br>n randomized<br>Mean age (SD)<br>Sex (% female)<br>Race/ Ethnicity<br>Mean BMI (SD)	Intervention	Comparator	Outcomes of interest reported
	treatment, and current opioid use.<br>n=106<br>Mean (SD) I: 26.9 (1.9) y; C: 26.7 (1.8) y<br>100% female<br>NR<br>Median(IQR) I: 26.4 (2.7) kg/m^2; C: 26.3 (2.9) kg/m^2			

Supplemental table SB.5 Intraperitoneal or preperitoneal instillation of LA plus incisional local infiltration vs no intervention/sham: Risk of Bias

Author, Year	Bias from randomization process	Bias from deviation from intended interventions (assignment)	Bias due to missing outcome data	Bias in measurement of outcome	Bias in selection of reported results	Overall risk of bias (low, some concerns, high)
Abet, 2017	Some concerns	Low	Low	Low	Low	Some concerns
Ismail, 2013	Low	Low	Low	High	Low	High

Supplemental table B.6 Intraperitoneal or preperitoneal instillation of LA plus incisional local infiltration vs no intervention/sham: Summary of Findings

**Quality assessment**	**Results**
**Number of studies**<br>**n analyzed**	
**24 hour pain scores**	
1 RCT<br>n=100	Abet, 2019<br>Mean (SD)<br>I: 1 (NR)<br>C: 1 (NR)<br>P-value: 0.98
**24 hour total opioid consumption**	
1 RCT<br>n=106	Ismail, 2013<br>Mean (SD)<br>I: 129.4 (21.7) C: 168.3(22.9)<br>p<0.001
**Hospital length of stay**	
1 RCT<br>n=106	Ismail, 2013<br>I: 25.57 (2.65) C: 28.27 (3.22)<br>p<0.001
**Adverse events**	
2 RCTs<br><br>n=206	No significant differences (nausea, vomiting) in both RCTs
**Patient satisfaction**	
1 RCT	Ismail, 2013

n=106	I: 3.3 (0.5)<br>C: 2.9 (0.6)<br>p<0.001

[SIIC] Incisional local anesthetics

Supplemental table SC.1 Incisional Local Anesthetics: Descriptive Characteristics of Studies

**Author, Year**<br>**Country**<br>**Study Design**<br>**Ambulatory or Inpatient**<br>**Risk of bias**	**Population**<br>**Subgroups**<br>**n randomized**<br>**Mean age (SD)**<br>**Sex (% female)**<br>**Race/ Ethnicity**<br>**Mean BMI (SD)**	**Intervention**	**Comparator**	**Outcomes of interest reported**
Berlit, 2015<br>Germany<br>RCT<br>Inpatient<br>Low	Gynaecologic laparoscopic surgery, patients who consumed regular analgesic medications were excluded<br>n= 121<br>Age- I: 43.07 (12.55)<br>C: 40.11 (10.06)<br>Sex - 100%<br>NA<br>BMI - I: 25.96 (4.69)<br>C: 26.96 (6.29)	EMLA patch on the abdominal incision	Placebo patch on the abdominal incision	24hr Pain scores<br>48-hour Pain scores<br>Requirement of Opioid Rescue Analgesia<br>Hospital Length of Stay.
Lin, 2015<br>China<br>RCT<br>Unclear<br>High	Patients with symptomatic gallstone disease were included. Patients with chronic pain syndrome or chronic narcotic use are excluded.<br>n= 180<br>Age Mean* - I1: 56.6, C: 51.0<br>Sex (n) - I1: 42, C:35<br>NA<br>BMI Mean - I1: 24.7, C: 25.1	Trocar site infiltration of Bupivacaine before incision closure	No Intervention	24hr Pain scores<br>Requirement of Opiod Rescue Analgesia<br>Hospital Length of Stay<br>Drug-Associated Harms

**Author, Year**<br>**Country**<br>**Study Design**<br>**Ambulatory or Inpatient**<br>**Risk of bias**	**Population**<br>**Subgroups**<br>**n randomized**<br>**Mean age (SD)**<br>**Sex (% female)**<br>**Race/ Ethnicity**<br>**Mean BMI (SD)**	**Intervention**	**Comparator**	**Outcomes of interest reported**
	*Reported for groups A and C			
Fassoulaki, 2016<br>Greece<br>RCT<br>Unclear<br>High	Laparoscopic cholecystectomy, patients with chronic pain, or consumption of analgesics and/or calcium channel blockers during the last month are excluded.<br>n= 110<br>Age - I: 51 (11.2)<br>C: 48 (12.5)<br>Sex- I: 69.09%<br>C: 74.54%<br>NA<br>BMI- I: 27 (3.6)<br>C: 28 (5.3)	Wound infusion with local anesthetics (ropivacaine)	Wound infusion with normal saline	24 hr Pain Score, 48 hr Pain Scores,<br>24-hour Consumption of Opioid Rescue Analgesia
Ali, 2018<br>Pakistan<br>RCT<br>Inpatient<br>High	Laparoscopic cholecystectomy, patients who were using opioids daily were excluded.<br>n=120<br>Age - 39.91 (11.70)<br>Sex - 77.5%<br>NA<br>BMI - 25.72 (3.80)	Instillation of Bupivacaine into to port sites	Instillation of Normal Saline into to port sites	24hr pains scores<br>Opioid rescue analgesia consumption

Author, Year<br>Country<br>Study Design<br>Ambulatory or Inpatient<br>Risk of bias	Population<br>Subgroups<br>n randomized<br>Mean age (SD)<br>Sex (% female)<br>Race/ Ethnicity<br>Mean BMI (SD)	Intervention	Comparator	Outcomes of interest reported
Chowdhury, 2019<br>Bangladesh<br>RCT<br>Ambulatory<br>High	Laparoscopy,<br>Patients with chronic pelvic pain who have used NSAIDs and other analgesics for a long time are excluded.<br>n=144<br>Age -I: 29.7 (4.5)<br>C: 30.2 (4.7)<br>Sex -100%<br>NA<br>BMI -NA	Port site infiltration of Bupivacaine	Port site infiltration of no bupivacaine	12 hr pain scores, 7-day pain score<br>Requirement of opioid rescue analgesia<br>Adverse events
Zhang, 2019<br>China<br>RCT<br>Unclear<br>Low	Laparoscopic hepatectomy<br>Patients are excluded if they have a serious chronic pain disease, and cognitive impairment<br><br>n=139<br>Age - I: 52.87 (12.463)<br>C: 50.31 (13.984)<br>Sex- n I=31, C=29<br>NA<br>BMI - NA	Ropivacaine infiltration	Sham	24hr pains scores<br>48-hour pain scores<br>24-hour total opioid consumption<br>48-hour total opioid consumption<br>Hospital Length of Stay<br>Drug-Associated Harms and Complications

Author, Year<br>Country<br>Study Design<br>Ambulatory or Inpatient<br>Risk of bias	Population<br>Subgroups<br>n randomized<br>Mean age (SD)<br>Sex (% female)<br>Race/ Ethnicity<br>Mean BMI (SD)	Intervention	Comparator	Outcomes of interest reported
Herrador-Benito, 2024<br>Spain<br>RCT<br>Unclear<br>Some Concerns	Elective Laparoscopic cholecystectomy, Patients are excluded if they have cognitive impairment, and chronic pain disorders<br><br>n=242<br>Age - I: 55.4 (15.5)<br>C: 53.9 (14.9)<br>Sex- I: 69.2%, C:62.9%<br>NA<br>BMI - I: 28.3 (4.8), C: 27.4 (4.5)	Levobupivacaine Infiltration	No Intervention	24 hr pain scores<br>Requirement of opioid rescue analgesia<br>Hospital length of stay<br>Patient satisfaction<br>Function and quality of life outcomes<br>Drug-associated harms<br>complications

Supplemental Table SC.2 Incisional Local Anesthetics: Risk of Bias

Author, Year	Bias from the randomization process	Bias from deviation from intended interventions (assignment)	Bias due to missing outcome data	Bias in the measurement of outcome	Bias in the selection of reported results	Overall risk of bias (low, some concerns, high)
Berlit, 2015	Low	Low	Low	Low	Low	Low
Lin, 2015	Some Concerns	Some Concerns	Low	High	Some Concerns	High

Author, Year	Bias from the randomization process	Bias from deviation from intended interventions (assignment)	Bias due to missing outcome data	Bias in the measurement of outcome	Bias in the selection of reported results	Overall risk of bias (low, some concerns, high)
Fassoulaki, 2016	Low	High	Some Concerns	Low	Low	High
Ali, 2018	Some Concerns	Some Concerns	High	High	Low	High
Chowdhury, 2019	High	Some Concerns	Low	High	Some Concerns	High
Zhang, 2019	Low	Low	Low	Low	Low	Low
Herrador-Benito, 2024	Some Concerns	Low	Low	Low	Low	Some Concerns

Supplemental Table SC.3 Incisional Local Anesthetics: Summary of Findings

Quality assessment<br><br>Number of studies<br>n analyzed	Results
**24-hour pain scores VAS/NRS 0-10**	
6 RCTs<br>n= 811	Herrador-Benito, 2024 reported mean scores of 2.18 (SD 1.71) in the intervention group and 2.60 (SD 1.53) in the control group, with a mean difference of −0.42 (95% CI −0.86 to 0.02).<br>Zhang, 2019 reported mean scores of 1.54 (SD 0.50) in the intervention group and 1.63 (SD 0.49) in the control group, with a mean difference of −0.09 (95% CI −0.26 to 0.08).<br>Ali, 2018 reported mean scores of 3.97 (SD 1.13) in the intervention group and 3.90 (SD 1.10) in the control group, with a mean difference of 0.07 (95% CI −0.33 to 0.47).<br>Fassoulaki, 2016 reported mean scores of 1.40 (SD 1.60) in the intervention group and 1.50 (SD 2.00) in the control group, with a mean difference of −0.10 (95% CI −0.82 to 0.62).<br>Berlit, 2015 reported mean scores of 3.12 (SD 2.80) in the intervention group and 3.68 (SD 3.14) in the control group, with a mean difference of −0.56 (95% CI −1.62 to 0.50).<br>Lin, 2015 reported mean pain scores of 0.6 in both the intervention and control groups, with no statistically significant difference ($p > 0.05$).
**24 hr Opioid consumption**	
1 RCT<br>n= 136	Zhang, 2019 reported mean opioid consumption of 166.8 OME (SD 18.4) in the intervention group and 184.3 OME (SD 16.2) in the control group, a statistically significant difference ($p < 0.0001$).
**48 hr Opioid consumption**	
1 RCT<br>n= 136	Zhang, 2019 reported mean opioid consumption of 245.0 OME (SD 23.2) in the intervention group and 266.7 OME (SD 23.8) in the control group, a statistically significant difference ($p < 0.0001$).

**Quality assessment**	**Results**
**Number of studies**<br>**n analyzed**	
**Opioid Rescue Analgesics (only for studies where cumulative opioid consumption was not reported)**	
6 RCTs<br>n= 819	Berlit 2015; Lin, 2015; Fassoulaki, 2016; Ali, 2018; Chowdhury, 2019; Herrador-Benito, 2024 reported rescue opioid outcomes, of which Ali 2018 and Lin 2015 favored intervention and others reported no significant difference.
**Hospital Length of Stay**	
4 RCTs<br>n= 589	Zhang, 2019 reported a mean hospital stay of 7.28 days (SD 2.50) in the intervention group and 7.76 days (SD 2.99) in the control group, with a mean difference of −0.48 days (95% CI −1.41 to 0.45).<br>Berlit, 2015 reported a mean stay of 2.58 days (SD 0.70) in the intervention group and 2.52 days (SD 0.48) in the control group, with a mean difference of 0.06 days (95% CI −0.15 to 0.27).<br>Herrador-Benito, 2024 reported a mean stay of 1.05 days (SD 0.33) in the intervention group and 1.11 days (SD 0.40) in the control group, with a mean difference of −0.06 days (95% CI −0.16 to 0.04).<br>Lin 2015 reported a mean stay of 3.4 days in the intervention group vs 3.7 days in the control group (p=0.397; SD not reported)
**Drug-associated harms / Adverse Events**	
7 RCTs<br>n= 853	Herrador-Benito, 2024 reported nausea and vomiting in 19.6% of patients in the intervention group compared with 31.4% in the control group, a statistically significant difference (p = 0.049).

**Quality assessment**<br>**Number of studies**<br>**n analyzed**	**Results**
	Fassoulaki 2016 and Lin 2015 reported in text no side effects from intervention drug.<br>Ali 2018; Chowdhury 2019; Berlit 2015 reported in text reported that no major postoperative complications were observed in any group.<br>Herrador- Benito - 2024 study reported hematomas and surgical site infections, and found no significant difference.<br>Zhang 2019 reported complications such as Hydrothorax, ascites, Peritonitis, Flatulence, Venous Thrombus, and Incision Infection found no significant difference.
**Patient satisfaction**	
1 RCT<br>n = 212	1 reported mean and percentage, found no difference

*Data were reported in figures, and measures of variance were not shown in the figures.

[SIID] Gabapentinoids

Supplemental table SD.1 Gabapentinoids: Descriptive Characteristics of Studies

Author, Year<br>Country<br>Study Design<br>Ambulatory or Inpatient<br>Risk of bias	Population<br>Subgroups<br>n randomized<br>Mean age (SD)<br>Sex (% female)<br>Race/ Ethnicity<br>Mean BMI (SD)	Intervention	Comparator	Outcomes of interest reported
Pushparani, 2022<br>India<br>RCT<br>Ambulatory<br>Some concerns	Laparoscopic GI surgical patients<br>Excluded those with chronic pain and suicidal depression, inclusion of chronic opioid use NR<br>n=100<br>I: 37.92 y; C: 36.84 y<br>75% female<br>NR<br>NR	Preoperative oral gabapentin (300 mg)	Oral placebo	Pain scores at 12 hr, 24 hr<br>Opioid rescue analgesics<br>Drug associated harms
Huynh, 2021<br>USA<br>RCT<br>Inpatient<br>High	Minimally invasive Gyn surgical patients<br>Included chronic pain patients and did not conduct a subgroup analysis, inclusion of chronic opioid use and comorbid psychiatric diagnoses NR<br>n=137<br>I: 50 (12) y; C: 53 (12) y<br>100% female<br>Majority white	Preoperative oral gabapentin (600 mg)<br><br>*Also given Celecoxib and APAP preoperatively	Celecoxib and APAP preoperative	Pain scores at 24 hr, 2 weeks<br>Total opioid consumption at 24 hr, 2 weeks<br>Hospital length of stay<br>Drug associated harms<br>Gabapentin-specific harms

	I: 53% BMI <30 kg/m^2; C: 50% BMI <30 kg/m^2			
Rupniewska-Ladyko, 2018<br>Poland<br>RCT<br>Unclear<br>Low	Laparoscopic GI patients<br>Excluded those with chronic pain and comorbid psychiatric diagnoses, inclusion of chronic opioid use NR<br>n=120<br>I: 40.31 (10.89) y; C: 42.02 (10.96) y<br>I: 80.7% female; C: 80.3% female<br>NR<br>I: 42.43 (5.12) kg/m^2; C: 43.69 (5.30) kg/m^2	Preoperative oral gabapentin (1200 mg)	Oral placebo	Pain scores at 12 hr<br>Total opioid consumption at 12 hr<br>Drug associated harms

Abbreviations: BMI= body mass index; C=comparator; GI=gastrointestinal; Gyn=gynecologic; hr=hours; I= intervention; kg=kilogram; m=meters; mg=milligram; n=number of participants; NR=not reported; RCT=randomized controlled trial; SD=standard deviation; y=year(s)

Supplemental table SD.2 Gabapetninoids: Risk of Bias

**Author, Year**	**Bias from randomization process**	**Bias from deviation from intended interventions (assignment)**	**Bias due to missing outcome data**	**Bias in measurement of outcome**	**Bias in selection of reported results**	**Overall risk of bias (low, some concerns, high)**
Pushparani, 2022	Low	Low	Low	Low	Some concerns	Some concerns
Huynh, 2021	Some concerns	High	High	Some concerns	Low	High
Rupniewska-Ladyko, 2018	Low	Low	Low	Low	Low	Low

Supplemental table SD.3 Gabapentinoids: Summary of Findings

**Quality assessment**	**Results**
**Number of studies**<br>**n analyzed**	
**24 hour pain scores**<br>**VAS/NRS 0-10**	
2 RCTs<br>n=229	Pushparani 2022 – figure data only, p>0.7<br>Huynh 2021- median (IQR)= 3(4) vs 3(3), p=0.98
**24 hour cumulative opioid consumption (OMEs)**	
1 RCT<br>n=129	Huynh 2021 found no difference<br>Mean (SD)<br>I: 168 (77) OME<br>C: 161 (68) OME<br>p=0.60
**Opioid Rescue Analgesics (only for studies where cumulative opioid consumption was not reported)**	
1 RCT<br>n=100	Pushparani 2022 found benefit<br>Total tramadol consumption in number of doses (n)<br>I: 0 doses - 29, 1 dose - 17, 2 doses - 4<br>C: 0 doses - 3, 1 dose - 26, 2 doses - 21<br>p<0.001
**Hospital LOS**	

1 RCT<br>n=129	Huynh 2021 found no difference<br>Median (IQR)<br>I: 24 (19) h<br>C: 26 (21) h<br>p=0.73
**Drug-associated harms**	
3 RCT<br>n=342	Pushparani 2022 found statistically significantly less nausea and vomiting in the intervention group<br>Huynh 2021; Rupniewska-Ladyko 2018 found no difference between arms
**Gabapentin-specific harms**	
1 RCT<br>n=129	Huynh 2021 found no difference between arms.<br>Dizziness:<br>I: 17/68<br>C: 8/61<br>p=0.14<br>Somnolence:<br>I: 20/68<br>C: 23/61<br>p=0.42

Abbreviations: C=comparator; h=hours; I=intervention; IQR=interquartile range; LOS=length of stay; n=number of participants; NR=not reported; NRS=numerical rating scale; OME=oral morphine equivalent; p=p-value; RCT=randomized controlled trial; SD=standard deviation; SOE=strength of evidence; VAS=visual analogue scale

[SIIE] Intravenous dexamethasone

Supplemental table SE.1 Intravenous Dexamethasone: Descriptive Characteristics of Studies

Author, Year<br>Country<br>Study Design<br>Ambulatory or Inpatient<br>Risk of bias	Population<br>n randomized<br>Mean age (SD)<br>Sex (% female)<br>Race/ Ethnicity<br>Mean BMI (SD)	Intervention	Comparator	Outcomes of interest reported
Lee, 2018<br>South Korea<br>RCT<br>Inpatient<br>High	Patients undergoing laparoscopic cholecystectomy<br>n= 388<br>Age:<br>Younger group: 41.1 (2.4)<br>Older group: 69.2 (2)<br>% female<br>Younger group: 49.2%<br>Older group: 50.3%<br>NR<br>NR	Intravenous dexamethasone 0.1 mg/kg (5 mg/mL)	Normal saline	Pain scores (1, 6, 12, 24hrs)<br>PONV
Lee, 2017<br>South Korea<br>RCT<br>Inpatient<br>High	Patients undergoing laparoscopic Cholecystectomy<br>n=392<br>Age:<br>I: 40.3 (4.0)<br>C: 40.9 (2.4)<br>% female<br>I: 50.3%<br>C: 49.2%<br>NR<br>NR	Intravenous dexamethasone 0.1 mg/kg (5 mg/mL)	Normal saline	Pain scores (1, 6, 12, 24hrs)<br>PONV

Mohtadi, 2014<br>Iran<br>RCT<br>Inpatient<br>Low	Patients undergoing laparoscopic cholecystectomy. Those receiving opioids were excluded.<br>n= 122<br>Age:<br>I: 46.7 (5.78)<br>C: 47.26 (5.93)<br>% female<br>I: 77.0%<br>C: 75.4%<br>NR<br>NR	0.1 mg/kg (up to 8 mg) of dexamethasone	2 ml of normal saline	Pain scores (0, 2, 6, 112, 24 hrs)<br>Total opioid (24 hrs)

Supplemental table SE.2 Intravenous dexamethasone: Risk of Bias

**Author, Year**	**Bias from randomization process**	**Bias from deviation from intended interventions (assignment)**	**Bias due to missing outcome data**	**Bias in measurement of outcome**	**Bias in selection of reported results**	**Overall risk of bias (low, some concerns, high)**
Lee, 2018	Low	Low	Low	High	Low	High
Lee, 2017	Low	High	High	High	Low	High
Mohtadi, 2014	Low	Low	Low	Low	Low	Low

Supplemental table SE.3 Intravenous Dexamethasone: Summary of findings

Quality assessment	Results
**Number of studies n analyzed**	
**24-hour pain scores**	
3 RCTs<br>n= 890	Mohtadi, 2015 reported mean scores of 2.95 (SD 1.85) in the intervention group and 3.11 (SD 1.79) in the control group, with a mean difference of −0.16 (95% CI −0.81 to 0.49).<br>Lee, 2017 reported mean scores of 2.26 (SD 0.46) in the intervention group and 2.29 (SD 0.67) in the control group, with a mean difference of −0.03 (95% CI −0.15 to 0.09).<br>Lee, 2018 reported mean scores of 2.00 (SD 0.47) in the intervention group and 1.95 (SD 0.48) in the control group, with a mean difference of 0.05 (95% CI −0.04 to 0.14).
**24- hour Opioid use**	
1 RCTs<br>n= 122	Mohtadi, 2014 reported means, found a significant difference that favors intervention.<br>I: 13.28 (2.20)<br>C: 18.07 (3.17)<br>p=0.03
**Drug associated harms (PONV)**	

1 RCT<br>n= 380	Lee, 2017 reported proportion of PONV, found a significant difference that favors intervention.<br>I: 45 (23.8%)<br>C: 68 (35.6%)<br>p<0.05

[SIIF] Acetaminophen

Supplemental table SF.1 Acetaminophen: Descriptive Characteristics of Studies

Author, Year<br>Country<br>Study Design<br>Ambulatory or Inpatient<br>Risk of bias	Population<br>n randomized<br>Mean age (SD)<br>Sex (% female)<br>Race/ Ethnicity<br>Mean BMI (SD)	Intervention	Comparator	Outcomes of interest reported
Rindos, 2019<br>USA<br>RCT<br>Mixed<br>High	Laparoscopic Gyn surgery patients<br>Excluded chronic opioid use; inclusion of chronic pain and those with psychiatric diagnoses NR<br>n=183<br>I: 41.8±8.3 y<br>C: 42.1±8.2 y<br>100% female<br>NR<br>NR	1000 mg acetaminophen IV before induction of general anesthesia (preop dose) and 6 hrs after first dose (postop dose)	Normal saline	Pain scores (24 hr)<br>Additional opioid metrics<br>Adverse events<br>Patient Satisfaction
Turner, 2019<br>USA<br>RCT<br>Inpatient<br>High	Laparoscopic Gyn surgery patients<br>Inclusion of those with psychiatric diagnoses, chronic opioid use, or chronic pain NR<br>n=101<br>I:62.1±7.0 y<br>C: 60.2±8.0 y<br>100% female<br>Race/ethnicity:<br>African American: 0.9%<br>Caucasian: 98%<br>Other: 0.9%	100 mL acetaminophen IV (1000 mg/100 mL) 10-30 minutes before incision	Normal saline	Pain Scores (change in pain scores from baseline; 24 hr)<br>Total postoperative opioid consumption (24 hrs, 7 days after discharge)<br>Hospital length of stay<br>Quality of life

**Author, Year**<br>**Country**<br>**Study Design**<br>**Ambulatory or Inpatient**<br>**Risk of bias**	**Population**<br>**n randomized**<br>**Mean age (SD)**<br>**Sex (% female)**<br>**Race/ Ethnicity**<br>**Mean BMI (SD)**	**Intervention**	**Comparator**	**Outcomes of interest reported**
	I: 27.0±4.1 kg/m^2<br>C: 27.6±4.6 kg/m^2			
El Chaar, 2016<br>USA<br>RCT<br>Mixed<br>Low	Laparoscopic GI surgery patients<br>Excluded chronic pain and opioid consumption in the 7 days before surgery; inclusion of those with psychiatric diagnoses NR<br>n=100<br>I: 43.2±15.8 y<br>C: 41.1±13.9 y<br>I: 72%<br>C: 78%<br>I: 72% White, 10% Hispanic, 8% African American	IV acetaminophen (1g in 100 ml of 0.9% NS Q 6h for 24h) 30 minutes before surgery + IV acetaminophen in the 18h window post-surgery	Normal saline	Pain Scores (over 24 hrs)<br>Additional opioid metrics<br>Hospital length of stay<br>Patient Satisfaction<br>Adverse events

**Author, Year**<br>**Country**<br>**Study Design**<br>**Ambulatory or Inpatient**<br>**Risk of bias**	**Population**<br>**n randomized**<br>**Mean age (SD)**<br>**Sex (% female)**<br>**Race/ Ethnicity**<br>**Mean BMI (SD)**	**Intervention**	**Comparator**	**Outcomes of interest reported**
	C: 88% White, 12% Hispanic<br>I: 45.2±7.2 kg/m^2<br>C: 44.1±6.1 kg/m^2			

Supplemental table SF.2 Acetaminophen: Risk of Bias

**Author, Year**	**Bias from randomization process**	**Bias from deviation from intended interventions (assignment)**	**Bias due to missing outcome data**	**Bias in measurement of outcome**	**Bias in selection of reported results**	**Overall risk of bias (low, some concerns, high)**
Rindos, 2019	Low	High	High	Low	Some Concerns	High
Turner, 2019	Low	Low	High	Low	Low	High
Chaar, 2016	Low	Low	Low	Low	Low	Low

Supplemental table SF.3 Acetaminophen: Summary of Findings

**Quality assessment**	**Results**
**Number of studies**<br>**n analyzed**	
**24 hour pain scores**	
1 RCT	Rindos, 2019

n=148	Mean (SD)<br>I: 3.55 (2.47)<br>C:3.11 (2.22)<br>P-value: 0.275<br><br>1 study reported mean and found no significant difference between groups
**24 hour total opioid consumption**	
0 RCTs	-
**Additional Opioid Metrics**	
3 RCTs<br><br>n=357	No statistically significant difference between opioid consumption metrics between groups
**Hospital length of stay**	
2 RCTs<br><br>n=201	No significant difference found between groups (Turner, 2019; Chaar, 2016)
**Adverse events**	
2 RCTs<br><br>n=248	Chaar, 2016 - NS<br><br>Rindos, 2019 - NS

**Patient satisfaction**	
2 RCTs<br><br>n=248	No significant difference between groups at various time points for patient satisfaction (Rindos, 2019; Chaar, 2016)
**Quality of life**	
1 RCT<br><br>n= 77	No significant difference between groups using PROMIS-Pain interference short form - 8a (timing unclear) (Turner, 2019)

[SIIG] Nonsteroidal anti-inflammatory drugs (NSAIDs)

Supplemental table SG.1 NSAIDS vs placebo or usual care: Descriptive Characteristics of Studies

**Author, Year**<br>**Country**<br>**Study Design**<br>**Ambulatory or Inpatient**<br>**Risk of bias**	**Population**<br>**n randomized**<br>**Mean age (SD)**<br>**Sex (% female)**<br>**Race/ Ethnicity**<br>**Mean BMI (SD)**	**Intervention**	**Comparator**	**Outcomes of interest reported**
Grimsby, 2012<br>USA<br>RCT<br>Inpatient<br>Low	Laparoscopic urology surgical patients<br>Excluded chronic opioid use; inclusion of chronic pain and psychiatric diagnoses NR<br>n=135<br>I: 43 y<br>C: 43 y<br>I: 40.4% female<br>C: 61.1% female<br>Race/ethnicity NR<br>I: 25.9 kg/m^2<br>C: 26.5 kg/m^2	Ketorolac infusion starting within 30 minutes of completion of surgery	Placebo/ Saline infusion	Pain scores (24 hr)<br>Total postoperative opioid consumption (24 hr)
Lin, 2015<br>China<br>RCT<br>Inpatient<br>High	Laparoscopic GI surgery patients<br>Excluded chronic opioid use and chronic pain; inclusion of those with psychiatric diagnoses NR<br>n=120<br>I: 56.6 y<br>C: 51 y	Parecoxib IV after entering the recovery room	No postoperative analgesia unless needed	Pain scores (24 hr)<br>Additional opioid metrics<br>Hospital length of stay<br>Adverse events

**Author, Year**<br>**Country**<br>**Study Design**<br>**Ambulatory or Inpatient**<br>**Risk of bias**	**Population**<br>**n randomized**<br>**Mean age (SD)**<br>**Sex (% female)**<br>**Race/ Ethnicity**<br>**Mean BMI (SD)**	**Intervention**	**Comparator**	**Outcomes of interest reported**
	I: 70% female<br>C: 58.3% female<br>Race/ ethnicity NR<br>I: 25.0 kg/m^2<br>C: 25.1 kg/m^2			
Okam, 2024<br>Nigeria<br>RCT<br>Outpatient<br>Low	Laparoscopic Gyn surgery patients<br>Inclusion of chronic pain, chronic opioid use, and psychiatric diagnoses NR<br>n=108<br>I: 36.37 y (5.52)<br>C: 34.51 y (5.62)<br>100% female<br>Race/ ethnicity NR<br>I: 28.08 (2.61) kg/m^2<br>C: 28.27 (2.52) kg/m^2	Rectal Diclofenac given at the end of the procedure before the participant recovered from general anesthesia	Rectal Placebo	Additional opioid metrics<br>Patient satisfaction<br>Adverse events

Supplemental table SG.2 NSAIDs versus placebo or standard of care: Risk of Bias

Author, Year	Bias from randomization process	Bias from deviation from intended interventions (assignment)	Bias due to missing outcome data	Bias in measurement of outcome	Bias in selection of reported results	Overall risk of bias (low, some concerns, high)
Grimsby, 2012	Low	Low	Low	Low	Low	Low
Lin, 2015	Some Concerns	Some Concerns	Low	High	Some Concerns	High
Okam, 2024	Low	Low	Low	Low	Low	Low

Supplemental table SG.3 NSAIDs versus placebo or standard of care: Summary of Findings

**Quality assessment**	**Results**
**Number of studies n analyzed**	
**24 hour pain scores**	
2 RCTs n=231	No significant difference found between arms for both RCTs (Lin, 2015; Grimsby, 2012)
**24 hour total opioid consumption**	
1 RCT n=111	No significant difference between groups (Grimsby, 2012)
**Additional Opioid Metrics**	
2 RCTs n=228	Both RCTs measured number of participants requiring opioids. Okam, 2024 found a significant difference between groups and Lin, 2015 did not.
**Hospital length of stay**	
1 RCT n=120	No significant difference between arms (Lin, 2015)
**Patient satisfaction**	
1 RCT n=108	No significant difference between arms (Okam, 2024)
**Adverse events**	
2 RCTs n=228	Both RCTs found no significant difference in adverse events between groups (Okam, 2024; Lin, 205)

[SIIH] Other pain management interventions

Supplemental Table SH.1 Pain Management Interventions Evaluated in a Single Randomized Controlled Trial

Author	Year	Intervention	Comparator
Janjua	2019	TAP block	Intraperitoneal instillation and port site infiltration with anesthetic
Cope	2021	Ice pack in the lower abdomen	Routine care
Sherif	2016	Intrathecal morphine/NAB	Sham with saline
Chawla	2020	Mesh soaked in local anesthetic	Mesh soaked in saline
Mei	2014	Propofol plus serotonin 5-HT3 antagonist<br>Tropisetron plus serotonin 5-HT3 antagonist	Propofol plus placebo<br>Tropisetron plus placebo
Kamali	2018	Alpha-2 agonist (Dexmedetomidine)	Paracetamol
Nardone	2023	Intraperitoneal washing with isotonic saline solution at 37C	Routine care
Friesgaard	2023	Intraoperative methadone	Intraoperative morphine
Ruiz-Tovar	2020	Topical application of vitamin E on staple lines and anastomoses.	Routine care
Yao	2017	Restrictive fluid infusion	Liberal fluid infusion
Beqiri	2012	Low pressure pneumoperitoneum with and without incisional local anesthetic	Regular pressure pneumoperitoneum with and without incisional local anesthetic
Liu	2021	Nalbuphine	Placebo

Nanda	2020	Buprenorphine patch plus diclofenac	Diclofenac alone
Sherif	2017	I1: continuous infusion of dexamethasone<br>I2: continuous infusion of xylocaine	Normal saline (sham)

[SII.I] Multimodal opioid-free strategies

Supplemental Table SI.1 List of Studies Evaluating Multimodal Opioid-Free Strategies

Author	Year	Intervention	Comparator	Operation	Key Findings
Bae	2024	Dexmeditomidine and lidocaine (intraoperative)	Remifentanil (intraoperative)	Laparoscopic gastrectomy	Significantly lower opioid requirements and maximum pain scores 24 hours postoperatively in the opioid-free group versus the comparison group.<br>No significant difference in PONV, length of hospital stay, or QoR-15 score one day postoperatively between groups.
Clanet	2024	Dexmeditomidine, lidocaine, and ketamine (intraoperative)	Remifentanil and morphine (intraoperative)	Laparoscopic gastric bypass	Significantly less PONV in the opioid-free group versus the comparator group.<br>No significant difference in morphine consumption 24 hours posteroperatively, length of hospital stay, QoR40 scores, or posteroperative complications.
Collange	2024	Dexmeditomidine, lidocaine, and ketamine (intraoperative)	Sufentanil and lidocaine (intraoperative)	Laparoscopic colectomy	No significant difference in oral morphine equivalent consumption at 48 hours postoperatively, PONV, hospital length of stay, postoperative complications, or WHODAS 2.0 scores between groups.
Perez	2024	Dexmedomidine, lidocaine, and ketamine	Fentanyl (intraoperative)	Laparoscopic or robotic bariatric surgery	No significant difference at 24 hours postoperatively in total opioid consumption, pain scores, or adverse effects between groups, nor in hospital length of stay.
Kerketta	2023	Ropivacaine erector spinae plane block and lidocaine (intraoperative)	Fentanyl (intraoperative)	Upper and lower abdominal laparoscopic surgeries	Significant reduction in pain scores with movement and total analgesic requirement 24 hours postoperatively in the opioid-free group versus the comparison group.<br>No significant difference in postoperative pain scores at rest 24 hours postoperatively between groups.
Yu	2023	Esketamine, dexmedetomidine, and lidocaine with	Remifentanil with local anesthetic	Laparoscopic cholecystectomy	Significantly lower use of butorphanol for rescue analgesia within 24 hours postoperatively and pain scores up to 8 hours postoperatively (but not 24 hours) in the opioid-free

		local anesthetic incision infiltration (intraoperative)	incision infiltration (intraoperative)		group versus comparison group.<br>No significant difference in incidence of PONV.
Geng	2021	Multimodal pain regimen (preoperative oral acetaminophen and gabapentin, two intraoperative flurbiprofen doses and local anesthetic incision infiltration, then postoperative celecoxib and acetaminophen)	Conventional analgesia (single intraoperative flurbiprofen dose)	Laparoscopic gynecological surgery	Significantly decreased pain scores and total morphine consumption 24 hours postoperatively in the intervention group versus the control group.<br>No significant difference in global QoR-40 scores, PONV, or postoperative complications between groups.

[SIIJ] ERAS protocol

Supplemental table SJ.1 ERAS protocol: Descriptive Characteristics of Studies

**Author, Year**<br>**Country**<br>**Study Design**<br>**Ambulatory or Inpatient**<br>**Risk of bias**	**Population**<br>**Subgroups**<br>**n randomized**<br>**Mean age (SD)**<br>**Sex (% female)**<br>**Race/ Ethnicity**<br>**Mean BMI (SD)**	**Intervention**	**Comparator**	**Outcomes of interest reported**
Papasavas, 2023<br>USA<br>RCT<br>Ambulatory<br>High	Laparoscopic GI surgical patients<br>Excluded those with current narcotic pain medication use; chronic opioid use or psychiatric history NR<br>n=132<br>I: 38.0 (IQR, 30.5, 46.0) y; C: 39.0 (IQR, 31.0, 50.5) y<br>I: 83.1%; C: 80% female<br>I: 55.4% White; C: 58.5% White<br>I: 44.6 (IQR, 39.8, 47.0) kg/m^2; C: 42.6 (IQR, 39.1, 46.6) kg/m^2	ERAS protocol	Standard care	Pain scores at 24 hr<br>Total opioid use<br>Hospital length of stay<br>Drug associated harms
Prabhakaran, 2020<br>India<br>RCT<br>Inpatient<br>Some concerns	Laparoscopic GI surgical patients<br>H/o chronic pain, chronic opioid use, and comorbid psychiatric diagnoses NR<br>n=112<br>I: 36.21 (11.31) y; C: 36.68 (9.61) y<br>I: 75%; C: 60.7%	ERAS protocol	Standard care	Pain scores over 24 hr<br>Rescue opioid metrics<br>Hospital length of stay<br>Drug associated harms

	NR<br>I: 42.33 ± 7.01 kg/m^2; C: 45.10 ± 8.06 kg/m^2			
Ruiz-Tovar, 2019<br>Spain<br>RCT<br>Inpatien<br>Some concerns	Laparoscopic GI patients<br>H/o chronic pain, chronic opioid use, and comorbid psychiatric diagnoses NR<br>n=180<br>I: 45.3 (11.7) y; C: 44.8 (10.8) y<br>I: 72.2% female; C: 72.2% female<br>NR<br>I: 44.9 (5.5) kg/m^2; C: 44.5 (4.2) kg/m^2	ERAS protocol	Standard care	Pain scores at 24 hr<br>Total opioid consumption at 24 hr<br>Hospital length of stay<br>Drug associated harms

Abbreviations: BMI= body mass index; C=comparator; GI=gastrointestinal; Gyn=gynecologic; hr=hours; I= intervention; kg=kilogram; m=meters; mg=milligram; n=number of participants; NR=not reported; RCT=randomized controlled trial; SD=standard deviation; y=year(s)

Supplemental table SJ.2 ERAS protocol: Risk of Bias

**Author, Year**	**Bias from randomization process**	**Bias from deviation from intended interventions (assignment)**	**Bias due to missing outcome data**	**Bias in measurement of outcome**	**Bias in selection of reported results**	**Overall risk of bias (low, some concerns, high)**
Papasavas, 2023	Low	High	Low	Low	Low	High
Prabhakaran, 2020	Low	Low	Low	Some concerns	Low	Some concerns
Ruiz-Tovar, 2019	Low	Low	Low	Some concerns	Low	Some concerns

Supplemental table SJ.3 ERAS protocol: Summary of Findings

Quality assessment<br>Number of studies<br>n analyzed	Results
**24 hour pain scores VAS/NRS 0-10**	
2 RCTs<br>n=292	Ruiz-Tovar 2019; Mean (SD)<br>I: 1.6 (1.2); C: 37 (2.8)<br>p<0.001<br>Prabhakaran 2020; Mean (SD)<br>I: 0.43 (0.50); C: 0.57 (0.51)<br>p>0.05
**Total cumulative opioid consumption (OMEs)**	
1 RCT<br>n=130	Papasavas 2023; Median (IQR)<br>I: 27.2 (9.4, 47.5) MME<br>C: 39.2 (19.2, 73.2) MME<br>p=0.011
**Opioid Rescue Analgesics (only for studies where cumulative opioid consumption was not reported)**	
1 RCT<br>n=180	Ruiz-Tovar 2019; % needing morphine rescue<br>I: 2.2%; C: 11.1%<br>p= 0.009
1 RCT<br>n=112	Prabhakaran 2020; time to first rescue; Mean (SD)<br>I: 5.57 (1.23) hr; C: 3.10 (0.99) hr<br>p <0.001
**Hospital LOS**	
3 RCTs<br>n = 422	Ruiz-Tovar 2019; Mean (SD)<br>I: 2.8 (3.1) days; C: 1.7 (1.8) days<br>p<0.001<br>Prabhakaran 2020; Mean (SD)<br>I: 1.36 (0.56) days; C: 1.89 (0.73) days

	p=0.003<br>Papasavas 2023,<br>Difference of 0.48 days (95% 0.13, 0.83)<br>p= 0.008
**Adverse events**	
3 RCT<br>n=422	2 RCTs reported no significant differences<br>1 RCT favored intervention<br>Ruiz-Tovar 2019, %<br>I: 8.8%; C: 2.2%; p<0.05<br><br>3 RCTs reported no difference in complication rates

Abbreviations: C=comparator; h=hours; I=intervention; IQR=interquartile range; LOS=length of stay; n=number of participants; NR=not reported; NRS=numerical rating scale; OME=oral morphine equivalent; p=p-value; RCT=randomized controlled trial; SD=standard deviation; SOE=strength of evidence; VAS=visual analogue scale

[SIIK] Active CO2 Removal

Supplemental table SK.1 Active CO2 Removal: Descriptive Characteristics of Studies

Author, Year<br>Country<br>Study Design<br>Ambulatory or Inpatient<br>Risk of bias	Population<br>n randomized<br>Mean age (SD)<br>Sex (% female)<br>Race/ Ethnicity<br>Mean BMI (SD)	Intervention	Comparator	Outcomes of interest reported
Arumugaswamy, 2024<br>India<br>RCT<br>Inpatient<br>Some concerns	Patients undergoing laparoscopic cholecystectomy<br>n = 181<br>Age:<br>I:38.6 (11.9)<br>C: 41.9 (14.1)<br>% female<br>I:78.9%<br>C: 73.6%<br>NR<br>BMI<br>I:24.7 (4.5)<br>C: 25.6 (3.8)	Low pressure insufflation 8-10 mmHg CO2	Standard pressure insufflation 12-14mmHg CO2	Pain scores (24 hrs, 7 days)<br>Total opioid<br>Length of hospital stay<br>PONV<br>PO complications

**Author, Year**<br>**Country**<br>**Study Design**<br>**Ambulatory or Inpatient**<br>**Risk of bias**	**Population**<br>**n randomized**<br>**Mean age (SD)**<br>**Sex (% female)**<br>**Race/ Ethnicity**<br>**Mean BMI (SD)**	**Intervention**	**Comparator**	**Outcomes of interest reported**
Noh, 2024<br>South Korea<br>RCT<br>NR<br>Low	Patients undergoing laparoscopic cholecystectomy<br>n= 110<br>Age:<br>I: 49 (11)<br>C:47 (13)<br>% female<br>I: 59.6%<br>C: 50%<br>NR<br>BMI<br>I:25 (4)<br>C: 25(4)	Lung-recruitment maneuver	Control	Pain scores (24 hrs)<br>Total opioid (24 hrs)<br>Patient satisfaction<br>PONV<br>PO complications
Park, 2023<br>South Korea<br>RCT<br>Inpatient<br>Low	Patients undergoing laparoscopic cholecystectomy<br>n= 212<br>Age:<br>I:45.9 (11.3)<br>C: 46.6 (13.1)<br>% female<br>I: 63.7%<br>C:63.7%<br>NR<br>BMI<br>I: 23.5 (3.6)<br>C: 24.3 (3.8)	Low pressure insufflation 4mmHg CO_2	Standard pressure insufflation 12mmHg CO_2	Pain scores (24, 48 hrs)<br>Total opioid (48 hrs)<br>Length of hospital stay<br>PONV

**Author, Year<br>Country<br>Study Design<br>Ambulatory or Inpatient<br>Risk of bias**	**Population<br>n randomized<br>Mean age (SD)<br>Sex (% female)<br>Race/ Ethnicity<br>Mean BMI (SD)**	**Intervention**	**Comparator**	**Outcomes of interest reported**
Abuelzein, 2023<br>Egypt, Saudi Arabia<br>RCT<br>Inpatient<br>Some concerns	Patients undergoing laparoscopic cholecystectomy<br>n= 816<br>Age:<br>I: 36.53 (10.32)<br>C:38.26 (12.34)<br>% female<br>I:84.6 %<br>C:87.32 %<br>NR<br>BMI<br>I:27.56 (2.32)<br>C:27.43 (2.17)	Active intraabdominal C02 removal	Standard of care	Pain scores (24 hrs)<br>Length of hospital stay
Pasquier, 2021<br>Sweden<br>RCT<br>Inpatient<br>High	Patients undergoing laparoscopic cholecystectomy<br>n= 283<br>Age:<br>Median(IQR)-<br>I:44.5 (36.3–58.8)<br>C:46.0 (37.0–57.0)<br>% female<br>I:56 (73.7%)<br>C:56 (78.9%)<br>NR<br>BMI<br>Median(IQR)<br>I: 27.2 (24.6–31.8)<br>C: 27.4 (24.0–30.9)	Pulmonary recruitment maneuver	Standard of care	Pain scores (24, 36, 48 hrs)<br>Length of hospital stay<br>PONV<br>PO complications

**Author, Year**<br>**Country**<br>**Study Design**<br>**Ambulatory or Inpatient**<br>**Risk of bias**	**Population**<br>**n randomized**<br>**Mean age (SD)**<br>**Sex (% female)**<br>**Race/ Ethnicity**<br>**Mean BMI (SD)**	**Intervention**	**Comparator**	**Outcomes of interest reported**
Chalermchockcharoenkit, 2021<br>Thailand<br>RCT<br>Inpatient<br>Low	Patients undergoing gynecologic laparoscopic surgery<br>n= 220<br>Age:<br>I: 43 (10.66)<br>C:43 (7.71)<br>% female<br>100%<br>NR<br>BMI<br>I: 22.74 (4.69)<br>C:22.05 (3.26)	Delayed suprapubic port removal	Standard of care	Pain scores (12-24, 24-48 hrs)<br>Opioid rescue analgesia
Radosa, 2019<br>Germany<br>RCT<br>Inpatient<br>High	Patients undergoing laparoscopic hysterectomy<br>n= 221<br>Age: median (IQR)<br>I: 51 (40- 79)<br>C: 49 (39- 78)<br>% female<br>100%<br>NR<br>BMI Median(IQR)<br>I: 26 (21.6–35.9)<br>C: 27.8 (19–29.2)	Low pressure insufflation with 8mmHg CO_2	Standard pressure insufflation with15mmHg CO_2	Pain scores (24, 48 hrs)<br>Length of hospital stay<br>PONV<br>PO complications<br>Total opioid (piritramide) 24 hrs

Author, Year<br>Country<br>Study Design<br>Ambulatory or Inpatient<br>Risk of bias	Population<br>n randomized<br>Mean age (SD)<br>Sex (% female)<br>Race/ Ethnicity<br>Mean BMI (SD)	Intervention	Comparator	Outcomes of interest reported
Pasquier, 2018<br>Sweden<br>RCT<br>Inpatient<br>High	Patients undergoing laparoscopic bariatric surgery<br>n= 200<br>Age: Median (IQR)<br>I:47 (34–53)<br>C:44 (32–51)<br>% female<br>100%<br>NR<br>BMI Median(IQR)<br>I: 38 (36–42)<br>C:38 (36–42)	Pulmonary recruitment maneuver	standard care	Pain scores (24, 36, 48 hrs)<br>Total opioid (48 hrs)<br>Length of hospital stay<br>PONV<br>IO complications
Gungorduk, 2018<br>Turkey<br>RCT<br>Inpatient<br>Low	Patients undergoing laparoscopic gynecological oncologic surgery<br>n= 146<br>Age:<br>I:49.3 (8.1)<br>C:48.6 (7.5)<br>% female<br>100%<br>NR<br>BMI<br>I:26.9 (2.1)<br>C:26.5 (2.4)	Pulmonary recruitment maneuver	Standard of care	Pain scores (24, 48 hrs)<br>Length of hospital stay<br>PONV<br>PO complications<br>Opioid rescue (12 hrs)

**Author, Year<br>Country<br>Study Design<br>Ambulatory or Inpatient<br>Risk of bias**	**Population<br>n randomized<br>Mean age (SD)<br>Sex (% female)<br>Race/ Ethnicity<br>Mean BMI (SD)**	**Intervention**	**Comparator**	**Outcomes of interest reported**
Asgari, 2018<br>Iran<br>RCT<br>Inpatient<br>Some concerns	Patients undergoing gynecologic laparoscopic surgery<br>n= 168<br>Age:<br>I: 36.53 (8.18)<br>C: 36.21 (8.25)<br>% female<br>100%<br>NR<br>BMI<br>I: 26.55 (5.09)<br>C: 25.28 (3.83)	Drain	No drain	Pain scores (24 hrs)<br>Total opioid (24 hrs)<br>Length of hospital stay
Singla, 2014<br>India<br>RCT<br>Inpatient<br>Some concerns	Patients undergoing laparoscopic cholecystectomy<br>n=100<br>Age:<br>I: 50.6 (13.95)<br>C: 53.76 (13.80)<br>% female<br>I:78.9%<br>C: 73.6%<br>NR<br>BMI<br>I:24.7 (4.5)<br>C: 25.6 (3.8)	Low pressure insufflation 7-8mmHg CO_2	Standard pressure insufflation 12-14mmHg CO_2	Pain scores (24 hrs)<br>PONV<br>IO complications<br>Opioid rescue<br>Total fentanyl by interval time

Author, Year<br>Country<br>Study Design<br>Ambulatory or Inpatient<br>Risk of bias	Population<br>n randomized<br>Mean age (SD)<br>Sex (% female)<br>Race/ Ethnicity<br>Mean BMI (SD)	Intervention	Comparator	Outcomes of interest reported
Tsai, 2013<br>Taiwan<br>RCT<br>Inpatient<br>Low	Patients undergoing gynecologic laparoscopic surgery<br>n= 102<br>Age:<br>I: 39.76(9.04)<br>C: 38.96(8.46)<br>% female<br>100%<br>NR<br>BMI<br>I: 22.76(3.94)<br>C: 22.66(3.98)	Pulmonary recruitment maneuver and intraperitoneal normal saline infusion	Standard of care	Pain scores (24, 48 hrs)<br>Total opioid (48 hrs)<br>Length of hospital stay<br>PONV<br>PO complications
Radosa, 2013<br>Germany<br>RCT<br>Inpatient<br>Some concerns	Patients undergoing laparoscopic hysterectomy<br>n= 293<br>Age:<br>I: 45.74 (8.35)<br>C:45.21 (6.38)<br>% female<br>100%<br>NR<br>BMI<br>I: 27.14 (6.11)<br>C: 26.18 (5.82)	Extended assisted ventilation with an open umbilical trocar valve	Standard of care	Pain scores (24, 48 hrs)<br>Length of hospital stay<br>PONV<br>PO complications<br>Total opioid (piritramide) 24 hrs

Supplemental table SK.2 Active CO2 Removal: Risk of Bias

Author, Year	Bias from randomization process	Bias from deviation from intended interventions (assignment)	Bias due to missing outcome data	Bias in measurement of outcome	Bias in selection of reported results	Overall risk of bias (low, some concerns, high)
Arumugaswamy, 2024	Low	Some concerns	Low	Low	Low	Some concerns
Noh, 2024[80]	Low	Low	Low	Low	Low	Low
Park, 2023	Low	Low	Low	Low	Low	Low
Abuelzein, 2023	Some concerns	Low	Low	Low	Low	Some concerns
Pasquier, 2021	Low	High	Some concerns	Low	Low	High
Chalermchockcharoenkit, 2021	Low	Low	Low	Low	Low	Low
Radosa, 2019	Low	High	High	Low	Low	High
Pasquier, 2018	Low	High	Some concerns	Low	Low	High
Gungorduk, 2018	Low	Low	Low	Low	Low	Low
Asgari, 2018	Low	Some concerns	Low	Some concerns	Low	Some concerns
Singla, 2014	Some concerns	Some concerns	Low	Low	Low	Some concerns
Tsai, 2013	Low	Low	Low	Low	Low	Low
Radosa, 2013	Some concerns	Low	Low	Some concerns	Low	Some concerns

Supplemental table SK.3 Active CO2 Removal: Summary of Findings

Quality assessment<br>Number of studies<br>n analyzed	Results
**24-hour pain scores**	
12 RCTs<br>n= 2368	Park, 2023 reported a mean score of 2.6 (SD 0.6) in the intervention group and 3.2 (SD 1.0) in the control group.<br>Singla, 2014 reported a mean score of 0.08 (SD 0.274) in the intervention group and 1.00 (SD 1.565) in the control group.<br>Arumugaswamy, 2024 reported a mean score of 2.1 (SD 1.4) in the intervention group and 2.1 (SD 1.2) in the control group.<br>Abuelzein, 2023 reported median scores of 1.5 (IQR 0–2) in the intervention group and 2.0 (IQR 0–2) in the control group; standard deviations were not reported.<br>Pasquier, 2018 reported median scores of 2 (IQR 1–3) in the intervention group and 3 (IQR 2–5) in the control group; standard deviations were not reported.<br>Pasquier, 2021 reported median scores of 2 (IQR 1–3) in the intervention group and 3 (IQR 1–4) in the control group; standard deviations were not reported.<br>Gungorduk, 2018 reported mean scores of 4.7 (SD 0.5) in the intervention group and 4.8 (SD 0.3) in the control group.<br>Asgari, 2018 reported mean scores of 3.86 (SD 1.57) in the intervention group and 3.51 (SD 1.27) in the control group.<br>Tsai, 2013 reported mean scores of 5.67 (SD 2.26) in the intervention group and 6.12 (SD 1.91) in the control group.<br>Radosa, 2013 reported mean scores of 4.28 (SD 1.51) in the intervention group and 5.14 (SD 1.49) in the control group.<br>Noh, 2024 reported median scores of 2 (IQR 1–2) in the intervention group and 3 (IQR 2–3) in the control group; standard deviations were not reported.<br>Radosa, 2019 reported median pain scores of 1 (IQR 0–8) in the intervention group and 0 (IQR 0–8) in the control group, with a statistically significant difference ($p \leq 0.01$).
**48-hour pain scores**	
7 RCTs<br>n= 1057	Park, 2023 reported mean scores of 2.6 (SD 0.6) in the intervention group and 2.8 (SD 0.6) in the control group.

	Arumugaswamy, 2024 reported mean scores of 0.6 (SD 0.9) in the intervention group and 0.5 (SD 0.8) in the control group.<br>Pasquier, 2018 reported *median* scores of 1 (IQR 0–2) in the intervention group and 1 (IQR 0–2.75) in the control group; standard deviations were not reported.<br>Pasquier, 2021 reported *median* scores of 1 (IQR 0–3) in the intervention group and 2 (IQR 1–3) in the control group; standard deviations were not reported.<br>Gungorduk, 2018 reported mean scores of 2.9 (SD 0.5) in the intervention group and 3.1 (SD 0.5) in the control group.<br>Tsai, 2013 reported mean scores of 3.41 (SD 2.33) in the intervention group and 4.18 (SD 2.55) in the control group.<br>Radosa, 2013 reported mean scores of 3.64 (SD 1.66) in the intervention group and 4.22 (SD 1.43) in the control group.
**24- hour Opioid use**	
3 RCTs<br>n= 492	2 RCTs (Radosa 2013; Asgari, 2018) reported means:<br>Radosa 2013 favors intervention<br>Radosa 2019 reported a median: favors intervention
**48- hour Opioid use**	
3 RCTs<br>n= 432	Park, 2023 reported mean opioid consumption of 34.50 OME (SD 25.38) in the intervention group and 39.90 OME (SD 35.31) in the control group, with a mean difference of −5.40 OME (95% CI −14.33 to 3.53).<br>Pasquier, 2018 reported mean opioid consumption of 17.12 OME (SD 18.12) in the intervention group and 29.12 OME (SD 22.70) in the control group, with a mean difference of −12.00 OME (95% CI −18.62 to −5.38).<br>Tsai, 2013 reported mean opioid consumption of 31.26 OME (SD 12.96) in the intervention group and 34.56 OME (SD 13.12) in the control group, with a mean difference of −3.30 OME (95% CI −8.41 to 1.81).
**Total opioid use at discharge**	

1 RCT<br>n= 181	Arumugaswamy_2024: no significant difference
**24- hour Opioid rescue use (for those that did not report total opioid)**	
3 RCTs<br>n= 457	3 RCTs (Chalermchockcharoenkit_2021; Pasquier_2021; Singla_2014) reported rescue proportion, one found a significant difference favoring the intervention.
**Length of stay**	
10 RCTs<br>n= 2162	Arumugaswamy, 2024 reported a mean hospital stay of 2.10 days (SD 0.40) in the intervention group and 2.14 days (SD 0.30) in the control group, with a mean difference of −0.04 days (95% CI −0.14 to 0.06).<br>Abuelzein, 2023 reported mean stays of 1.40 days (SD 0.33) in the intervention group and 1.74 days (SD 0.54) in the control group, with a mean difference of −0.34 days (95% CI −0.40 to −0.28).<br>Park, 2023 reported mean stays of 2.00 days (SD 0.10) in both intervention and control groups, resulting in a mean difference of 0.00 days (95% CI −0.06 to 0.06).<br>Pasquier, 2021 reported identical mean stays of 1.00 day (SD 0.00) in both groups, with a mean difference of 0.00 days (95% CI 0.00 to 0.00).<br>Pasquier, 2018 similarly reported mean stays of 1.00 day (SD 0.00) in both groups, with a mean difference of 0.00 days (95% CI 0.00 to 0.00).<br>Gungorduk, 2018 reported mean stays of 2.90 days (SD 1.00) in the intervention group and 2.81 days (SD 1.00) in the control group, with a mean difference of 0.10 days (95% CI −0.32 to 0.52).<br>Asgari, 2018 reported mean stays of 1.21 days (SD 0.58) in the intervention group and 1.40 days (SD 0.76) in the control group, with a mean difference of −0.19 days (95% CI −0.43 to 0.05).

	Tsai, 2013 reported mean stays of 2.64 days (SD 0.88) in the intervention group and 2.98 days (SD 0.89) in the control group, with a mean difference of −0.34 days (95% CI −0.69 to 0.01).<br>Radosa, 2013 reported mean stays of 5.38 days (SD 1.21) in the intervention group and 5.93 days (SD 1.72) in the control group, with a mean difference of −0.55 days (95% CI −0.97 to −0.13).<br>Radosa, 2019 reported median, found a significant difference, was not included in MA because data was skewed.
**Patient satisfaction**	
1 RCT<br>n = 106	Noh 2024 reported median score and found a significant difference favoring intervention
**Drug associated harms (PONV)**	
8 RCTs<br>n= 1081	8 RCTs (Pasquier, 2021; Radosa, 2019; Pasquier, 2018; Gungorduk, 2018; Singla, 2014; Tsai, 2013; Radosa, 2013; Noh 2024) reported proportion of participants with nausea, vomiting or both, among which Radosa 2019 found a significant difference favoring intervention; and others reported no significant difference.
**Intervention associated harms (IO complications)**	
2 RCTs<br>n = 278	2 RCTs (Radosa 2019; Singla 2014) reported the proportions of participants who suffered intraoperative complications (bile leak, bladder injury, bleeding), none found a significant difference.

[SIIL] Warm humidified insufflation

Supplemental table SL.1 Warm humidified insufflation: Descriptive Characteristics of Studies

Author, Year<br>Country<br>Study Design<br>Ambulatory or Inpatient<br>Risk of bias	Population<br>n randomized<br>Mean age (SD)<br>Sex (% female)<br>Race/ Ethnicity<br>Mean BMI (SD)	Intervention	Comparator	Outcomes of interest reported
Jiang, 2019<br>China<br>RCT<br>Inpatient<br>Some concerns	Elderly patients undergoing laparoscopic colorectal surgery. Excluded participants with a history of alcohol or drug abuse<br>n =50<br>Age:<br>I1: 69.06 (3.65)<br>C1: 68.52 (3.11)<br>C2: 68.37 (4.05)<br>% female<br>I1 : 24%<br>C1: 30%<br>C2: 36%<br>NR<br>BMI:<br>I1: 23.18 (2.88)<br>C1:23.06 (3.16)<br>C2: 22.84 (3.81)	I1(WH): warm [37°C], humidified<br>[98% relative humidity] CO2 insufflation	C1(CF): 20°C, 0% relative humidity CO2 insufflation, intraoperative warming with forced-air warmer set to 38°C<br>C2(CE): 20°C, 0% relative humidity CO2 insufflation, intraoperative warming with electric blankets set to 38°C	Pain scores (24, 48hrs)<br>Patient satisfaction<br>Length of hospital stay<br>Adverse events<br>Sufentanil consumption (12-24, 24-48hrs)

Sutton, 2017<br>USA<br>RCT<br>Inpatient<br>Low	Patients undergoing minimally invasive colon resection<br>n=104<br>Age:<br>Median (IQ range)<br>I: 60.5 (24.5)<br>C: 56 (17)<br>% female<br>I: 48%<br>C: 60.8%<br>NR<br>BMI:<br>I: 26.08(4.51)<br>C: 27.26(4.49)	Warm and humidified CO2 Pneumoperitoneum (36.7°C, 95% humidity)	Cold and dry CO2 Pneumoperitoneum (room temperature, 0% humidity)	Pain scores (POD1-POD7)<br>Total opioid (POD1- POD 7)<br>Length of hospital stay<br>PO complications
Klugsberger, 2014<br>Austria<br>RCT<br>Inpatient<br>Low	Patients undergoing laparoscopic cholecystectomy<br>n=143<br>Age:<br>I: 55.68 (16.93)<br>C: 55.87 (14.44)<br>% female<br>I: 70.4%<br>C: 68.7%<br>NR<br>BMI:<br>I: 28.74(5.40)<br>C: 28.34(5.22)	Humidified CO2	Standard CO2 insufflation	Pain scores POD 1<br>Rescue piritramide

Supplemental table SL.2 Warm humidified insufflation: Risk of Bias

Author, Year	Bias from randomization process	Bias from deviation from intended interventions (assignment)	Bias due to missing outcome data	Bias in measurement of outcome	Bias in selection of reported results	Overall risk of bias (low, some concerns, high)
Jiang, 2019	Some concerns	Low	Low	Some concerns	Low	Some concerns

Sutton, 2017	Low	Low	Low	Low	Low	Low
Klugsberger, 2014	Low	Low	Low	Low	Low	Low

Supplemental table SL.3 Warm humidified insufflation: Summary of findings

Quality assessment<br>Number of studies<br>n analyzed	Results
**24-hour pain scores**	
3 RCTs<br>n= 399	Two RCTs (Jiang, 2019; Sutton, 2017) reported median pain scores, none found significant differences.<br>One RCT (Klugsberger, 2014) reported mean pain scores and found no significant differences.
**48-hour pain scores**	
2 RCTs<br>n= 251	Two RCTs (Jiang, 2019; Sutton, 2017) reported median pain scores, none found significant differences.
**24- hour Opioid use**	
2 RCTs<br>n= 101	Sutton, 2017 reported morphine equivalent daily dose (MEDD) and found a significant difference that favors intervention.<br>Median (IQR)<br>I: 23 (41.71)<br>C: 48.6 (51.5)<br>p= 0.009<br>Klugsberger, 2014 reported no significant difference.<br>Mean (SD-NR)<br>I: 277.5 μg<br>C: 90 μg<br>p= 0.751
**Length of stay**	
2 RCTs<br>n= 251	Two RCTs reported median length of stay, one found a significant difference that favors the intervention.<br>Jiang, 2019:<br>Median days (IQR)<br>I: 8.7 (6.3–12.5)<br>C1: 9.5 (7.1–11.9)<br>C2: 12.2 (7.5–13.2)<br>p=0.048<br>I vs C1 p >0.05

	I vs C2 p <0.05<br><br>Sutton 2017- no significant difference
**Patient satisfaction**	
1 RCT<br>n = 106	One RCT (Jiang, 2019) reported median satisfaction score and found a significant difference that favors the intervention vs one of the control groups.<br>Median (IQR)<br>I: 4.25 (3.25–4.75)<br>C1: 4.25 (3.00–4.75)<br>C2: 3.75 (2.75–4.75)<br>p=0.012<br>I vs C1 p >0.05<br>I vs C2 p <0.05
**Adverse events**	
3 RCTs<br>n= 399	All three RCTs report no significant differences in adverse event rates (nausea/vomiting, superficial or deep surgical site infection, anastomotic leak or intraabdominal abscess, urinary retention, cardiac or pulmonary complications, and small bowel obstruction, arrhythmias, hypertension, or hypotension).

[SIIM] Complementary and alternative medicine strategies

Supplemental Table SM.1 List of Studies Evaluating Complementary and Alternative Medicine Strategies

Author	Year	Intervention	Comparator	Operation	Key Findings
Zeeni	2020	Trendelenburg position (20 °) once fully awake and cooperative in the PACU and retained this position for the first 24 h	Standard care	Gynecological laparoscopic procedures	NRS pain scores [Median (IQR)] were significantly lower in the intervention group at 12 h compared to the control group (0 [0–1] versus 5 [1–4], p < 0.001), furthermore improvement in postoperative shoulder pain between time of arrival to PACU (time zero) and 12 h postoperatively was significantly higher in patients allocated to the experimental group compared to the control group. Pain scores were significantly lower in patients allocated to the experimental group versus the control group (0 [0–1] versus 5 [1–4], p < 0.001)
Gu	2019	Long duration transcutaneous electrical acupoint stimulation (L-TEAS)	Placebo (C-TEAS)	Laparoscopic radical gastrectomy	Compared to the C-TEAS group, the VAS scores at 4 h (2.79 ± 0.48 vs. 3.41 ± 0.62), 8 h (2.65 ± 0.42 vs. 3.17 ± 0.49), and 24 h (1.98 ± 0.39 vs. 2.72 ± 0.73) were significantly decreased in the L-TEAS group (P < 0.05) after the operation. Compared to the C-TEAS group, analgesic consumption at 4 h (10.02 ± 2.26 vs. 13.38 ± 2.98), 8 h (20.55 ± 4.59 vs. 24.63 ± 3.94) and 36 h (72.64 ± 9.74 vs. 76.01 ± 10.43) was significantly decreased in the L-TEAS group (P < 0.05) after the operation. There was a significant difference in the incidence of PONV between the L-TEAS (12%) and C-TEAS (32.2%) groups (P < 0.05). No significant difference was observed with respect to RSS among the 2 groups (P > 0.05). The time intervals from surgery to the first bowel sounds as well as the passage of flatus and defecation were shorter in the L-TEAS group than in the C-TEAS group (19.69 ± 7.61 h vs. 26.58 ± 7.43 h, 36.58 ± 10.75 h vs. 43.56 ± 9.57 h and 71.48 ± 20.62 h vs. 77.27 ± 22.67 h, respectively) (P < 0.05). The rate of moderately satisfaction and above in the L-TEAS (67.3%) group was higher than for that in the C-TEAS group (42.4%), and for very satisfied, the LTEAS (55.2%) group was higher than that in the C-TEAS group (15.3%) (P < 0.05).

Joudi	2016	Hypnosis	Standard care	Laparoscopic cholecystectomy	Results suggest that hypnosis could effectively reduce pain after laparoscopic cholecystectomy and significantly reduce hospitalization time
Peng	2023	Rehabilitation exercise and pain neuroscience education	Standard care	Laparoscopic colorectal surgery	Compared with those receiving conventional analgesia (group CA, N=89), patients in group REPNE (N=86) reported reduced intensity of pain 24 h after surgery, less risk of pain catastrophizing and sensitization, and better quality of life during hospitalization recovery till 1 month after surgery ($p<0.05$). No statistical diference was found for neuropathic transformation of post-operative pain or for the incidence of chronic post-operative pain ($p>0.05$).
Artiklar	2024	I1: Breathing and coughing exercises<br><br>I2: Breathing and coughing exercises + 2 liters per minute O_2 treatment	Standard care	Laparoscopic cholecystectomy	After surgery, a statistically significant difference was found in favor of the intervention groups in pain levels at the 12th hour ($P < .05$). There was a negative and statistically significant difference between pain levels and SpO_2 values in all groups ($P < .05$). Non-opioids were consumed in lower amounts in the intervention groups ($P > .05$), while opioids were consumed only in the control group.
Kheradkish	2024	Acupressure. Selected pressure points were LI11, LI4, SJ5, HT7, P6, and K1.	Sham (thumb pad pressed)	Laparoscopic cholecystectomy	The average VAS significantly decreased in both the intervention and control groups ($p < 0.001$). The intervention group had a significantly higher reduced score compared to the control group, 30 min and 72 h after applying acupressure ($p < 0.001$). Significant differences were observed between the two groups in terms of changes in systolic blood pressure (SBP), diastolic blood pressure (DBP), heart rate (HR), and breath rate (BR) ($p < 0.05$).
Wang	2024	Preoperative virtual reality video (soothing; natural)	Standard care	Gynecological laparoscopic procedures	The VAS scores [Median (IQR)] at 30 min [2 (1,2) vs. 3 (2,3)], 2 h [2 (2,3) vs. 4 (3,4)], 4 h [3 (2,4) vs. 4 (4,5)], 8 h [3 (2,4) vs. 4 (4,5)], 12 h [2 (2,3) vs. 4 (3,4)], 24 h [1 (1,2) vs. 3 (2,3)] after surgery. Generalized estimation equation (GEE) indicated that VR intervention was negatively correlated with postoperative VAS values ($\beta = -0.830$, S.E = 0.199, 95%CI (− 1.220,-0.439), Wald $\chi 2 = 17.359$, $p<0.05$), in the meanwhile, VR also lower the incidence

					of moderate pain (VAS > 4) at 8 h postoperatively (12.1% vs 31.0%, $p = 0.013$). However, the 24 h tramadol usage remained unchanged. Patients in the VR group had better sleep quality (6.33 ± 2.3 vs. 4.12 ± 2.5, $p < 0.001$) and lower incidence of nausea (43.1% vs. 63.2%, $p < 0.05$), dizziness (0% vs. 14.0%, $p < 0.05$), and headache (12.1% vs. 29.8%, $p < 0.05$).

[SIII] Alphabetized List of Included Studies

1. Abet E, Orion F, Denimal F, et al. Interest of Using Ropivacaine for Outpatient Laparoscopic Cholecystectomy: Prospective Randomized Trial. World J Surg. 2017 Mar;41(3):687-92. doi: 10.1007/s00268-016-3797-2. PMID: 27872974.
2. Abuelzein MLA, Baghdadi MA, Abbdelhady WA, et al. A prospective randomized controlled study on the role of restoring liver diaphragm surface tension and pain control at port sites in optimizing pain management following laparoscopic cholecystectomy. Annals of Gastroenterological Surgery. 2023 Jan;7(1):131-7. doi: 10.1002/ags3.12602. PMID: 36643366.
3. Aldohayan A, Alshammari S, Binjaloud A, et al. Pre-incisional Laparoscopic Preperitoneal Local Anesthetic Technique in Laparoscopic Sleeve Gastrectomy. Journal of the Society of Laparoendoscopic Surgeons. 2022;26(3):Jul-Sep. doi: 10.4293/jsls.2022.00049. PMID: 36071990.
4. Ali S, Zarin M, Jan Z, et al. Effect of bupivacaine on postoperative pain after laparoscopic cholecystectomy. Journal of the College of Physicians and Surgeons Pakistan. 2018 Sep;28(9):663-6. doi: 10.29271/jcpsp.2018.09.663. PMID: 30158029.
5. Arden D, Seifert E, Donnellan N, et al. Intraperitoneal Instillation of Bupivacaine for Reduction of Postoperative Pain After Laparoscopic Hysterectomy: A Double-Blind Randomized Controlled Trial. J Minim Invasive Gynecol. 2013;20(5):620-6. doi: 10.1016/j.jmig.2013.03.012. PMID: 52594629.
6. Artiklar T, Erden S. The Effect of Breathing and Coughing Exercises and Oxygen Therapy on Shoulder Pain and Analgesic Consumption After Laparoscopic Cholecystectomy: A Randomized Controlled Study. Journal of perianesthesia nursing : official journal of the American Society of PeriAnesthesia Nurses. 2024 Aug;39(4):540-6. doi: 10.1016/j.jopan.2023.10.008. PMID: 38069969.
7. Arumugaswamy PR, Chumber S, Rathore YS, et al. Low-pressure pneumoperitoneum with deep neuromuscular blockade versus standard pressure pneumoperitoneum in patients undergoing laparoscopic cholecystectomy for gallstone disease: a non-inferiority randomized control trial. Surg Endosc. 2024 Jan;38(1):449-59. doi: 10.1007/s00464-023-10558-0. PMID: 38012441.
8. Asgari Z, Hosseini R, Rastad H, et al. Does peritoneal suction drainage reduce pain after gynecologic laparoscopy? Surgical Laparoscopy, Endoscopy and Percutaneous Techniques. 2018 Apr;28(2):73-6. doi: 10.1097/sle.0000000000000490. PMID: 29189662.
9. Bae MI, Oh J, Lee HS, et al. Influence of psychological factors and pain sensitivity on the efficacy of opioid-free anesthesia: A randomized clinical trial. Gen Hosp Psychiatry. 2024 Jul-Aug;89:84-92. doi: 10.1016/j.genhosppsych.2024.04.001. PMID: 38838608.
10. Barr Grzesh RL, Treszezamsky AD, Fenske SS, et al. Use of Paracervical Block Before Laparoscopic Supracervical Hysterectomy. Journal of the Society of Laparoendoscopic Surgeons. 2018;22(3):Jul-Sep. doi: 10.4293/jsls.2018.00023. PMID: 30356343.
11. Beqiri AI, Domi RQ, Sula HH, et al. The combination of infiltrative bupivacaine with low-pressure laparoscopy reduces postcholecystectomy pain. A prospective randomized controlled study. Saudi Med J. 2012;33(2):134-8. PMID: 22327752.

12. Berlit S, Tuschy B, Brade J, et al. Topical anaesthetic patches for postoperative wound pain in laparoscopic gynaecological surgery: a prospective, blinded and randomised trial. Arch Gynecol Obstet. 2015 Mar;291(3):585-90. doi: 10.1007/s00404-014-3462-7. PMID: 25216962.
13. Calle GA, Lopez CC, Sanchez E, et al. Transversus abdominis plane block after ambulatory total laparoscopic hysterectomy: Randomized controlled trial. Acta Obstet Gynecol Scand. 2014;93(4):345-50. doi: 10.1111/aogs.12351. PMID: 372699231.
14. Cataldo R, Bruni V, Migliorelli S, et al. Laparoscopic-Guided Transversus Abdominis Plane (TAP) Block Combined with Port-Site Infiltration (PSI) for Laparoscopic Sleeve Gastrectomy in an ERABS Pathway: A Randomized, Prospective, Double-Blind, Placebo-Controlled Trial. Obes Surg. 2024 Jul;34(7):2475-82. doi: 10.1007/s11695-024-07292-4. PMID: 38764003.
15. Chalermchockcharoenkit A, Hengrasmee P, Sutchritpongsa P, et al. Effects of delayed suprapubic port removal on post-laparoscopic shoulder pain: A randomized controlled trial. Gynecol Surg. 2021;18(1). doi: 10.1186/s10397-021-01087-6.
16. Chawla T, Shahzad N, Ahmad K, et al. Post-operative pain after laparoscopic ventral hernia repair, the impact of mesh soakage with bupivacaine solution versus normal saline solution: A randomised controlled trial (HAPPIEST Trial). J Minim Access Surg. 2020 Oct-Dec;16(4):328-34. doi: 10.4103/jmas.JMAS_50_19. PMID: 32978352.
17. Chen T, Li M, Liu Y, et al. A Prospective Randomized Controlled Study of Ultrasound-Guided Rectus Sheath Block for Pain Management in Laparoscopic Umbilical Hernia Repair with Intraperitoneal Onlay Mesh. J Pain Res. 2025;18:1-10. doi: 10.2147/jpr.S481853. PMID: 39776764.
18. Chowdhury TS, Mustary M, Chowdhury TA. A randomized controlled trial depicting postoperative pain score following port-site infiltration during laparoscopy. Journal of South Asian Federation of Obstetrics and Gynaecology. 2019;11(4):230-4. doi: 10.5005/jp-journals-10006-1693.
19. Clanet M, Touihri K, El Haddad C, et al. Effect of opioid-free versus opioid-based strategies during multimodal anaesthesia on postoperative morphine consumption after bariatric surgery: a randomised double-blind clinical trial. BJA Open. 2024 Mar;9:100263. doi: 10.1016/j.bjao.2024.100263. PMID: 38435809.
20. Collange V, Berruet JB, Aubrun F, et al. Opioid free versus opioid sparing strategies for multimodal antinociception during laparoscopic colectomy: a randomised controlled trial. Anaesthesia Critical Care & Pain Medicine. 2024;43(6):101436. doi: 10.1016/j.accpm.2024.101436. PMID: 39393528.
21. Cope AG, Wetzstein MM, Mara KC, et al. Abdominal Ice after Laparoscopic Hysterectomy: A Randomized Controlled Trial. J Minim Invasive Gynecol. 2021 Feb;28(2):342-50.e2. doi: 10.1016/j.jmig.2020.06.027. PMID: 32622918.
22. Cunningham TK, Draper H, Bexhell H, et al. A double-blinded randomised controlled study to investigate the effect of intraperitoneal levobupivacaine on post laparoscopic pain. Facts Views & Vision in Obgyn. 2020;12(3):155-61. PMID: 33123690.
23. Dai L, Ling X, Qian Y. Effect of Ultrasound-Guided Transversus Abdominis Plane Block Combined with Patient-Controlled Intravenous Analgesia on Postoperative Analgesia After Laparoscopic Cholecystectomy: a Double-Blind, Randomized Controlled Trial. Journal of gastrointestinal surgery : official journal of the Society for Surgery of the Alimentary Tract. 2022 Dec;26(1):2542-50. doi: 10.1007/s11605-022-05450-6. PMID: 36100826.
24. De Cicco Nardone C, Ficarola F, Plotti F, et al. The role of peritoneal lavage in benign gynecologic laparoscopic surgery. Eur Rev Med Pharmacol Sci. 2023;27(1):6800-8. doi: 10.26355/eurrev_202307_33151. PMID: 37522691.

25. El Chaar M, Stoltzfus J, Claros L, et al. IV Acetaminophen Results in Lower Hospital Costs and Emergency Room Visits Following Bariatric Surgery: a Double-Blind, Prospective, Randomized Trial in a Single Accredited Bariatric Center. Journal of gastrointestinal surgery : official journal of the Society for Surgery of the Alimentary Tract. 2016 Apr;20(4):715-24. doi: 10.1007/s11605-016-3088-0. PMID: 26842692.
26. El Sherif FA, Othman AH, Abd El-Rahman AM, et al. Effect of adding intrathecal morphine to a multimodal analgesic regimen for postoperative pain management after laparoscopic bariatric surgery: a prospective, double-blind, randomized controlled trial. British Journal of Pain. 2016 Nov;10(4):209-16. doi: 10.1177/2049463716668904. PMID: 27867510.
27. Fassoulaki A, Vassi E, Korkolis D, et al. Perioperative continuous ropivacaine wound infusion in laparoscopic cholecystectomy: A randomized controlled double-blind trial. Surgical Laparoscopy, Endoscopy and Percutaneous Techniques. 2016 Feb;26(1):25-30. doi: 10.1097/sle.0000000000000224. PMID: 26679680.
28. Friesgaard KD, Brix LD, Kristensen CB, et al. Clinical effectiveness and safety of intraoperative methadone in patients undergoing laparoscopic hysterectomy: a randomised, blinded clinical trial. BJA Open. 2023;7:100219. doi: 10.1016/j.bjao.2023.100219. PMID: 37638083.
29. Frost AS, Kohn JR, Le Neveu M, et al. Laparoscopic administration of bupivacaine at the uterosacral ligaments during benign laparoscopic and robotic hysterectomy: a randomized controlled trial. Am J Obstet Gynecol. 2023;229(5):526.e1-.e14. doi: 10.1016/j.ajog.2023.07.047. PMID: 37531986.
30. Geng Z, Bi H, Zhang D, et al. The impact of multimodal analgesia based enhanced recovery protocol on quality of recovery after laparoscopic gynecological surgery: a randomized controlled trial. BMC Anesthesiol. 2021;21(1):179. doi: 10.1186/s12871-021-01399-2. PMID: 34182929.
31. Grimsby GM, Conley SP, Trentman TL, et al. A double-blind randomized controlled trial of continuous intravenous Ketorolac vs placebo for adjuvant pain control after renal surgery. Mayo Clin Proc. 2012;87(1):1089-97. doi: 10.1016/j.mayocp.2012.07.018. PMID: 23058854.
32. Gu S, Lang H, Gan J, et al. Effect of transcutaneous electrical acupoint stimulation on gastrointestinal function recovery after laparoscopic radical gastrectomy - A randomized controlled trial. European Journal of Integrative Medicine. 2019;26:11-7. doi: 10.1016/j.eujim.2019.01.001.
33. Gungorduk K, Asicioglu O, Ozdemir IA. Effect of the pulmonary recruitment maneuver on pain after laparoscopic gynecological oncologic surgery: a prospective randomized trial. J Gynecol Oncol. 2018;29(6):e92. doi: 10.3802/jgo.2018.29.e92. PMID: 30207100.
34. Gungorduk K, Gulseren V, Tastan L, et al. Paracervical block before laparoscopic total hysterectomy: A randomized controlled trial. Taiwanese Journal of Obstetrics and Gynecology. 2024 Mar;63(2):186-91. doi: 10.1016/j.tjog.2024.01.013. PMID: 38485313.
35. Herrador-Benito J, Paramo-Zunzunegui J, Rodriguez-Caravaca G, et al. Pre-incisional local infiltration with levobupivacaine in laparoscopic cholecystectomy: a randomized and clinical trial. Cirugia y Cirujanos (English Edition). 2024;92(1):69-75. doi: 10.24875/ciru.230000221. PMID: 38537241.
36. Huynh TQ, Patel NR, Goldstein ND, et al. Preoperative Gabapentin for Minimally Invasive Hysterectomy: A Randomized Controlled Trial. J Minim Invasive Gynecol. 2021 Feb;28(2):237-44.e2. doi: 10.1016/j.jmig.2020.04.040. PMID: 32389735.

37. Ismail MT, Hassanin MZ, Elshmaa NS. Preincisional infiltration and intraperitoneal instillation of levobupivacaine 0.25% for management of early postoperative pain following laparoscopic ovarian drilling. J Obstet Gynaecol Res. 2013;39(2):543-8. doi: 10.1111/j.1447-0756.2012.01989.x. PMID: 368566252.
38. Jairath A, Ganpule A, Gupta S, et al. Can intraperitoneal bupivacaine decreases pain in patients undergoing laparoscopic live donor nephrectomy? A randomized control trial. World J Urol. 2017 Jun;35(6):985-9. doi: 10.1007/s00345-016-1942-z. PMID: 27678271.
39. Janjua S, Aslam KZ, Sarfraz S, et al. Comparison of modified unilateral ultrasound guided subcostal transversus abdominis plane block with conventional port-site and intraperitoneal infiltration of bupivacaine for postoperative pain relief in laparoscopic cholecystectomy. Pakistan Armed Forces Medical Journal. 2019;69(4):801-7.
40. Jiang R, Sun Y, Wang H, et al. Effect of different carbon dioxide (CO<inf>2</inf>) insufflation for laparoscopic colorectal surgery in elderly patients: A randomized controlled trial. Medicine (United States). 2019 Oct;98(4):e17520. doi: 10.1097/md.0000000000017520. PMID: 31593122.
41. Joudi M, Fathi M, Izanloo A, et al. An Evaluation of the Effect of Hypnosis on Postoperative Analgesia following Laparoscopic Cholecystectomy. Int J Clin Exp Hypn. 2016 Jul-Sep;64(3):365-72. doi: 10.1080/00207144.2016.1171113. PMID: 27267679.
42. Kamali A, Ashrafi TH, Rakei S, et al. A comparative study on the prophylactic effects of paracetamol and dexmedetomidine for controlling hemodynamics during surgery and postoperative pain in patients with laparoscopic cholecystectomy. Medicine. 2018;97(5):e13330. doi: 10.1097/md.0000000000013330. PMID: 30572436.
43. Kaur R, Seal A, Lemech I, et al. Intraperitoneal Instillation of Local Anesthetic (IPILA) in Bariatric Surgery and the Effect on Post-operative Pain Scores: a Randomized Control Trial. Obes Surg. 2022 Jul;32(7):2349-56. doi: 10.1007/s11695-022-06086-w. PMID: 35508748.
44. Kawahara R, Tamai Y, Yamasaki K, et al. The analgesic efficacy of ultrasound-guided transversus abdominis plane block with mid-axillary approach after gynecologic laparoscopic surgery: A randomized controlled trial. Journal of Anaesthesiology Clinical Pharmacology. 2015 Jan-Mar;31(1):67-71. doi: 10.4103/0970-9185.150547. PMID: 25788776.
45. Kerketta CS, Chhanwal HS, Kheskani D, et al. Opioid Free Anesthesia in Laparoscopic Surgery: A New Emerging Technique. Journal of Cellular and Molecular Anesthesia. 2023;8(4):256-63. doi: 10.22037/jcma.v8i3.41302.
46. Kheradkish F, Valiani M, Amini Rarani S, et al. The effect of acupressure on pain levels and physiologic indicator in patients undergoing laparoscopic cholecystectomy: A double-blind, randomized clinical trial. Complement Ther Clin Pract. 2024 Nov;57:101868. doi: 10.1016/j.ctcp.2024.101868. PMID: 38850620.
47. Kihlstedt Pasquier E, Andersson E. Pulmonary Recruitment Maneuver Reduces Shoulder Pain and Nausea After Laparoscopic Cholecystectomy: A Randomized Controlled Trial. World J Surg. 2021 Dec;45(1):3575-83. doi: 10.1007/s00268-021-06262-6. PMID: 34482412.
48. Kinjo Y, Kurita T, Fujino Y, et al. Evaluation of laparoscopic-guided rectus sheath block in gynecologic laparoscopy: A prospective, double-blind randomized trial. International Journal Of Surgery. 2019 Feb;62:47-53. doi: 10.1016/j.ijsu.2019.01.004. PMID: 30654145.

49. Klugsberger B, Schreiner M, Rothe A, et al. Warmed, humidified carbon dioxide insufflation versus standard carbon dioxide in laparoscopic cholecystectomy: A double-blinded randomized controlled trial. Surg Endosc. 2014;28(9):2656-60. doi: 10.1007/s00464-014-3522-x. PMID: 53171281.
50. Lee C, Chung JY, Lee M. Sex-related differences in the efficacy of dexamethasone pretreatment for postoperative analgesia in patients undergoing laparoscopic cholecystectomy: A randomized controlled study. Turkish Journal of Medical Sciences. 2017 Aug 23;47(4):1282-6. doi: 10.3906/sag-1701-113. PMID: 29156875.
51. Lee C, Chung JY, Lee M. Age-related differences in the efficacy of dexamethasone for postoperative analgesia in patients undergoing laparoscopic cholecystectomy: A randomised controlled study. Kuwait Med J. 2018;50(3):282-7.
52. Li X, Xu ZZ, Li XY, et al. The analgesic efficacy of ultrasound-guided transversus abdominis plane block for retroperitoneoscopic renal surgery: A randomized controlled study. BMC Anesthesiol. 2019 Oct 18;19(1):186. doi: 10.1186/s12871-019-0850-3. PMID: 31627728.
53. Lin S, Hua J, Xu B, et al. Comparison of bupivacaine and parecoxib for postoperative pain relief after laparoscopic cholecystectomy: A randomized controlled trial. Int J Clin Exp Med. 2015;8(8):13824-9. PMID: 26550332.
54. Liu X, Hu J, Hu X, et al. Preemptive Intravenous Nalbuphine for the Treatment of Post-Operative Visceral Pain: A Multicenter, Double-Blind, Placebo-Controlled, Randomized Clinical Trial. Pain and Therapy. 2021 Dec;10(2):1155-69. doi: 10.1007/s40122-021-00275-8. PMID: 34089152.
55. Lu H, Xie Q, Ye W, et al. Ultrasound-guided erector spinae plane block for postoperative analgesia in Chinese patients undergoing laparoscopic cholecystectomy: a double-blind randomized controlled trial. Langenbecks Arch Surg. 2023;408(1):111. doi: 10.1007/s00423-023-02834-3. PMID: 36854802.
56. Manan A, Khan AA, Ahmad I, et al. Intraperitoneal bupivacaine as post-laparoscopic cholecystectomy analgesia. Journal of the College of Physicians and Surgeons Pakistan. 2020 Jan;30(1):9-12. doi: 10.29271/jcpsp.2020.01.09. PMID: 31931924.
57. Mei W, Li M, Yu Y, et al. Tropisetron alleviate early post-operative pain after gynecological laparoscopy in sevoflurane based general anaesthesia: A randomized, parallel-group, factorial study. European Journal of Pain (United Kingdom). 2014;18(2):238-48. doi: 10.1002/j.1532-2149.2013.00365.x. PMID: 372578519.
58. Mohtadi A, Nesioonpour S, Salari A, et al. The effect of single-dose administration of dexamethasone on postoperative pain in patients undergoing laparoscopic cholecystectomy. Anesthesiology & Pain Medicine. 2014;4(3):e17872. doi: 10.5812/aapm.17872. PMID: 25237639.
59. Mongelli F, Marengo M, Bertoni MV, et al. Laparoscopic-Assisted Transversus Abdominis Plane (TAP) Block Versus Port-Site Infiltration with Local Anesthetics in Bariatric Surgery: a Double-Blind Randomized Controlled Trial. Obes Surg. 2023 Nov;33(1):3383-90. doi: 10.1007/s11695-023-06825-7. PMID: 37740830.
60. Nanda A, Sangineni KSD, Pakhare V, et al. Comparative Evaluation of Efficacy of Preventive Analgesia with Diclofenac and Buprenorphine Patch versus Single Diclofenac Patch for Postoperative Pain following General Anesthesia for Laparoscopic Cholecystectomy. Anesthesia: Essays and Researches. 2020;14(3):428-33. doi: 10.4103/aer.AER_109_20. PMID: 34092854.
61. Nazar PA, Davul A. Efficacy of Transmuscular Quadratus Lumborum Block in the Multimodal Regimen for Postoperative Analgesia after Total Laparoscopic Hysterectomy: A Prospective Randomised Double-Blinded Study. International Journal of Academic Medicine and Pharmacy. 2023;5(1):324-7. doi: 10.47009/jamp.2023.5.1.67.

62. Noh YJ, Kwon EJ, Bang YJ, et al. The effect of lung-recruitment maneuver on postoperative shoulder pain in patients undergoing laparoscopic cholecystectomy: a randomized controlled trial. J Anesth. 2024 Dec;38(6):839-47. doi: 10.1007/s00540-024-03403-8. PMID: 39276226.
63. Okam PC, Ikechebelu JI, Eleje GU, et al. Efficacy and safety of diclofenac suppository for postoperative pain relief after diagnostic hystero-laparoscopy and dye test: A double-blind, placebo-controlled, randomized trial. European Journal of Obstetrics and Gynecology and Reproductive Biology: X. 2024 Sep;23(n):100326. doi: 10.1016/j.eurox.2024.100326. PMID: 39104867.
64. Papasavas P, Seip RL, McLaughlin T, et al. A randomized controlled trial of an enhanced recovery after surgery protocol in patients undergoing laparoscopic sleeve gastrectomy. Surg Endosc. 2023 Feb;37(2):921-31. doi: 10.1007/s00464-022-09512-3. PMID: 36050610.
65. Park SE, Hong TH. Effects of extremely low-pressure pneumoperitoneum on postoperative recovery after single site robot-assisted cholecystectomy: a randomized controlled trial. Langenbecks Arch Surg. 2023;408(1):242. doi: 10.1007/s00423-023-02988-0. PMID: 37349518.
66. Pasquier EK, Andersson E. Pulmonary recruitment maneuver reduces pain after laparoscopic bariatric surgery: a randomized controlled clinical trial. Surg Obes Relat Dis. 2018 Mar;14(3):386-92. doi: 10.1016/j.soard.2017.11.017. PMID: 29290563.
67. Pedrazzani C, Park SY, Conti C, et al. Analgesic efficacy of pre-emptive local wound infiltration plus laparoscopic-assisted transversus abdominis plane block versus wound infiltration in patients undergoing laparoscopic colorectal resection: results from a randomized, multicenter, single-blind, non-inferiority trial. Surg Endosc. 2021 Jul;35(7):3329-38. doi: 10.1007/s00464-020-07771-6. PMID: 32632489.
68. Peng L, Song Y, Lv B, et al. The effect of implementation of pain neuroscience education and rehabilitation exercise on post-operative pain and recovery after laparoscopic colorectal surgery: a prospective randomized controlled trial. J Anesth. 2023 Oct;37(5):775-86. doi: 10.1007/s00540-023-03235-y. PMID: 37528250.
69. Perez JJ, Strunk JD, Preciado OM, et al. Effect of an opioid-free anesthetic on postoperative opioid consumption after laparoscopic bariatric surgery: A prospective, single-blinded, randomized controlled trial. Reg Anesth Pain Med. 2024 Jun 5(n). doi: 10.1136/rapm-2024-105632. PMID: 38839427.
70. Prabhakaran S, Misra S, Magila M, et al. Randomized Controlled Trial Comparing the Outcomes of Enhanced Recovery After Surgery and Standard Recovery Pathways in Laparoscopic Sleeve Gastrectomy. Obes Surg. 2020 Sep;30(9):3273-9. doi: 10.1007/s11695-020-04585-2. PMID: 32291702.
71. Pushparani A, Venkatraman R, Gayathri L, et al. Efficacy of Single Dose Oral Gabapentin in Day Care Laparoscopic Surgeries-A Randomized Double-blinded Placebo Control Study. International Journal of Research in Pharmaceutical Sciences. 2022;13(3):311-7. doi: 10.26452/ijrps.v13i3.2228.
72. Radosa JC, Radosa MP, Mavrova R, et al. Five minutes of extended assisted ventilation with an open umbilical trocar valve significantly reduces postoperative abdominal and shoulder pain in patients undergoing laparoscopic hysterectomy. European Journal of Obstetrics and Gynecology and Reproductive Biology. 2013;171(1):122-7. doi: 10.1016/j.ejogrb.2013.08.014. PMID: 52754660.

73. Radosa JC, Radosa MP, Schweitzer PA, et al. Impact of different intraoperative CO2 pressure levels (8 and 15 mmHg) during laparoscopic hysterectomy performed due to benign uterine pathologies on postoperative pain and arterial pCO2 : a prospective randomised controlled clinical trial. BJOG. 2019;126(1):1276-85. doi: 10.1111/1471-0528.15826. PMID: 31136069.
74. Ren L, Qin P, Min S, et al. Transversus Abdominis Plane Block Versus Local Wound Infiltration for Postoperative Pain After Laparoscopic Colorectal Cancer Resection: a Randomized, Double-Blinded Study. J Gastrointest Surg. 2022;26(2):425-32. doi: 10.1007/s11605-021-05121-y. PMID: 34505222.
75. Rindos NB, Mansuria SM, Ecker AM, et al. Intravenous acetaminophen vs saline in perioperative analgesia with laparoscopic hysterectomy. Am J Obstet Gynecol. 2019 Apr;220(4):373.e1-.e8. doi: 10.1016/j.ajog.2019.01.212. PMID: 30682359.
76. Ruiz-Tovar J, Garcia A, Ferrigni C, et al. Application of Vitamin E Acetate on Staple Lines and Anastomoses of Roux-en-Y Gastric Bypass: Impact on Postoperative Pain and Acute Phase Reactants. Obes Surg. 2020;30(8):2988-93. doi: 10.1007/s11695-020-04635-9. PMID: 32342266.
77. Ruiz-Tovar J, Garcia A, Ferrigni C, et al. Impact of implementation of an enhanced recovery after surgery (ERAS) program in laparoscopic Roux-en-Y gastric bypass: a prospective randomized clinical trial. Surg Obes Relat Dis. 2019 Feb;15(2):228-35. doi: 10.1016/j.soard.2018.11.002. PMID: 30606469.
78. Ruiz-Tovar J, Garcia A, Ferrigni C, et al. Laparoscopic-Guided Transversus Abdominis Plane (TAP) Block as Part of Multimodal Analgesia in Laparoscopic Roux-en-Y Gastric Bypass Within an Enhanced Recovery After Surgery (ERAS) Program: a Prospective Randomized Clinical Trial. Obes Surg. 2018 Nov;28(1):3374-9. doi: 10.1007/s11695-018-3376-8. PMID: 29980989.
79. Ruiz-Tovar J, Gonzalez G, Sarmiento A, et al. Analgesic effect of postoperative laparoscopic-guided transversus abdominis plane (TAP) block, associated with preoperative port-site infiltration, within an enhanced recovery after surgery protocol in one-anastomosis gastric bypass: a randomized clinical trial. Surg Endosc. 2020 Dec;34(1):5455-60. doi: 10.1007/s00464-019-07341-5. PMID: 31932932.
80. Ruiz-Tovar J, Gonzalez J, Garcia A, et al. Intraperitoneal Ropivacaine Irrigation in Patients Undergoing Bariatric Surgery: a Prospective Randomized Clinical Trial. Obes Surg. 2016 Nov;26(1):2616-21. doi: 10.1007/s11695-016-2142-z. PMID: 27007272.
81. Rupniewska-Ladyko A, Malec-Milewska M, Kraszewska E, et al. Gabapentin before laparoscopic sleeve gastrectomy reduces postoperative oxycodone consumption in obese patients: A randomized double-blind placebo-controlled trial. Minerva Anestesiol. 2018 May;84(5):565-71. doi: 10.23736/s0375-9393.17.12194-2. PMID: 29108405.
82. Safari S, Rokhtabnak F, Djalali Motlagh S, et al. Effect of intraperitoneal bupivacaine on postoperative pain in laparoscopic bariatric surgeries. Surg Obes Relat Dis. 2020 Feb;16(2):299-305. doi: 10.1016/j.soard.2019.10.028. PMID: 31836291.
83. Salmonsen CB, Lange KHW, Kleif J, et al. Transversus abdominis plane block in minimally invasive colon surgery: A multicenter three-arm randomized controlled superiority and non-inferiority clinical trial. Regional Anesthesia and Pain Medicine. 2024 Jan 23. doi: 10.1136/rapm-2024-105712. PMID: 39542642.

84. Schipper IE, Schouten M, Yalcin T, et al. The Use of Intraperitoneal Bupivacaine in Laparoscopic Roux-en-Y Gastric Bypass: a Double-blind, Randomized Controlled Trial. Obes Surg. 2019 Oct;29(1):3118-24. doi: 10.1007/s11695-019-03982-6. PMID: 31201692.
85. Sherif AA, Elsersy HE. The impact of dexmedetomidine or xylocaine continuous infusion on opioid consumption and recovery after laparoscopic sleeve gastrectomy. Minerva Anestesiol. 2017;83(1):1274-82. doi: 10.23736/s0375-9393.17.11855-9. PMID: 28607333.
86. Singla S, Mittal G, Raghav, et al. Pain management after laparoscopic cholecystectomy-a randomized prospective trial of low pressure and standard pressure pneumoperitoneum. Journal of Clinical and Diagnostic Research JCDR. 2014;8(2):92-4. doi: 10.7860/jcdr/2014/7782.4017. PMID: 24701492.
87. Sinha A, Jayaraman L, Punhani D. Efficacy of ultrasound-guided transversus abdominis plane block after laparoscopic bariatric surgery: A double blind, randomized, controlled study. Obes Surg. 2013;23(4):548-53. doi: 10.1007/s11695-012-0819-5. PMID: 52432056.
88. Sutton E, Bellini G, Grieco MJ, et al. Warm and Humidified Versus Cold and Dry CO<inf>2</inf> Pneumoperitoneum in Minimally Invasive Colon Resection: A Randomized Controlled Trial. Surg Innov. 2017 Oct;24(5):471-82. doi: 10.1177/1553350617715834. PMID: 28653583.
89. Terranova C, Schiavoni L, Ficarola F, et al. The Role of TAP/RS Block in Minor Gynecologic Laparoscopic Surgery: A Randomized Clinical Trial. Gynecol Obstet Invest. 2024;89(2):103-10. doi: 10.1159/000535835. PMID: 38266505.
90. Tsai HW, Wang PH, Yen MS, et al. Prevention of postlaparoscopic shoulder and upper abdominal pain: A randomized controlled trial. Obstet Gynecol. 2013;121(3):526-31. doi: 10.1097/AOG.0b013e318283fcca. PMID: 368734054.
91. Tülübaş EK, Seyit H, Bostancı İ, et al. Laparoscopic Transversus Abdominal Plane Block is Effective in Multimodal Analgesia for Laparoscopic Sleeve Gastrectomy. Medical Journal of Bakirkoy. 2019;15(3):198-203. doi: 10.4274/BTDMJB.galenos.2018.20180528093003.
92. Turner LC, Zyczynski HM, Shepherd JP. Intravenous Acetaminophen Before Pelvic Organ Prolapse Repair: A Randomized Controlled Trial. Obstet Gynecol. 2019;133(3):492-502. doi: 10.1097/aog.0000000000003102. PMID: 30741813.
93. Wang Y, Sun J, Yu K, et al. Virtual reality exposure reduce acute postoperative pain in female patients undergoing laparoscopic gynecology surgery: A Randomized Control Trial (RCT) study. J Clin Anesth. 2024;97:111525. doi: 10.1016/j.jclinane.2024.111525. PMID: 38870701.
94. Wong KA, Cabrera AG, Argiroff AL, et al. Transversus abdominis plane block with liposomal bupivacaine and its effect on opiate use after weight loss surgery: a randomized controlled trial. Surg Obes Relat Dis. 2020 Jul;16(7):886-93. doi: 10.1016/j.soard.2020.03.031. PMID: 32402732.
95. Xue Q, Chu Z, Zhu J, et al. Analgesic Efficacy of Transverse Abdominis Plane Block and Quadratus Lumborum Block in Laparoscopic Sleeve Gastrectomy: A Randomized Double-Blinded Clinical Trial. Pain and Therapy. 2022 Jun;11(2):613-26. doi: 10.1007/s40122-022-00373-1. PMID: 35312948.
96. Yao L, Wang Y, Du B, et al. Comparison of Postoperative Pain and Residual Gas between Restrictive and Liberal Fluid Therapy in Patients Undergoing Laparoscopic Cholecystectomy. Surgical Laparoscopy, Endoscopy and Percutaneous Techniques. 2017 Oct;27(5):346-50. doi: 10.1097/sle.0000000000000463. PMID: 28891830.

97. Yin G, Li Y, Wei P, et al. Analgesic effect of the ultrasound-guided thoracolumbar paravertebral block in patients undergoing robot-assisted laparoscopic nephrectomy: a randomized controlled trial. BMC Anesthesiol. 2024;24(1):69. doi: 10.1186/s12871-024-02460-6. PMID: 38388893.
98. Yu JM, Tao QY, He Y, et al. Opioid-Free Anesthesia for Pain Relief After Laparoscopic Cholecystectomy: A Prospective Randomized Controlled Trial. J Pain Res. 2023;16:3625-32. doi: 10.2147/jpr.S432601. PMID: 37928062.
99. Zeeni C, Chamsy D, Khalil A, et al. Effect of postoperative Trendelenburg position on shoulder pain after gynecological laparoscopic procedures: a randomized clinical trial. BMC Anesthesiol. 2020;20(1):27. doi: 10.1186/s12871-020-0946-9. PMID: 31996139.
100. Zhang H, Du G, Liu YF, et al. Overlay of a sponge soaked with ropivacaine and multisite infiltration analgesia result in faster recovery after laparoscopic hepatectomy. World J Gastroenterol. 2019 Sep 14;25(3):5185-96. doi: 10.3748/wjg.v25.i34.5185. PMID: 31558866.
101. Zheng LQ, Kosai NR, Ani MFC, et al. The Impact of Laparoscopic Intraperitoneal Instillation of Ropivacaine in Enhancing Respiratory Recovery and Reducing Acute Postoperative Pain in Laparoscopic Sleeve Gastrectomy: a Double-Blinded Randomised Control; RELiEVE Trial. Obes Surg. 2023 Oct;33(1):3141-6. doi: 10.1007/s11695-023-06777-y. PMID: 37667104.
